# Shaping Antimalarials: A Geometry-First Approach to *Pf*CLK3 Covalent Inhibitors

**DOI:** 10.1021/acs.jmedchem.5c03342

**Published:** 2026-01-31

**Authors:** Skye B. Brettell, Carla Fuentes-Guerra Bustos, Saumya Sharma, Gillian Cann, Lauren V. Carruthers, Abbey Begen, Graeme Milligan, David J. Clarke, Andrew B. Tobin, Andrew G. Jamieson

**Affiliations:** † School of Chemistry, The Advanced Research Centre, University of Glasgow, 11 Chapel Lane, Glasgow G11 6EW, U.K.; ‡ Centre for Translational Pharmacology, The Advanced Research Centre, University of Glasgow, 11 Chapel Lane, Glasgow G11 6EW, U.K.; § Keltic Pharma Therapeutics, The Advanced Research Centre, University of Glasgow, 11 Chapel Lane, Glasgow G11 6EW, U.K.; ∥ EaSTCHEM School of Chemistry, University of Edinburgh, Joseph Black Building, David, Brewster Road, Edinburgh EH9 3FJ, U.K.

## Abstract

The emergence of*Plasmodium falciparum*resistance to frontline therapies highlights the urgent need for new antimalarial agents. The essential, multistage kinase *Pf*CLK3 is a validated target, and covalent kinase inhibitors (CKIs) offer potential for durable inhibition. However, CKI design has largely prioritised warhead reactivity over the geometric requirements which govern covalent bond formation. Herein, we describe a geometry-first approach to optimize covalent *Pf*CLK3 inhibitors, starting from the highly reactive chloroacetamide SB4–17 (**2**). Systematic variation of warhead and linker geometry revealed that maintaining the α-reactive geometry of the chloroacetamide scaffold enables covalent engagement of Cys368 with substantially less reactive electrophiles. Notably, the methyl sulfamate analogue SB5–171 (**14**) showed potent antiparasitic activity (EC_50_ = 104 nM) and improved metabolic stability (*t*_1/2_ = 35 min in mouse hepatocytes). These findings demonstrate that geometric optimization can decouple covalent engagement from high intrinsic reactivity, providing a rational framework for designing selective, drug-like CKIs.

## Graphical abstract

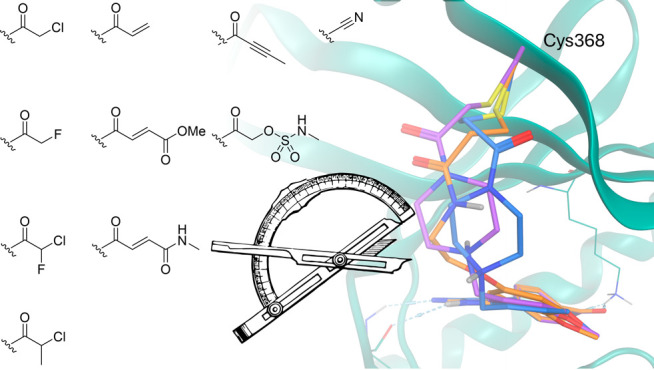

## Introduction

Drug resistance poses one of the biggest threats to human health in the modern world. Predictions forecast 40 million deaths from drug-resistant infections by 2050.^1–3^ Malaria is one such disease continuously evolving resistance to frontline therapeutics, with mortalities rising by 46% from 2019 to 2023.^4,5^ *Plasmodium falciparum*­(*Pf*) resistance to Artemisinin-based Combination Therapy (ACT), mediated, at least in part, by the *Pf*Kelch 13 protein is particularly troubling.^6^ The spread of resistant parasites to southeast Asia to mainland Africa now threatens to undo the progress of the last 25 years.^5^ While the recent introduction of the RTS,S and R21 vaccines represent a breakthrough, efficacy in the field has proved limited.^7,8^ Pilot schemes in young children, who account for the majority of malaria mortality, showed only a 13% reduction in mortality when used generally, and a 44.5% reduction when used seasonally. However, when used in conjunction with mosquito bed nets and effective chemoprevention, mortality was reduced by 95%. Widespread simultaneous rollout of seasonal vaccination and control interventions in malaria endemic countries, mainly of low-middle income status, is however logistically and financially challenging. This underscores the desperate need for novel chemotherapies to combat the rise of antimalarial resistance.

A novel mechanism of action that has demonstrated efficacy in both preclinical and clinical settings is the inhibition of kinases.^9^ Therapeutics targeting kinases have achieved considerable success in the treatment of oncology and autoimmune disorders.^10^ One such compound in the field of malaria, MMV390048 (**3**, Figures 1 and 3), is an inhibitor of*P. falciparum* phosphatidylinositol 4-kinase, and exhibited promising efficacy in Phase II clinical trials before development was discontinued due to the emergence of teratogenic effects in nonclinical studies involving rats.^11,12^ Despite these off-target toxicities, MMV390048 (**3**) provided a critical proof of concept, establishing that kinase inhibitors can be effectively employed for the treatment of malaria in a clinical setting.

**Figure 1:**
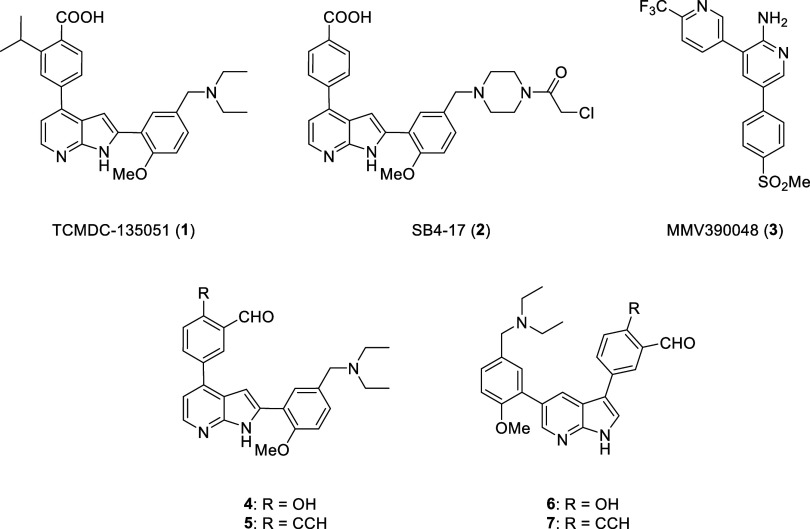
A selection of malarial kinase inhibitors in the literature.^2,13,15,16^

Our lab has been pursuing the essential protein kinase *Pf* cyclin dependent-like kinase 3 (*Pf*CLK3) as a potential new target. A high throughput screen of 13,533 compounds yielded TCMDC-135051 (**1**), a highly potent inhibitor of *Pf*CLK3 with nanomolar efficacy in liver, sexual and asexual blood stage parasites.^13^ The hit compound also cleared*Plasmodium berghei*­(*Pb*) parasites in mice when intraperitoneally dosed twice daily at 50 mg/kg. SAR studies and homology modeling suggested TCMDC-135051 (**1**) functions as a type-I kinase inhibitor, binding the ATP pocket of *Pf*CLK3.^14^ This was then confirmed by the X-ray cocrystal structure.^2^ Our recent efforts have focused on optimizing the efficacy and selectivity of TCMDC-135051 (**1**) by way of covalent kinase inhibitors (CKIs). Identification of a cysteine (Cys368) next to the *Pf*CLK3 ATP-binding site which is not well conserved across the human kinome enabled development of chloroacetamide **2**, SB4–17.^2^ This molecule demonstrated covalent inhibition of recombinant kinase, an extended duration of action in parasites, and a superior selectivity profile to that of TCMDC-135051 (**1**). We have also explored the targeting of the catalytic lysine as a novel strategy to evade future resistance mechanisms, developing a series of aldehyde-based warheads (compounds **4**–**7**).^15^

Despite the significant improvements to the selectivity and potency of our series, designing a covalent inhibitor with a good pharmacokinetic profile can be challenging. Electrophilic warheads suffer from intrinsic instability to metabolism, specifically by conjugation with glutathione and hydrolysis.^17,18^ In the present study, we aimed to optimize SB4–17 (**2**), a covalent inhibitor bearing a highly reactive and intrinsically unstable chloroacetamide warhead.^2^ While the literature extensively outlines the importance of tuning down warhead reactivity during the optimization of a covalent inhibitor, the importance of warhead geometry is not so apparent.^19–23^ Through systematic variation of warhead and linker geometry, we established a geometry-driven structure–activity relationship (SAR) that delivered multiple lead compounds with improved stability and potency relative to SB4–17 (**2**). Among these, SB5–171 (**14**), incorporating a methyl sulfamate warhead, achieved an optimal balance of antiplasmodial potency and metabolic stability in glutathione, human serum, and mouse hepatocytes. This work underscores the value of a geometry-first optimization strategy in covalent drug design and provides a promising lead compound, SB5–171 (**14**), which is currently undergoing evaluation in our *in vivo* malaria models. Collectively, these findings highlight a novel approach to covalent inhibitor development with potential to advance antimalarial drug discovery.

## Results

### Structure Based Drug Design

Chloroacetamides are highly reactive and metabolically labile.^2,17^ While excellent warheads for tool compounds, in covalent drug discovery, it is important to employ the least reactive warhead which will still engage the target residue, in order to avoid off-target effects and high clearance by metabolism. For this reason, the acrylamide warhead is generally regarded as the gold standard in covalent drug discovery due to its low intrinsic reactivity and specificity for cysteine.^20,24^ Previous work demonstrated that acrylamide-based warheads were unable to bind *Pf*CLK3 covalently, which was attributed to their lower reactivity relative to chloroacetamide SB4–17 (**2**). While traditional literature has focused solely on reactivity differences between warheads, a small number of recent studies have highlighted the importance of reaction geometry in covalent inhibitor design.^17,18,22,25,26^ The cysteine thiolate reacts with the chloroacetamide α-carbon (Figure 2), whereas it is the β-carbon is attacked on acrylamides. We hypothesized that the reactivity of our warhead could be tuned down significantly if the α-reactive geometry of chloroacetamide SB4–17 (**2**) was matched. As the scaffold of the molecule greatly influences warhead reactivity, matched molecular pairs should be compared whenever possible.^6,26^ Given the limited comparable data between all warheads employed in this current study, we have chosen to use the fold-change in glutathione half-life from a matched chloroacetamide as a metric with which to compare relative stabilities of warheads from different literature sources.^4–6,9^ We first sought to replace the chloroacetamide warhead with less reactive haloacetamides. Fluoroacetamides (compound **8**) are relatively inert due to the fluoride ion’s poor leaving group ability, however these warheads have been shown to react with cysteines when positioned in optimal proximity.^27^ Dihaloacetamide warheads were developed by Ojida, Shindo and colleagues in 2023 as α-reactive electrophiles with tunable reactivity according to the halides employed.^18,28^ Covalent adduct formation is also reversible, which has been shown to improve the toxicity profile of covalent compounds.^28^ Compound **9** features a chlorofluoroacetamide, which has proven approximately 61-fold less reactive to glutathione than the chloroacetamide using matched molecular pair analysis.^18^ Finally, the steric hindrance of the α-methyl chloroacetamides mean that this warhead (featured in compound **10**) is around 6-fold less reactive than its unsubstituted equivalents.^17^

**Figure 2:**
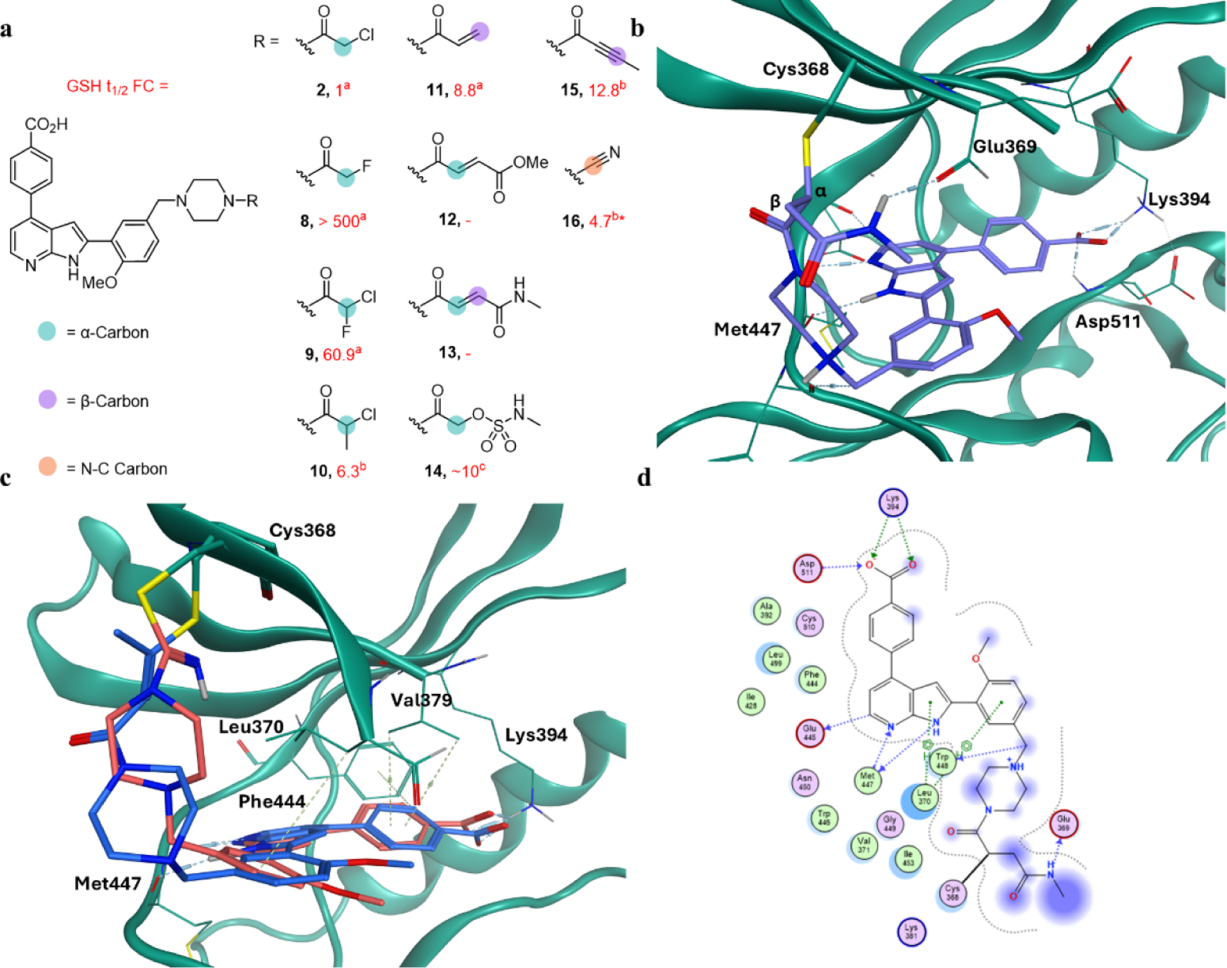
Novel designs featuring varied warheads. (a) Warheads chosen with α-reactive (teal), β-reactive (lilac) and N–C-carbon-reactive (orange) carbons. The fold-change of their half-lives in the presence of glutathione (GSH *t*_1/2_ FC) relative to the chloroacetamide is given in red. *t*_1/2_ value taken from reference a,^2,18^ reference b,^17^ and reference c.^32^ (b) Molecular docking of fumaramide **13** (lilac) in *Pf*CLK3 (teal, PDB: 8RPC), with the ligand interaction map shown in (d). Covalent adduct formation is predicted at the piperazine acrylamide’s α-carbon. (c) Molecular docking of butynamide **15** (blue) and cyanamide **16** (pink) in *Pf*CLK3 (teal). *Exact matched molecular pair not found in literature.

Next, the fumarate ester electrophiles developed by Cravatt and co-workers were employed (compound **12**, Figure 2).^29^ Like the acrylamides, these warheads are α,β-unsaturated Michael acceptors, however due to the presence of the more reactive acrylate, they are α-reactive relative to the acrylamide moiety (teal, Figure 2).^30^ Their intrinsic susceptibility to esterases confers kinetic selectivity: off-target reactivity was deemed to be slower than hydrolysis and inactivation by esterases, leading to improved selectivity among ibrutinib analogues.^29,31^ Fumaramide **13** was also designed to remove the esterase liability of these analogues to increase stability. In compound **13**, both α and β carbons should be reactive, though the secondary acrylamide (carbon highlighted in teal, Figure 2) should react with thiolates faster than the tertiary acrylamide.^17^ When fumaramide **13** was docked covalently into the cocrystal structure of TCMDC-135051 (**1**) and *Pf*CLK3 (PDB: 8RPC) using the molecular operating environment (MOE), this analogue maintained the binding mode of TCMDC-135051 (**1**). A bidentate hinge binding interaction with the azaindole core was predicted, as well as a salt bridge with the catalytic lysine, as is observed in the crystal structure. Interestingly, only the secondary acrylamide was shown to react with Cys368. The algorithm in MOE does not take into account reactivity differences, therefore this docking result speaks to this present hypothesis, that the α-carbon on this scaffold is the optimal position to react with Cys368.

Sulfamate acetamide warheads were then explored. In 2023, London and colleagues developed a series of electrophiles which employed sulfamates as leaving groups which break down into sulfur trioxide and an amine.^32^ These α-reactive warheads are highly tunable according to the substitution of the amine. The methyl sulfamate was shown to have comparable reactivity to that of the acrylamides, while matching the geometry of the chloroacetamide. For this reason, analogue **14** was designed. Finally, butynamide **15** and cyanamide **16** were designed. Despite having hugely different geometries to that of the acetamides or acrylamides, both docked well in *Pf*CLK3, maintaining the binding mode of other analogues while forming a covalent bond with Cys368.

The geometry of the covalent reaction was then varied further by diversifying the shape and length of the linker, using the acrylamide warhead (Figure 3a). First, the linker of the original scaffold was systematically modified, using piperazine (linkers A and B), pyrazolidine (linker C), and imidazolidine (linker D). While commercially available as mono-*Boc* diamines, the potential for instability among these linkers upon acylation was noted. Next, diazetidine (linker E), azetidine (linker F), and spirocyclic linkers G and H were designed. These designs were chosen from the Enamine spirocycle library. Finally, a linear ethanolamine linker I was designed to build in more flexibility with the hope of achieving the optimal angle of approach for the warhead. All designs were then docked into *Pf*CLK3, with those which docked favorably shown in Figure 3b–d. Linkers A, C, D, F, and I were predicted to maintain the binding mode of TCMDC-135051 (**1**), while covalently binding Cys368. Their docking scores and conformation energies are given in Figure 3e. All analogues scored well, however compounds **17** and **25** were predicted to have the most favorable conformational energies. Acrylamides **17**, **19**, **20**, **22** and **25** were therefore taken forward as synthetic targets. Linkers B, E, G, and H however were unable to maintain the binding mode of other analogues while engaging Cys368, which indicates that these designs do not achieve a favorable angle of approach to the nucleophile from the ATP binding-site. Analogues **18**, **21**, **23** and **24** were therefore deprioritised as synthetic targets.

**Figure 3:**
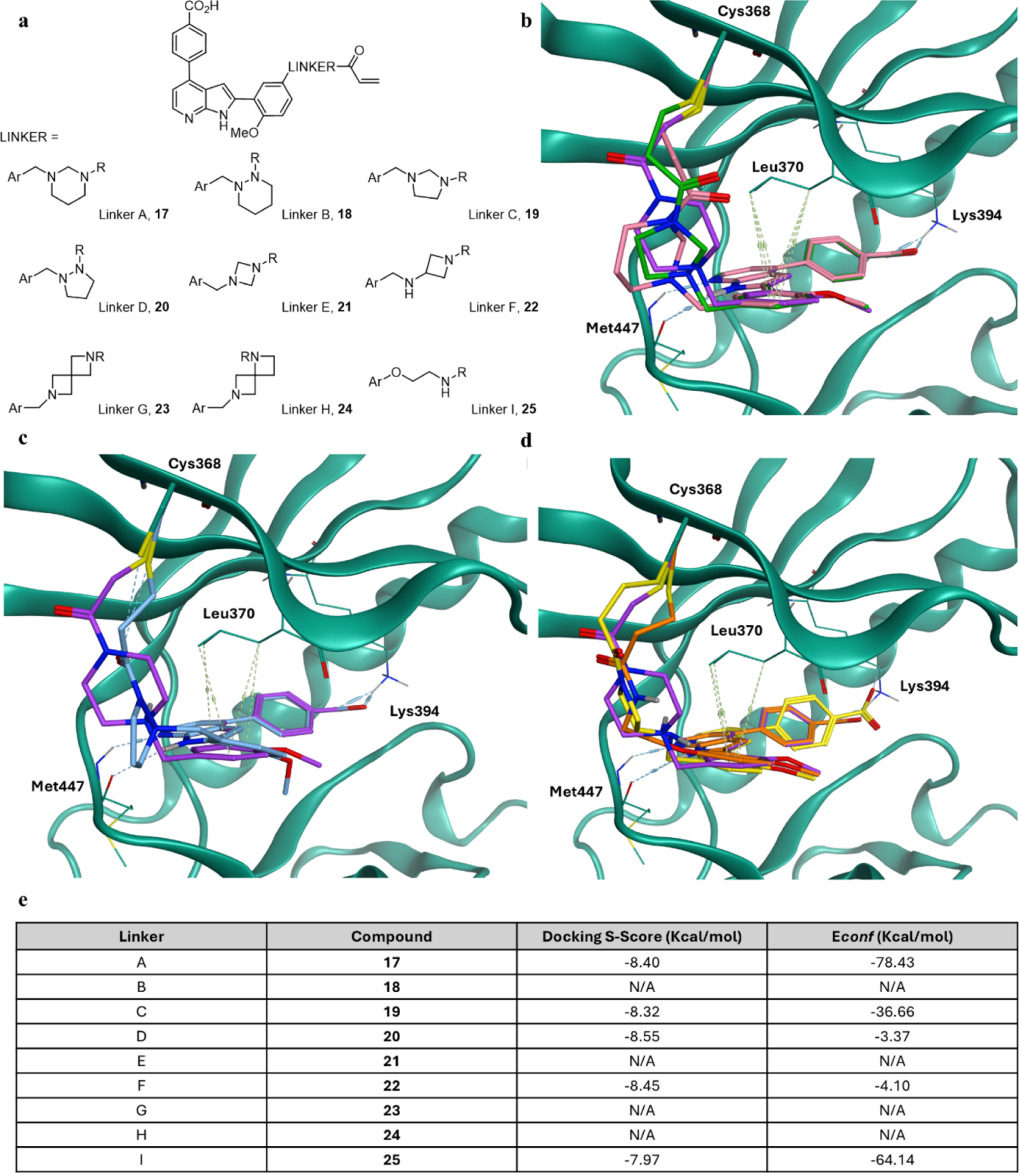
Variable linker and warhead designs. (b,c) Molecular docking of 1,3-piperazine **17** (pink), imidazolidine **19** (green) and pyrazolidine acrylamide **20** (blue) acrylamides as well as chloroacetamide **2** (lilac) in *Pf*CLK3 (teal, PDB: 8RPC). (d) Molecular docking of azetidine **22** (yellow), and ethanolamine **25** (orange) acrylamides as well as chloroacetamide **2** (lilac) in *Pf*CLK3 (teal). (e) Docking scores for all designs. N/A represents designs where binding-mode of TCMDC-135051 (**1**) was not maintained.

### Synthesis

Analogues **8**–**16** were obtained via the 8-step synthesis outlined in our previous work.^2^ From common intermediate **26**, late stage installation of warheads was possible (Scheme 1). The α-haloacetamides proceeded with mixed success: direct coupling with ethyl 2-fluoroacetate as per literature precedent afforded no conversion to product, therefore a two-step one pot procedure was carried out.^33,34^ First, the coupling of intermediate **26** and chloroacetyl chloride regenerated SB4–17 (**2**) in situ, before addition of TBAF to invoke halogen-exchange. This afforded **8** in a low yield of 5% after HPLC purification, with the predominant product being that of hydrolysis of the carbon–halogen bond. Next, coupling the racemic chlorofluoroacetic acid using T3P indicated only 25% conversion to product, affording **9** in 9% yield. The α-methyl chloroacetamide analogue **10** was obtained using 2-chloropropionyl chloride to install the warhead in 31% yield.

**Scheme 1:**
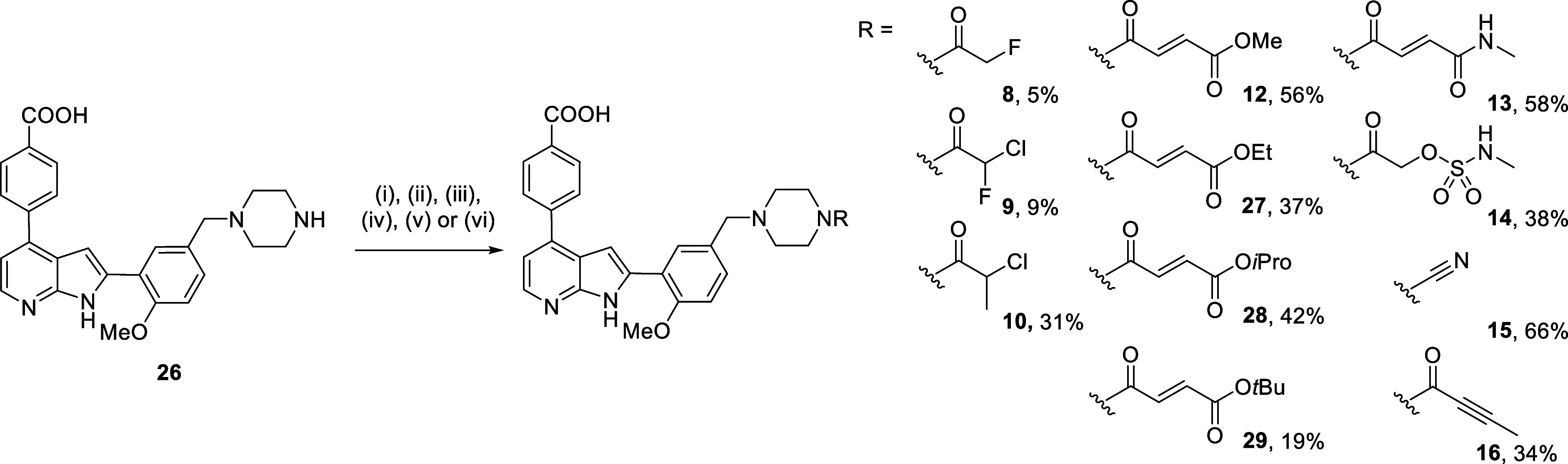
Late-Stage Diversification of Intermediate **26** to Afford Analogues **8**–**16** and **27**–**29** ^a^

a (i) Chloroacetyl chloride, DIPEA, DMF, r.t., 1 h followed by TBAF, r.t., 1 h. (ii) Acid, T3P, DIPEA, r.t., 1–36 h. (iii) Acid chloride, DIPEA, r.t., 3 h. (iv) Acid, HATU, DIPEA, r.t., 1–18 h (v) conditions (iv) followed by CsF, DMF, 80 °C, 6 h, followed by methylsulfamoyl chloride, DIPEA, DMF, r.t., 18 h. (vi) CNBr, NaHCO_3_ (aq), DMF, r.t., 18 h.

Fumarate esters **12** and **13** were obtained using HATU and DIPEA to couple the relevant carboxylic acids to amine **26**, affording analogues in 56% and 58% yield, respectively. Subsequently, fumarates **27**–**29** were also synthesized in 19–42% yield, inspired by recent work by Zaro and colleagues showing that increasing the steric bulk of the ester can increase the selectivity and stability of ibrutinib analogues.^31^ Butynamide **15** was obtained using T3P to couple **26** with 2-butynoic acid in 34% yield. For cyanamide **16**, the cyano group was installed using cyanogen bromide and sodium hydrogen carbonate yielding the desired product in 66% yield. As reverse-phase purification used acidic eluents, substoichiometric amounts of cyanogen bromide were used and the waste was neutralized using NaOH to prevent the release of hydrofluoric acid.

For the synthesis of the linker series, the published route was adapted to allow late-stage linker installation. From previously published intermediate **30**, the northern hemisphere of the molecule was appended using a Suzuki–Miyaura coupling, affording aldehyde **32** in 29% yield (Scheme 2). Linkers A, C, D and F were then installed using mono-*Boc* protected diamines via a series of reductive aminations. Compounds **33**–**36** were obtained in 65–89% yield.

**Scheme 2:**
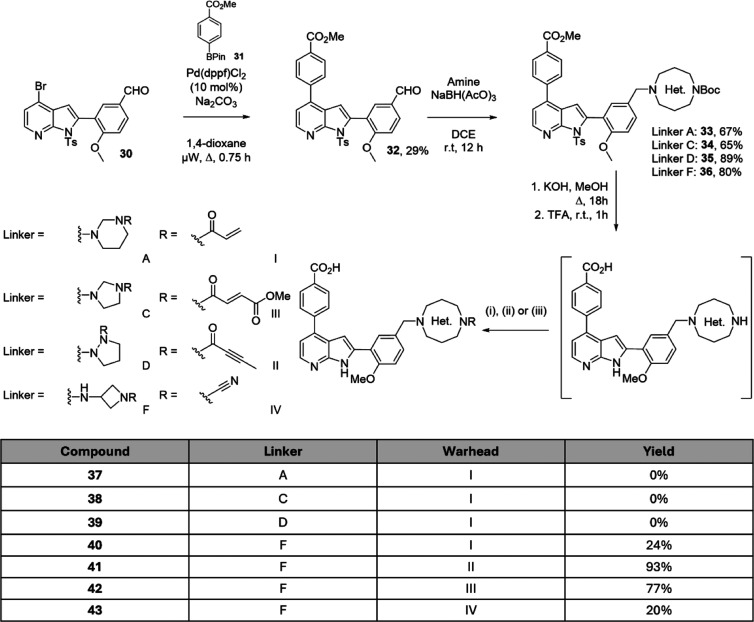
Synthesis of Analogues **37**–**43** (i) Acryloyl Chloride, DIPEA, DMF, r.t., 1 h^a^

a (ii) Carboxylic acid, T3P, DIPEA, DMF, r.t., 18 h. (iii) CNBr, NEt_3_, DMF, r.t., 18 h. Het = heterocycle, linker A, C, D, F.

A two-step deprotection strategy, as outlined in previous work, was applied to all analogues but achieved limited success (Scheme 2).^2^ After successful removal of the tosyl and ester protecting groups using K_2_CO_3_ in MeOH, subsequent treatment with TFA resulted in decomposition for linkers A and C. It was hypothesized that both heterocycles were able to ring-open under acidic conditions, similar to that of an acetal deprotection. Compounds **37** and **38** were therefore not obtained. For linker D, acylation of the fully deprotected free amine intermediate also resulted in decomposition via an unknown mechanism. Compound **39** was also not isolated.

For azetidine linker F, deprotection and acylation was more successful, and treatment of the free amine with acryloyl chloride and DIPEA resulted in a 24% yield of compound **40** (Scheme 2). In order to vary the geometry further, fumarate **41**, butynamide **42** and cyanamide **43** were also isolated using the same conditions outlined earlier.

For the synthesis of analogues featuring the linear linker I, a separate synthesis was undertaken (Scheme 3). N-*Boc*-ethanolamine (**44**) was appended to 3-bromo-4-methoxyphenol (**46**) via the mesylate intermediate (**45**) to give bromide **47** in 49% yield over two steps. A Miyaura borylation afforded boronic ester **48** in 48% yield. Two sequential Suzuki–Miyaura cross couplings installed both arms of the molecule to the 2-iodo-4-bromoazaindole **49** in 90% and 74% yield respectively to give **51**. Global deprotection then afforded intermediate **52**. The acrylamide and methyl fumarate **53** and **54** were obtained in 30% and 22% yield respectively, while butynamide and cyanamide **55** and **56** were not isolated. This was thought to be owing to the low reactivity of the ethanolamine linker, as only starting material was recovered in the synthesis of **55** and **56**.

**Scheme 3:**
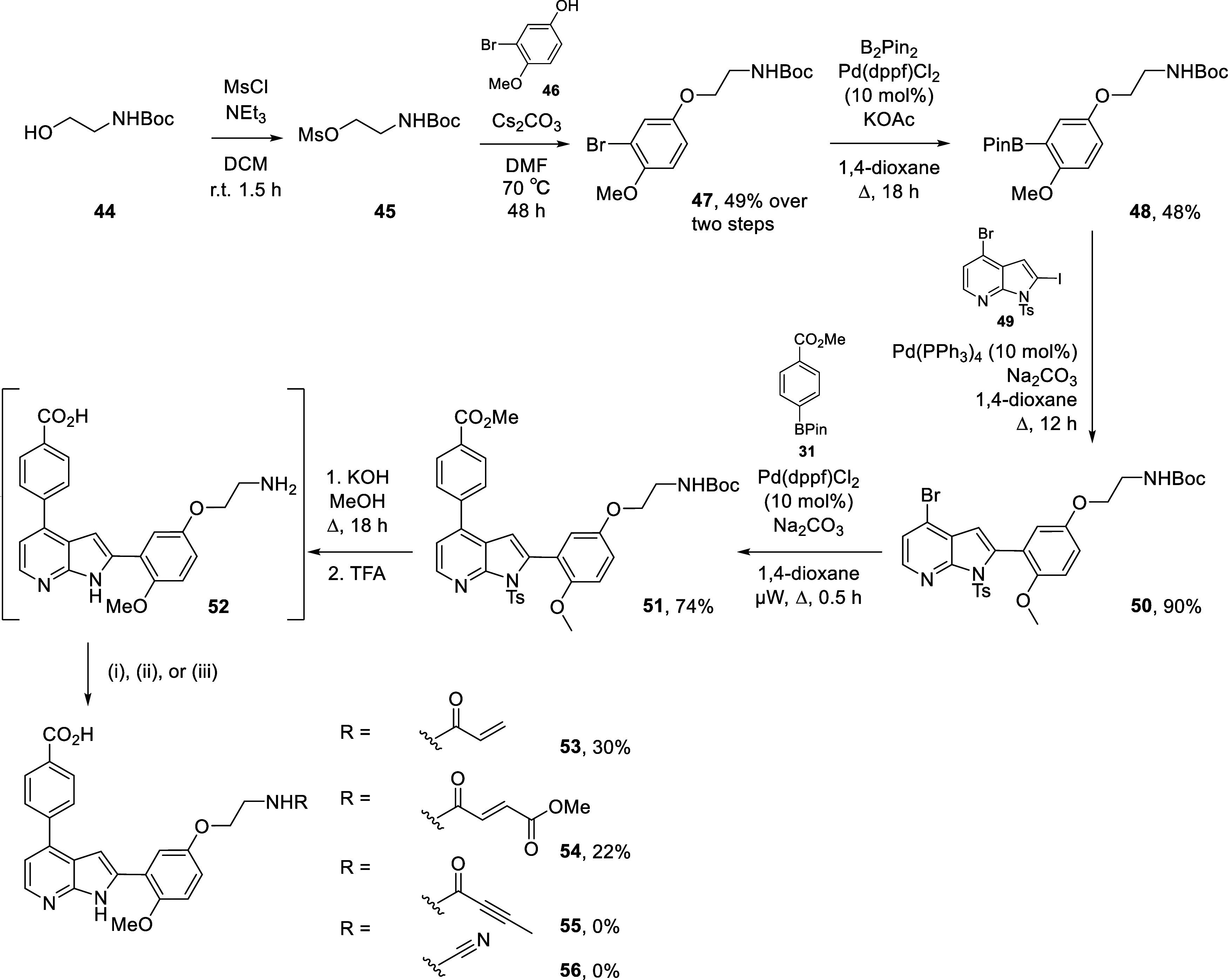
Synthesis of Ethanolamine Linker Analogues **53**–**56** ^a^

a (i) Acryloyl chloride, DIPEA, DMF, r.t., 1 h. (ii) Carboxylic acid, HATU/T3P/SO_2_Cl, DIPEA, DMF, r.t., 18 h. (iii) CNBr, NEt_3_, DMF, r.t., 18 h.

### Potency versus Stability

All analogues were then evaluated using intact protein mass spectrometry, and for their activity against both recombinant *Pf*CLK3 and 3D7 *Pf* parasites (Figure 4). Biochemical potency is given at two different concentrations of ATP, 5 μM (measured Km for *Pf*CLK3) and 3 mM (approximate concentration in parasites), as is outlined in our previous work.^2,15^ As in our previous studies, it was observed that compounds capable of covalent bond formation maintained their high potency when [ATP] = 3 mM, whereas those unable to cross-link lost significant potency. This is consistent with an irreversible preassociation of covalent ligands in the ATP-binding site during a 15 min preincubation employed in our assay. Those ligands which showed partial covalency exhibited intermediary potencies between 100 and 1000 nM.

**Figure 4:**
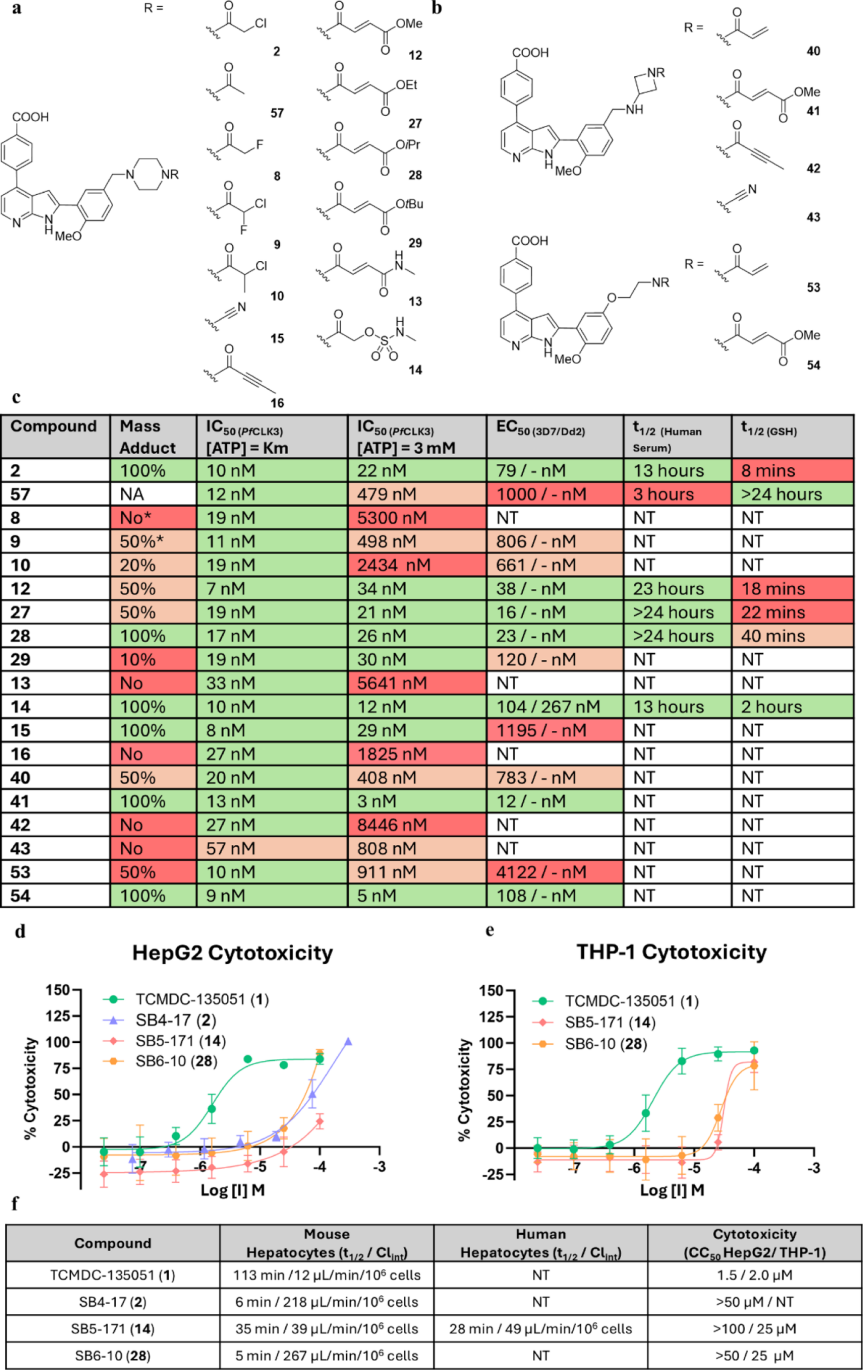
Biological evaluation of novel compounds. (a) Structures of α-reactive library. (b) Structures of variable linker library. (c) Mass spectrometry, enzymatic,* parasiticidal and stability data for all novel compounds, with chloroacetamide SB4–17 (**2**) and noncovalent control **57** for comparison. (d,e) HepG2 and THP-1 cytotoxicity concentration response curves of compounds **1**, **2**, **14**, and **28**. (f) Hepatocytic stability and cytotoxicity data for compound **1**, **2**, **14**, and **28**. NT or - = not tested. All potency values are the result of pooled data over at least 3 separate experiments. Mass adduct formation observed after 4 h at room temperature. *protein concentration = 25 nM. Biochemical potency for covalent compounds quoted as “apparent IC_50_” due to the time-dependent nature of covalent inhibition.

It should be noted that biochemical potency is determined using a protein concentration of 25 nM, placing a lower limit on determined IC_50_ values. Attempts to lower this concentration resulted in unacceptable signal/noise ratios. We also recognize that covalent inhibition is time dependent, and determination of *k*_inact_ is generally the superior metric with which to rank covalent inhibitors. However, due to the lower IC_50_ limit placed by the protein concentration of the assay, accurate time-dependent potency could not be determined. Thus, IC_50_ values are quoted as an “apparent IC_50_” from our standard 2 hour incubation assay. This represents a limitation in our study, and parasiticidal potency is perhaps a better metric with which to rank this data set.

The results of SB4–17 (chloroacetamide **2**) and noncovalent control **57** from our previous work are given in Figure 4 for comparison. All analogues with a parasite potency of <120 nM were then evaluated for their stability in excess glutathione using a HPLC-based assay.

### α-Reactive Warheads with Significantly Reduced Reactivity Covalently Inhibit *Pf*CLK3

Of the α-haloacetamides, both the chlorofluoroacetamide **9** and the α-methyl chloroacetamide **10** (Figure 4a) demonstrated partial covalent modification of *Pf*CLK3 kinase domain (amino acids 334–699). This resulted in intermediate potencies in the parasite assay. These potencies of 806 nM and 661 nM are greater than noncovalent control **57**, but significantly less potent than highly reactive SB4–17 (**2**). While not potent enough to be taken forward as leads, these data show that by matching the exact geometry of the chloroacetamide, far less reactive warheads can engage *Pf*CLK3 covalently. It was encouraging to see formation of the covalent bond by one of the least reactive designs, chlorofluoroacetamide **9**. . We previously demonstrated that the acrylamide, which is more reactive than the chlorofluoroacetamide warhead, was unable to covalently modify our target. This suggests that covalent modification is not governed by reactivity alone, resonating with the present hypothesis which postulates that reactivity can be significantly lowered provided that the optimal geometry is achieved for the covalent reaction. This is an encouraging result for this present strategy.

Fumarate esters **12**, and **27**–**29** all demonstrated covalent binding with the kinase, though adduct formation with the least reactive *tert*-butyl ester **29** was only a minor product (Figure 4a,c). Consistent with this observation, all esters potently killed 3D7 parasites, with EC_50_ values of 16–38 nM with the exception of *tert*-butyl ester **29** (120 nM). Interestingly, fumaramide **13** was unable to engage *Pf*CLK3 covalently and demonstrated poor parasite potency. It is thought that the substitution of the acrylamide decreased its reactivity such that it could not react with Cys368. Further work may seek to increase this reactivity with the addition of electron withdrawing groups to the fumaramide. In terms of stability, half-lives in the presence of glutathione were shown to be 18–40 min for esters **12**, **27** and **28**, with an upward trend observed proportional to steric bulk. The success of the fumarate esters confirms the essentiality of maintaining α-reactive geometry on this scaffold.

Methyl sulfamate **14** (Figure 4a), though less potent than the fumarates, demonstrated covalent binding and good parasite potency of 104 nM (Figure 4c). This analogue proved more stable than the fumarates, with a glutathione half-life of 2 h. Once again, the α-reactive geometry proved key for covalent inhibition. Cyanamide **15** and butynamide **16**, with different geometries, had parasiticidal potencies in the micromolar range. Though cyanamide **15** was able to covalently modify *Pf*CLK3, this was not reflected in its parasitic potency. These analogues were therefore not progressed.

### Varying Linker Geometry Improves Covalent Binding

Among the variable linkers, azetidine-based compounds exhibited a spectrum of outcomes, including several promising results. Neither butynamide **42** nor cyanamide **43** analogues (Figure 4b) demonstrated covalent binding or significant parasiticidal activity. These compounds are believed to possess an unfavorable geometry for engaging Cys368. Acrylamide **40** showed partial covalent adduct formation after 4 h, and demonstrated intermediary potency against 3D7 parasites (EC_50_ = 783 nM, Figure 4c). The use of the shorter azetidine linker was able to improve covalent binding and potency relative to our previously published acrylamide using the piperazine linker, once again highlighting the success of a geometry-based optimization process.^2^ The methyl fumarate equivalent **41** (Figure 4b) showed 100% adduct formation and was highly potent in the parasite assay, with an EC_50_ of 12 nM. The increased activity relative to **40** may be attributed to a mixture of geometry and reactivity differences.

For the ethanolamine linkers (Figure 4a), both demonstrated covalent adduct formation, although acrylamide **53** exhibited only partial modification. Parasite potency was therefore low for the acrylamide **53**, but much improved for fumarate **54**. This is consistent with the results observed for azetidines **40** and **41**.

Overall, comparing all three linkers employed, covalently binding azetidines **40** and **41** were more potent than their piperazine and ethanolamine equivalents. The ethanolamines were the least potent of this series. This systematic study of the effects of geometry versus reactivity on covalent binding and potency has indicated that the α-reactive warheads linked to the core azaindole scaffold via piperazines and azetidines provide the optimal geometry to engage Cys368.

Excited by these encouraging new leads, the two most potent piperazines, methyl sulfamate SB5–171 (**14)** and isopropyl fumarate SB6–10 (**28**) were advanced to ADME studies. To assess their potential, we measured stability in mouse hepatocytes and evaluated cytotoxicity (Figure 4d,e). Isopropyl fumarate **28** proved highly unstable against mouse hepatocytes, with a half-life of only 5 min and an intrinsic clearance of 267 μL/min/10^6^ cells. The methyl sulfamate **14** however proved much more stable, with a half-life of 35 min and an intrinsic clearance of 39 μL/min/10^6^ cells. The stability of SB5–171 (**14**) against human hepatocytes proved similar. This is a significant improvement from our initial covalent compound SB4–17 (**2**), with a half-life of only 6 min against mouse hepatocytes. Both compounds proved noncytotoxic (CC_50_ > 50 μM) against HepG2 cells as well THP-1 cells. Methyl sulfamate **14** therefore achieves a good balance between potency and stability.

It should also be noted that SB5–171 (**14**), our newly found next generation lead, was profiled against the Dd2*P. falciparum*cell line. This is a chloroquinine resistant *Pf* cell line, and has been used previously to study SB4–17’s (**2**) propensity for resistance (reported in our previous work to be low).^4^ The new lead compound **14** displayed a similar potency to the previously reported value of compound **2** (267 nM versus 240 nM). This result from a drug resistant cell line is encouraging given chloroacetamide **2**’s previously low susceptibility to resistance development. We would expect SB5–171 to therefore perform similarly in drug pressure studies, which are ongoing.

Future work will prioritise advancing this lead compound into *in vivo* malaria models to validate its therapeutic potential under physiological conditions. These studies will be critical for confirming efficacy, optimizing pharmacokinetics, and assessing safety, thereby informing the compound’s progression towards preclinical development. More broadly, this approach exemplifies how rational warhead design and linker optimization can deliver covalent inhibitors with improved selectivity and drug-like properties, offering a strategic framework for next-generation antimalarial discovery. Furthermore, the principles established here may be applicable to the development of covalent therapeutics for other indications such as cancer, where optimization of the pharmacokinetics remains a key roadblock for covalent drugs.

## Discussion and Conclusions

The rapid emergence of*P. falciparum*resistance to frontline therapies underscores the urgent need for antimalarial agents with novel mechanisms of action. *Pf*CLK3 has been validated as a multistage target, and covalent kinase inhibitors (CKIs) offer an attractive strategy for achieving sustained target engagement. However, CKI design remains challenging: most efforts focus on tuning warhead reactivity, often at the expense of target engagement. Our work addresses this limitation by demonstrating that geometric optimization, rather than reactivity alone, can drive effective covalent inhibition.

Previous studies have highlighted the importance of warhead positioning in covalent drug design. Ojida and colleagues demonstrated that dihaloacetamides can achieve tunable reactivity through halogen substitution,^18^ while Cravatt’s group introduced fumarate esters to impart kinetic selectivity via metabolic susceptibility.^29^ More recently, London and co-workers developed sulfamate-based electrophiles as warheads with improved metabolic stability profiles.^32^ While these studies advanced the chemical diversity of covalent warheads, they largely focused on intrinsic reactivity or metabolic tuning. In contrast, our geometry-first approach systematically explores how warhead and linker orientation govern covalent bond formation, revealing that maintaining the α-reactive geometry of our chloroacetamide scaffold enables covalent engagement even with substantially less reactive electrophiles. The methyl sulfamate analogue SB5–171 (**14**) exemplifies this principle, combining potent parasiticidal activity with improved hepatocyte stability, a key barrier to clinical translation. Furthermore, our linker optimization studies underscore the importance of conformational control: azetidine-based linkers provided superior potency relative to piperazine and ethanolamine analogues, reinforcing the role of geometry in CKI design.

Our strategy represents a new approach for covalent drug discovery. By decoupling covalent engagement from high intrinsic reactivity, geometric optimization can deliver inhibitors with improved selectivity, stability, and drug-like properties. While our focus has been on malaria, the principles established here are broadly applicable to other parasitic and infectious disease targets, where durable and selective target engagement remains a key challenge. More generally, integrating geometric considerations into covalent inhibitor design could accelerate the development of next-generation therapeutics across diverse disease areas. While the two pillars of covalent drug design, reversible affinity versus covalent reactivity, are well studied and established, this study introduces a third pillar in the form of reaction geometry, which can greatly influence the former two.

In conclusion, this study establishes a geometry-first paradigm for covalent inhibitor design, demonstrating that precise spatial alignment of warhead and linker can enable selective covalent engagement without relying on highly reactive electrophiles. By systematically varying both warhead chemistry and linker architecture, we identified *Pf*CLK3 inhibitors that combine potent parasiticidal activity with improved metabolic stability, exemplified by the methyl sulfamate analogue SB5–171. These findings challenge the conventional emphasis on intrinsic reactivity and highlight geometry as a critical determinant of covalent drug performance. Beyond malaria, this approach offers a broadly applicable framework for developing covalent therapeutics, and has the potential to deliver safer, more effective medicines across diverse therapeutic areas.

## Experimental Section

### Small-Molecule Synthesis and Characterization

Small molecules mentioned in this study were synthesized, with their purity and identity validated using ^1^H and ^13^C NMR, HPLC and HRMS. All tested compounds are >95% pure by HPLC Analysis. Methods and characterization of newly synthesized small molecules are supplied in the Chemical Synthesis and Characterization Data section of the Supporting Information.

### 4-(2-{2-Methoxy-5-[(piperazin-1-yl)­methyl]­phenyl}-1H-pyrrolo­[2,3-*b*]­pyridin-4-yl)­benzoic acid (**26**)

Intermediate **26** was synthesized using the protocol from our previous work. All characterization was in accordance with this literature.^4^

### 4-[2-(5-{[4-(2-Fluoroacetyl)­piperazin-1-yl]­methyl}-2-methoxyphenyl)-1H-pyrrolo­[2,3-*b*]­pyridin-4-yl]­benzoic acid (**8**)

Compound **26** (16 mg, 0.04 mmol, 1.0 equiv) was dissolved in anhydrous DMF and treated with chloroacetyl chloride (4 μL, 0.06 mmol, 1.5 equiv) and triethylamine (30 μL, 0.24 mmol, 6 equiv) and stirred at room temperature for 2 h. The reaction was then treated with tetrabutylammonium fluoride (TBAF) (2.0 M in THF, 113 μL, 0.22 mmol, 5.0 equiv). After 18 h the crude reaction mixture was then purified by reverse phase flash column chromatography on an Isolera one with a 25 g C18 column (5–95% MeCN 0.1% TFA in H_2_O 0.1%) to yield yellow solid **49** (1.0 mg, 4.5% yield). ^**1**^ **H NMR (400 MHz, DMSO)** δ: 12.01 (s, 1H), 8.35 (d, *J* = 4.9 Hz, 1H), 8.14 (dd, *J* = 8.2, 1.4 Hz, 2H), 7.96 – 7.89 (m, 3H), 7.49 (d, *J* = 8.5 Hz, 1H), 7.32 – 7.24 (m, 2H), 7.08 (s, 1H), 5.16 (dd, *J* = 43.8, 14.6 Hz, 2H), 4.33 (s, 2H), 3.96 (s, 3H), 3.42 (br s, 4H), 3.00 (br s, 4H); ^**19**^ **F NMR (377 MHz, DMSO)** δ: −229.58; **HRMS** *m*/*z*: calcd for C_28_H_27_N_4_O_4_F [M + H]^+^, calcd for 503.2089; found, 503.2103; **Retention Time** (min) 21.62 (5–95% ACN 0.1% TFA in H_2_O 0.1% over 50 min), 96% purity.

### 4-[2-(5-{[4-(2-Chloro-2-fluoroacetyl)­piperazin-1-yl]­methyl}-2-methoxyphenyl)-1H-pyrrolo­[2,3-*b*]­pyridin-4-yl]­benzoic acid (**9**)

To a solution of racemic chlorofluoroacetic acid (60 μL, 0.81 mmol, 18 equiv) and DIPEA (154 μL, 0.90 mmol, 20 equiv) was added T3P (50% v/v solution in EtOAc, 482 μL, 18 equiv) and stirred at 0 °C for 1 h. Compound **26** (20 mg, 0.045 mmol, 1 equiv) was then added and the mixture was heated at 50 °C. After 18 h, conversion to product was determined to be 25%, with conversion to an unknown side product at 20%. The crude reaction mixture was then purified by reverse phase flash column chromatography on an Isolera one with a 25g C18 column (5–95 ACN 0.1% TFA in H_2_O 0.1%) to yield a yellow solid (2.1 mg, 9% yield). ^**1**^ **H NMR (400 MHz, DMSO)** δ: 12.02 (s, 1H), 8.35 (d, *J* = 5.0 Hz, 1H), 8.14 (d, *J* = 8.0 Hz, 2H), 7.93 (d, *J* = 8.4 Hz, 3H), 7.50 (d, *J* = 8.5 Hz, 1H), 7.36 – 7.19 (m, 3H), 7.09 (s, 1H), 4.35 (s, 2H), 3.96 (s, 3H), 3.44 (br s, 4H), 3.26 – 2.99 (br s, 4H); ^**19**^ **F NMR (377 MHz, DMSO)** δ: −145.33. **HRMS** *m*/*z*: calcd for C_28_H_26_N_4_O_4_ClF [M + H]^+^, calcd for 537.1699; found, 537.1697; **Retention Time** (min) 23.20 (5–95% ACN 0.1% TFA in H_2_O 0.1% over 50 min), 95% purity.

### 4-[2-(5-{[4-(2-Chloropropanoyl)­piperazin-1-yl]­methyl}-2-methoxyphenyl)-1H-pyrrolo­[2,3-*b*]­pyridin-4-yl]­benzoic acid (**10**)

Compound **26** (13 mg, 0.0275 mmol, 1.0 equiv) in anhydrous DMF (500 μL, 0.1 M) was treated with NEt_3_ (4 μL, 0.034 mmol, 1.25 equiv) and 2-chloropropionyl chloride (3 μL, 0.034 mmol, 1.25 equiv). After 1 h at room temperature, the reaction was retreated with 2 equivalents (0.055 mmol) of each reagent. A further 5 equiv (0.138 mmol) of each reagent was added 1 h later, and after a subsequent hour, the reaction was determined to have completed by LCMS analysis. The crude reaction mixture was then purified by reverse phase flash column chromatography on an Isolera one using a 30g C18 column (5–70 ACN 0.1% TFA in H_2_O 0.1%) to yield a yellow solid (4.5 mg, 31% yield). ^**1**^ **H NMR (400 MHz, DMSO)** δ: 12.06 (d, *J* = 2.1 Hz, 1H), 10.14 (s, 1H), 8.36 (d, *J* = 5.0 Hz, 1H), 8.15 (d, *J* = 8.4 Hz, 2H), 7.97 – 7.89 (m, 3H), 7.51 (dd, *J* = 8.7, 2.0 Hz, 1H), 7.32 – 7.25 (m, 2H), 7.09 (d, *J* = 2.1 Hz, 1H), 5.08 (s, 1H), 4.36 (s, 2H), 3.96 (s, 3H), 3.55 – 2.95 (m, 6H), 1.52 (d, *J* = 6.4 Hz, 3H**);** ^**13**^ **C NMR (101 MHz, DMSO)** δ: 167.0, 157.3, 149.4, 143.1, 142.6, 139.0, 135.8, 132.7, 131.9, 130.5, 130.0, 128.4, 120.2, 118.0, 114.8, 112.4, 99.3, 82.5, 58.5, 56.0, 50.5, 50.2, 50.1. 42.2, 20.7; **HRMS** *m*/*z*: calcd for C_29_H_29_N_4_O_4_Cl [M + H]+, calcd for 533.1950; found, 533.1957; **Retention Time** (min) 23.22 (5–95% ACN 0.1% TFA in H_2_O 0.1% over 60 min), 99% purity.

### 4-{2-[2-Methoxy-5-({4-[(2*E*)-4-methoxy-4-oxobut-2-enoyl]­piperazin-1-yl}­methyl)­phenyl]-1H-pyrrolo­[2,3-*b*]­pyridin-4-yl}­benzoic acid (**12**)

To a solution of monomethyl fumarate (24 mg, 0.18 mmol, 4 equiv) in DMF (1.5 mL, 0.12 M) was added DIPEA (31 μL, 0.18 mmol, 4 equiv) and HATU (69 mg, 0.18 mmol, 4 equiv). The reaction was stirred at room temperature for 15 min, during which time the yellow solution went golden brown. Compound **26** (20 mg, 0.045 mmol, 1 equiv) was then added, and the reaction stirred for a further hour. The crude reaction mixture was then purified by reverse phase flash column chromatography on an Isolera one using a 30g C18 column (10–60% ACN 0.1% TFA in H_2_O 0.1%) to yield a yellow solid (13.5 mg, 56% yield); ^**1**^ **H NMR (400 MHz, DMSO)** δ: 12.05 (d, *J* = 2.2 Hz, 1H), 10.16 (s, 1H), 8.36 (d, *J* = 5.0 Hz, 1H), 8.15 (d, *J* = 8.4 Hz, 2H), 7.96 – 7.89 (m, 3H), 7.50 (dd, *J* = 8.5, 2.2 Hz, 1H), 7.48 (d, *J* = 15.4, 1H), 7.32 – 7.25 (m, 2H), 7.09 (d, *J* = 2.0 Hz, 1H), 6.61 (d, *J* = 15.4 Hz, 1H), 4.34 (s, 2H), 3.96 (s, 3H), 3.73 (s, 3H), 3.44 (br s, 4H), 3.07 (br s, 4H); ^**13**^ **C NMR (101 MHz, DMSO)** δ: 167.0, 165.4, 162.9, 157.3, 149.4, 143.0, 142.6, 139.1, 135.8, 134.2, 132.7, 131.9, 130.6, 130.2, 130.0, 128.4, 121.4, 120.2, 118.1, 114.8, 112.4, 99.3, 58.5, 54.0, 52.2, 50.7, 50.1. ; **HRMS** *m*/*z*: calcd for C_31_H_30_N_4_O_6_ [M + H]+, calcd for 555.2238; found, 555.2236; **Retention Time** (min) 23.60 (5–95% ACN 0.1% TFA in H_2_O 0.1% over 50 min), 99% purity.

### 4-{2-[5-({4-[(2*E*)-4-Ethoxy-4-oxobut-2-enoyl]­piperazin-1-yl}­methyl)-2-methoxyphenyl]-1H-pyrrolo­[2,3-*b*]­pyridin-4-yl}­benzoic acid (**27**)

To a solution of monoethyl fumarate (20 mg, 0.125 mmol, 5 equiv) in DMF (500 μL, 0.25 M) was added DIPEA (43 μL, 0.25 mmol, 10 equiv) and T3P (50 wt % in EtOAc, 74 μL, 0.125 mmol, 5 equiv). The reaction was stirred at room temperature for 15 min, during which time the yellow solution went golden brown. Compound **26** (11 mg, 0.025 mmol, 1 equiv) was then added, and the reaction stirred for a further hour. The crude reaction mixture was then purified by reverse phase HPLC (20–70% ACN 0.1% TFA in H_2_O 0.1%) to yield a yellow solid (6.7 mg, 37% yield); ^**1**^ **H NMR (400 MHz, DMSO)** δ: 12.02 (s, 1H), 8.35 (d, *J* = 5.0 Hz, 1H), 8.14 (d, *J* = 8.4 Hz, 2H), 7.95 (d, J = 2.2, 1H), 7.93 (d, *J* = 8.4, 2H), 7.51 (dd, *J* = 8.5, 2.2 Hz, 1H), 7.46 (d, *J* = 15.4 Hz, 1H), 7.32 – 7.25 (m, 2H), 7.10 (d, *J* = 1.9 Hz, 1H), 6.69 (d, *J* = 1.1 Hz, 1H), 6.59 (d, *J* = 15.4 Hz, 1H), 4.34 (s, 2H), 4.19 (q, *J* = 7.1 Hz, 2H), 3.96 (s, 3H), 1.24 (t, *J* = 7.1 Hz, 3H); * ^**13**^ **C NMR (101 MHz, DMSO)** δ: 167.1, 165.7, 164.9, 163.0, 157.3, 149.4, 143.1, 142.6, 139.1, 135.9, 134.6, 134.1, 132.7, 131.9, 130.6, 130.0, 128.5, 120.2, 118.1, 114.8, 112.4, 99.3, 61.0, 58.6, 56.0, 14.0; **HRMS** *m*/*z*: calcd for C_32_H_32_N_4_O_6_ [M + H]+, calcd for 569.2395; found, 569.2391; **Retention Time** (min) 24.08 (5–95% ACN 0.1% TFA in H_2_O 0.1% over 50 min), 99% purity *Piperazine peaks occluded by H_2_O signals.

### 4-{2-[5-({4-[(2*E*)-4-*iso*-Propoxy-4-oxobut-2-enoyl]­piperazin-1-yl}­methyl)-2-methoxyphenyl]-1H-pyrrolo­[2,3-*b*]­pyridin-4-yl}­benzoic acid (**28**)

To a solution of monoispropyl fumarate (22 mg, 0.138 mmol, 5 equiv) in DMF (550 μL, 0.5 M) was added DIPEA (47 μL, 0.275 mmol, 10 equiv) and T3P (50 wt % in EtOAc, 81 μL, 0.138 mmol, 5 equiv). The reaction was stirred at room temperature for 30 min, during which time the yellow solution went golden brown. Compound **26** (13 mg, 0.0275 mmol, 1 equiv) was then added, and the reaction stirred for a further hour. The crude reaction mixture was then purified by reverse phase HPLC (25–75% ACN 0.1% TFA in H_2_O 0.1%) to yield a yellow solid (6.4 mg, 42% yield); ^**1**^ **H NMR (400 MHz, DMSO)** δ: 12.03 (s, 1H), 8.36 (d, *J* = 4.7 Hz, 1H), 8.15 (d, *J* = 8.4, 2H), 7.97 – 7.89 (m, 3H), 7.51 (dd, *J* = 8.5, 2.2 Hz, 1H), 7.43 (d, *J* = 15.4 Hz, 1H), 7.32 – 7.25 (m, 2H), 7.09 (d, *J* = 1.9 Hz, 1H), 6.56 (d, *J* = 15.4 Hz, 1H), 5.00 (hept, *J* = 6.3 Hz, 1H), 4.34 (s, 3H), 3.96 (s, 3H), 3.47 (br s, 4H), 3.10 (br s, 4H), 1.24 (d, *J* = 6.3 Hz, 6H); ^**13**^ **C NMR (101 MHz, DMSO)** δ: 167.0, 164.4, 163.0, 157.3, 146.9, 143.1, 142.6, 139.0, 135.8, 133.9, 132.6, 131.9, 130.9, 130.5, 130.0, 128.4, 121.3, 120.2, 118.0, 114.8, 112.4, 99.3, 68.4, 58.5, 56.0, 50.6, 50.1, 21.5; **HRMS** *m*/*z*: calcd for C_33_H_34_N_4_O_6_ [M + H]^+^, calcd for 583.2551; found, 583.2552; **Retention Time** (min) 25.37 (5–95% ACN 0.1% TFA in H_2_O 0.1% over 50 min), 100% purity.

### 4-{2-[5-({4-[(2*E*)-4-*tert*-Butoxy-4-oxobut-2-enoyl]­piperazin-1-yl}­methyl)-2-methoxyphenyl]-1H-pyrrolo­[2,3-*b*]­pyridin-4-yl}­benzoic acid (**29**)

To a solution of mono-*tert*-butyl fumarate (23 mg, 0.134 mmol, 5 equiv) in DMF (1.07 mL, 0.125 M) was added DIPEA (45 μL, 0.267 mmol, 10 equiv) and HATU (51 mg, 0.134 mmol, 5 equiv). The reaction was stirred at room temperature for 30 min, during which time the yellow solution went golden brown. Compound **26** (12 mg, 0.0267 mmol, 1 equiv) was then added, and the reaction stirred for a further hour. The crude reaction mixture was then purified by reverse phase HPLC (20–60% ACN 0.1% TFA in H_2_O 0.1%) to yield a yellow solid (3.1 mg, 19% yield); ^**1**^ **H NMR (400 MHz, DMSO)** δ: 12.01 (d, *J* = 2.1 Hz, 1H), 10.00 (s, 1H), 8.35 (d, *J* = 5.0 Hz, 1H), 8.14 (d, *J* = 8.4, 2H), 7.92 (m, 3H), 7.50 (dd, *J* = 8.5, 2.2 Hz, 1H), 7.36 (d, *J* = 15.4 Hz, 1H), 7.31 – 7.25 (m, 2H), 7.08 (d, *J* = 2.1 Hz, 1H), 6.50 (d, *J* = 15.4 Hz, 1H), 4.34 (s, 2H), 3.96 (s, 3H), 1.46 (s, 9H); * ^**13**^ **C NMR (101 MHz, DMSO)** δ: 167.0, 164.1, 163.1, 158.2, 157.3, 149.5, 143.2, 142.6, 139.0, 135.8, 133.2, 132.6, 132.2, 131.8, 130.5, 130.0, 128.4, 120.3, 118.0, 114.8, 112.4, 99.3, 81.2, 58.6, 56.0, 27.6; * **HRMS** *m*/*z*: calcd for C_34_H_36_N_4_O_6_ [M + H]+, calcd for 597.2708; found, 597.2717; **Retention Time** (min) 26.76 (5–95% ACN 0.1% TFA in H_2_O 0.1% over 50 min), 99% purity. *Piperazine peaks not visible due to amide rotamer broadening.

### 4-{2-[2-Methoxy-5-({4-[(2*E*)-3-(methylcarbamoyl)­prop-2-enoyl]­piperazin-1-yl}­methyl)­phenyl]-1H-pyrrolo­[2,3-*b*]­pyridin-4-yl}­benzoic acid (**13**)

To a solution of (E)-4-(methylamino)-4-oxobut-2-enoic acid (17 mg, 0.134 mmol, 5 equiv) in DMF (1.07 mL, 0.125 M) was added DIPEA (45 μL, 0.267 mmol, 10 equiv) and HATU (46 mg, 0.121 mmol, 4.5 equiv). The reaction was stirred at room temperature for 30 min, during which time the colorless solution became deep purple. Compound **26** (12 mg, 0.0267 mmol, 1 equiv) was then added, and the reaction stirred for a further 18 h. The conversion was determined to be 50% by LCMS, and so the reaction was resubjected to the conditions stated above. After a further 18 h, the crude reaction mixture was then purified by reverse phase flash column chromatography on an Isolera one using a 30g C18 column (5–95% ACN 0.1% TFA in H_2_O 0.1%) to yield a yellow solid (10.3 mg, 58% yield); ^**1**^ **H NMR (400 MHz, DMSO)** δ: 12.06 (d, *J* = 2.1 Hz, 1H), 10.15 (s, 1H), 8.43 (q, *J* = 4.7 Hz, 1H), 8.36 (d, *J* = 5.0 Hz, 1H), 8.15 (d, *J* = 8.4 Hz, 2H), 7.96 – 7.89 (m, 3H), 7.50 (dd, *J* = 8.6, 2.1 Hz, 1H), 7.32 – 7.23 (m, 3H), 7.09 (d, *J* = 2.1 Hz, 1H), 6.85 (d, *J* = 15.0 Hz, 1H), 4.34 (s, 2H), 3.95 (s, 3H), 3.43 (s, 4H), 3.04 (s, 4H), 2.69 (d, *J* = 4.7 Hz, 3H); ^**13**^ **C NMR (101 MHz, DMSO)** δ: 167.0, 164.0, 163.6, 157.3, 149.4, 143.1, 142.6, 139.1, 135.9, 135.5, 132.6, 131.9, 130.6, 130.1, 128.5, 127.9, 121.4, 120.2, 118.1, 114.8, 112.4, 99.3, 58.5, 56.0, 25.8; * **HRMS** *m*/*z*: calcd for C_31_H_31_N_5_O_5_ [M + H]+, calcd for 554.2398; found, 554.2400; **Retention Time** (min) 21.84 (5–95% ACN 0.1% TFA in H_2_O 0.1% over 50 min), 95% purity. *Piperazine peaks not visible due to amide rotamer broadening.

### 4-(2-{5-[(4-{2-[(*tert*-Butyldiphenylsilyl)­oxy]­acetyl}­piperazin-1-yl)­methyl]-2-methoxyphenyl}-1H-pyrrolo­[2,3-*b*]­pyridin-4-yl)­benzoic acid (**S1**)

To a solution of 2-((t-butyldiphenylsilyl)­oxy)­acetic acid (173 mg, 0.55 mmol, 5.0 equiv) in DMF (1.0 mL, 0.5 M) was added DIPEA (190 μL, 1.1 mmol, 10.0 equiv) and HATU (168 mg, 0.44 mmol, 4.0 equiv). The mixture was stirred at room temperature for 30 min before compound **26** (50 mg, 0.11 mmol, 1.0 equiv) was added, and the reaction was stirred for a further 18 h at room temperature. The crude reaction mixture was then purified by reverse phase flash column chromatography on an Isolera one using a 30g C18 column (5–95 ACN 0.1% TFA in H_2_O 0.1%) to yield a yellow solid (43 mg, 81% yield). ^**1**^ **H NMR (400 MHz, DMSO)** δ: 12.06 – 12.01 (m, 1H), 10.16 (s, 1H), 8.36 (d, *J* = 5.0 Hz, 1H), 8.15 (d, *J* = 8.6 Hz, 2H), 7.97 – 7.87 (m, 3H), 7.66 – 7.59 (m, 4H), 7.51 – 7.35 (m, 7H), 7.28 (m, 2H), 7.09 (d, *J* = 2.0 Hz, 1H), 4.42 (s, 2H), 4.33 (s, 2H), 3.95 (s, 3H), 3.52 – 3.25 (br s, 4H), 2.96 (br s, 4H), 0.97 (s, 9H); ^**13**^ **C NMR (101 MHz, DMSO)** δ: 168.1, 167.0, 157.3, 149.4, 143.1, 142.6, 139.0, 135.8, 135.1, 134.5, 132.6, 130.5, 130.0, 128.4, 127.9, 127.5, 121.2, 120.2, 118.0, 114.8, 112.4, 99.3, 62.8, 58.5, 56.0, 26.5, 18.9; **HRMS** *m*/*z*: calcd for C_44_H_46_N_4_O_5_Si [M-H]- calc for 737.3165 found 737.3157.

### 4-(2-{2-Methoxy-5-[(4-{2-[(methylsulfamoyl)­oxy]­acetyl}­piperazin-1-yl)­methyl]­phenyl}-1H-pyrrolo­[2,3-*b*]­pyridin-4-yl)­benzoic acid (**14**)

To a solution of **S1** (15 mg, 0.02 mmol, 1 equiv) in DMF (676 μL, 0.03 M) was added CsF (31 mg, 0.2 mmol, 10.0 equiv) and the reaction was heated at 60 °C for 6 h under inert atmosphere. After cooling to room temperature, the crude reaction mixture was then concentrated in vacuo, redissolved in anhydrous DMF (100 μL, 0.2 M) and treated with DIPEA (84 μL, 0.48 mmol, 24.0 equiv) and methyl sulfamoyl chloride (126 mmol, 1.44 mmol, 72.0 equiv). The reaction was stirred at room temperature for 1 h. The crude reaction mixture was then quenched with H_2_O (100 μL) and purified by reverse phase flash column chromatography on an Isolera one using a 30 g C18 column (5–95 MeCN 0.1% TFA in H_2_O 0.1%). The crude reaction mixture was then purified by reverse phase flash column chromatography on an Isolera one using a 30 g C18 column (5–95 MeCN 0.1% TFA in H_2_O 0.1%) to yield yellow solid (5.6 mg, 47% yield, 38% yield from compound **26**). ^**1**^ **H NMR (400 MHz, DMSO)** δ: 12.03 (s, 1H), 8.36 (d, *J* = 5.0 Hz, 1H), 8.14 (d, *J* = 8.2 Hz, 2H), 7.97 – 7.89 (m, 3H), 7.83 (q, *J* = 4.8 Hz, 1H), 7.51 (dd, *J* = 8.6, 2.2 Hz, 1H), 7.32 – 7.25 (m, 2H), 7.09 (d, *J* = 2.0 Hz, 1H), 4.81 (s, 2H), 4.35 (s, 2H), 3.96 (s, 3H), 3.42 (br s, 4H), 3.01 (br s, 4H), 2.60 (d, *J* = 4.8 Hz, 3H); ^**13**^ **C NMR (101 MHz, DMSO)** δ: 167.0, 164.1, 157.3, 149.5, 142.6, 139.0, 135.8, 132.7, 130.5, 130.0, 128.4, 120.3, 118.0, 114.8, 112.4, 107.2, 101.1, 99.3, 88.5, 75.0, 65.6, 56.0, 50.1, 28.9; **HRMS** *m*/*z*: calcd for C_29_H_31_N_5_O_7_S [M-H]- calc for 592.1871 found 592.1874; **Retention Time** (min) 23.62 (5–95% ACN 0.1% TFA in H_2_O 0.1% over 50 min), 97% purity.

### 4-(2-{5-[(4-Cyanopiperazin-1-yl)­methyl]-2-methoxyphenyl}-1H-pyrrolo­[2,3-*b*]­pyridin-4-yl)­benzoic acid (**15**)

Compound **26** (13 mg, 0.0275 mmol, 1.0 equiv) in DMF (500 μL, 0.1 M) and aqueous sodium hydrogen carbonate (36 μL) was treated with cyanogen bromide (3.2 mg, 0.025 mmol, 0.9 equiv) and stirred at room temperature for 18 h, during which time the yellow reaction mixture turned vivid orange. The crude reaction mixture was then purified by reverse phase flash column chromatography on an Isolera one using a 30g C18 column (10–60% ACN 0.1% TFA in H_2_O 0.1%) to yield a yellow solid (8.15 mg, 66% yield); ^**1**^ **H NMR (400 MHz, DMSO)** δ: 12.06 (s, 1H), 8.36 (d, *J* = 5.0 Hz, 1H), 8.14 (d, *J* = 8.4 Hz, 2H), 7.97 – 7.89 (m, 3H), 7.50 (dd, *J* = 8.5, 2.2 Hz, 1H), 7.32 – 7.24 (m, 2H), 7.10 (d, *J* = 1.7 Hz, 1H), 4.35 (s, 2H), 3.95 (s, 3H), 3.29 (br s, 4H); * ^**13**^ **C NMR (101 MHz, DMSO)** δ: 167.2, 157.4, 149.2, 143.0, 142.6, 139.4, 136.1, 132.9, 132.0, 130.7, 130.2, 128.6, 121.4, 120.3, 118.3, 116.4, 115.0, 112.5, 99.4, 58.8, 56.1, 49.5, 45.6; **HRMS** *m*/*z*: calcd for C_27_H_25_N_5_O_3_ [M + H]+, calcd for 468.2030; found, 468.2027; **Retention Time** (min) 22.17 (5–95% ACN 0.1% TFA in H_2_O 0.1% over 50 min), 99% purity. *One piperazine peak occluded by H_2_O signal.

### 4-[2-(5-{[4-(But-2-ynoyl)­piperazin-1-yl]­methyl}-2-methoxyphenyl)-1H-pyrrolo­[2,3-*b*]­pyridin-4-yl]­benzoic acid (**16**)

A solution of 2-butynoic acid (22 mg, 0.27 mmol, 5.0 equiv), DIPEA (94 μL, 0.54 mmol, 10.0 equiv) and T3P (50% v/v solution in EtOAc, 163 μL, 0.27 mmol, 5.0 equiv) was stirred in DMF (1.08 mL, 0.25 M) at room temperature for 30 min before compound **26** (24 mg, 0.054 mmol, 1 equiv) was added. After 1 h, the crude reaction mixture was then purified by reverse phase flash column chromatography on an Isolera one using a 30g C18 column (5–70% ACN 0.1% TFA in H_2_O 0.1%) to yield a yellow solid (9.3 mg, 34% yield); ^**1**^ **H NMR (400 MHz, DMSO**) δ: 12.10 (d, *J* = 2.0 Hz, 1H), 10.21 (s, 1H), 8.37 (d, *J* = 5.0 Hz, 1H), 8.15 (d, *J* = 5.0 Hz, 2H), 7.98 – 7.89 (m, 3H), 7.51 (dd, *J* = 8.6, 2.1 Hz, 1H), 7.32 – 7.25 (m, 2H), 7.10 (d, *J* = 2.0 Hz, 1H), 4.34 (s, 2H), 3.96 (s, 3H), 3.44 (br s, 4H), 3.06 (br s, 4H), 2.03 (s, 3H); ^**13**^ **C NMR (101 MHz, DMSO)** δ: 167.0, 157.3, 152.0, 149.2, 142.9, 142.6, 139.2, 135.9, 132.7, 131.9, 130.6, 130.1, 128.5, 121.4, 120.2, 118.2, 114.8, 112.4, 99.4, 90.6, 72.2, 58.5, 56.0, 50.5, 50.0, 3.4; **HRMS** *m*/*z*: calcd for C_30_H_28_N_4_O_4_ [M+2H]^2+^ calc for 255.1128 found 255.1124; **Retention Time** (min) 22.90 (5–95% ACN 0.1% TFA in H_2_O 0.1% over 50 min), 99% purity.

### 3-[4-Bromo-1-tosyl-1H-pyrrolo­[2,3-*b*]­pyridin-2-yl]-4-methoxy-benzaldehyde (**30**)

Intermediate **30** was synthesized using the protocol from our previous work. All characterization was in accordance with this literature.^4,19^

### Methyl-4-[2-(5-formyl-2-methoxyphenyl)-1-(4-methylbenzenesulfonyl)-1H-pyrrolo­[2,3*b*]­pyridin-4-yl]­benzoate (**32**)

To a 35 mL microwave vial containing **30** (2.3 g, 4.8 mmol, 1.0 equiv), methyl 4-(4,4,5,5-tetramethyl-1,3,2-dioxaborolan-2-yl)­benzoate **31**, (1.4 g, 5.3 mmol, 1.1 equiv) and Pd­(dppf)­Cl_2_·CH_2_Cl_2_ complex (0.2 g, 0.2 mmol, 0.05 equiv) in 1,4-dioxane under nitrogen was added 2 M aqueous sodium carbonate (12.0 mL, 23.9 mmol, 5.0 equiv). The reaction mixture was purged with nitrogen for 10 min and microwaved at 110 °C for 45 min. The reaction mixture was diluted with 1:1 water/ethyl acetate and two layers were separated. The aqueous layer was extracted with ethyl acetate and the combined organic layers were dried over magnesium sulfate and concentrated in vacuo to give a brown solid. This was purified using automated flash column chromatography eluting with 25–50% ethyl acetate/petroleum ether. The desired fractions were combined and concentrated in vacuo to give compound **32** as a light brown solid (0.7 g, 29%). R_f_: 0.5 (50% EtOAc in petroleum ether); ^1^H NMR (400 MHz, CDCl_3_) δ: 9.96 (s, 1H), 8.50 (d, *J* = 5.0 Hz, 1H), 8.14 (dt, *J* = 8.4, 1.8 Hz, 2H), 8.00 (dd, *J* = 8.5, 2.1 Hz, 1H), 7.91 (d, *J* = 2.1 Hz, 1H), 7.87 (dt, *J* = 8.2, 1.8 Hz, 2H), 7.68 (dt, *J* = 8.4, 1.8 Hz, 2H), 7.27 (d, *J* = 5.0 Hz, 1H), 7.22 (d, *J* = 8.2 Hz, 2 x CH, 2H), 7.11 (d, *J* = 8.5 Hz, 1H), 6.70 (s, 1H), 3.94 (s, 3H), 3.93 (s, 3H), 2.36 (s, 3H).^**13**^C NMR (101 MHz, CDCl_3_) δ: 190.5, 166.6, 163.5, 149.7, 145.0, 144.9, 142.0, 141.2, 137.6, 136.0, 134.3, 131.2, 130.3, 130.2, 129.3, 129.3, 128.5, 128.1, 123.5, 119.7, 118.2, 110.5, 107.4, 56.1, 52.3, 21.6. **HRMS** *m/z*: calcd for C_30_H_24_N_2_O_6_S (M + H)^+^ 541.1421 found 541.1423.

### *tert*-butyl 3-[(4-Methoxy-3-{4-[4-(methoxycarbonyl)­phenyl]-1-(4-methylbenzenesulfonyl)-1H-pyrrolo­[2,3-*b*]­pyridin-2-yl}­phenyl)­methyl]-1,3-diazinane-1-carboxylate (**33**)

To a solution of **32** (0.05 g, 0.09 mmol, 1.0 equiv) in anhydrous dichloroethane (3 mL) was added tert-butyl-1,3-diazinane-1-carboxylate (0.05 g, 0.28 mmol, 3.0 equiv) and titanium isopropoxide (0.05 mL, 0.19 mmol, 2.0 equiv) and the solution was stirred under argon at room temperature for 10 min. Sodium triacetoxyborohydride (0.05 mg, 0.23 mmol, 2.5 equiv) was added and the solution was stirred under argon at room temperature for 3 h. Sodium triacetoxyborohydride (0.02 mg, 0.09 mmol, 1.0 equiv) was added and the solution was stirred under argon at room temperature for 18 h. The solution was then quenched by the addition of ammonium hydroxide solution (5 mL) and the reaction mixture was diluted with 1:1 water/dichloromethane and two layers were separated. The aqueous layer was extracted with dichloromethane, and the combined organic layers were washed with brine, dried over magnesium sulfate, and concentrated in vacuo to give a colorless oil. This oil was purified using automated flash column chromatography eluting with 45–80% ethyl acetate/petroleum ether. The desired fractions were combined and concentrated in vacuo to give compound **33** as a colorless oil (0.04 g, 65%). R_f_: 0.48 (60% EtOAc in petroleum ether). ^1^H NMR (400 MHz, CDCl_3_) δ: 8.48 (d, *J* = 5.1 Hz, 1H), 8.13 (dt, *J* = 8.4, 2.0 Hz, 2H), 7.91 (dt, *J* = 8.2, 2.9 Hz, 2H), 7.68 (dt, *J* = 8.4, 2.0 Hz, 2H), 7.40 (dd, *J* = 8.4, 2.2 Hz, 1H), 7.32 (d, *J* = 2.2 Hz, 1H), 7.25 (d, *J* = 5.1 Hz, 1H), 7.20 (d, *J* = 8.2 Hz, 2H), 6.92 (d, *J* = 8.4 Hz, 1H), 6.66 (s, 1H), 4.20 (br s, 1H), 4.12 (br s, 1H), 3.94 (s, 3H), 3.78 (s, 3H), 3.63 (s, 2H), 3.51 (m, 2H), 2.75 (s, 2H), 2.35 (s, 3H), 1.65 (s, 2H), 1.41 (s, 9H). ^13^C NMR (101 MHz, CDCl_3_) δ: 166.7, 157.6, 154.5, 149.8, 144.5, 142.3, 140.8, 139.3, 136.4, 131.7, 131.4, 130.2, 129.2, 128.5, 128.2, 122.0, 119.9, 118.0, 110.2, 106.8, 79.7, 77.3, 55.6, 52.3, 51.3, 28.4, 21.6. **HRMS** *m/z*: calcd for C_39_H_42_N_4_O_7_S (M + H)^+^ 711.2840 found 711.2839.

### *tert*-Butyl 3-[(4-methoxy-3-{4-[4-(methoxycarbonyl)­phenyl]-1-(4-methylbenzenesulfonyl)-1H-pyrrolo­[2,3-*b*]­pyridin-2-yl}­phenyl)­methyl]­imidazolidine-1-carboxylate (**34**)

To a solution of **32** (0.05 g, 0.09 mmol, 1.0 equiv) in anhydrous dichloroethane (3 mL) was added *tert*-butyl imidazolidine-1-carboxylate (0.05 g, 0.28 mmol, 3.0 equiv) and the solution was stirred under argon at room temperature for 10 min. Sodium triacetoxyborohydride (0.05 mg, 0.23 mmol, 2.5 equiv) was added and the solution was stirred under argon at room temperature for 3 h. Sodium triacetoxyborohydride (0.02 mg, 0.09 mmol, 1.0 equiv) was added and the solution was stirred under argon at room temperature for 18 h. The solution was then quenched by the addition of ammonium hydroxide solution (5 mL) and the reaction mixture was diluted with 1:1 water/dichloromethane and two layers were separated. The aqueous layer was extracted with dichloromethane, and the combined organic layers were washed with brine, dried over magnesium sulfate and concentrated in vacuo to give a colorless oil. This oil was purified using automated flash column chromatography eluting with 45–80% ethyl acetate/petroleum ether. The desired fractions were combined and concentrated in vacuo to give compound **34** as a colorless oil (0.06 mg, 89%). R_f_: 0.21 (45% EtOAc in petroleum ether). ^1^H NMR (400 MHz, CDCl_3_) δ: 8.49 (d, *J* = 5.1 Hz, 1H), 8.13 (d, *J* = 8.1 Hz, 2H), 7.91 (dt, *J* = 8.2, 2.0 Hz, 2H), 7.68 (d, *J* = 8.1 Hz, 2H), 7.40 (d, *J* = 7.8 Hz, 1H), 7.34 (d, *J* = 4.0 Hz, 1H), 7.25 (d, *J* = 5.1 Hz, 1H), 7.21 (d, *J* = 8.2 Hz, 2H), 6.94 (d, *J* = 7.8 Hz, 1H), 6.66 (s, 1H), 4.04 (d, *J* = 21.4 Hz, 2H), 3.94 (s, 3H), 3.79 (s, 3H), 3.67 (s, 2H), 3.47 (dt, *J* = 21.4, 7.0 Hz, 2H), 2.88 (t, *J* = 7.0 Hz, 2H), 2.35 (s, 3H), 1.44 (s, 9H). ^13^C NMR (101 MHz, CDCl_3_) δ: 166.6, 157.7, 153.6, 149.8, 144.6, 142.3, 140.9, 139.0, 136.4, 131.5, 131.1, 130.2, 129.2, 128.5, 128.2, 122.1, 119.9, 118.0, 110.3, 106.9, 77.2, 57.4, 55.6, 52.3, 28.5, 21.6. **HRMS** *m/z*: calcd for C_38_H_40_N_4_O_7_S (M + H)^+^ 697.2675 found 697.2678.

### *tert*-butyl 2-[(4-Methoxy-3-{4-[4-(methoxycarbonyl)­phenyl]-1-(4-methylbenzenesulfonyl)-1H-pyrrolo­[2,3-*b*]­pyridin-2-yl}­phenyl)­methyl]­pyrazolidine-1-carboxylate (**35**)

To a solution of **32** (0.1 g, 0.19 mmol, 1.0 equiv) in anhydrous dichloroethane (5 mL) was added *tert*-butyl pyrazolidine-1-carboxylate (0.1 g, 0.56 mmol, 3.0 equiv) and magnesium sulfate (0.1 g, 0.93 mmol, 5.0 equiv) and the solution was stirred under argon at room temperature for 15 min. Sodium triacetoxyborohydride (0.1 g, 0.58 mmol, 2.5 equiv) was added and the solution was stirred under argon at room temperature for 3 h. Sodium triacetoxyborohydride (0.04 g, 0.19 mmol, 1.0 equiv) was added and the solution was stirred under argon at room temperature for 18 h. The solution was then quenched by the addition of ammonium hydroxide solution (5 mL) and the reaction mixture was diluted with 1:1 water/dichloromethane and two layers were separated. The aqueous layer was extracted with dichloromethane, and the combined organic layers were washed with brine, dried over magnesium sulfate, and concentrated in vacuo to give a colorless oil. This oil was purified using automated flash column chromatography eluting with 40–70% ethyl acetate/petroleum ether. The desired fractions were combined and concentrated in vacuo to give compound **35** as a colorless oil (0.1 g, 80%). R_f_: 0.33 (40% EtOAc in petroleum ether); ^1^H NMR (400 MHz, CDCl_3_) δ: 8.48 (d, *J* = 5.1 Hz, 1H), 8.13 (dt, *J* = 8.1 Hz, 2H), 7.91 (dt, *J* = 8.2, 2.0 Hz, 2H), 7.68 (d, *J* = 8.1 Hz, 2H), 7.46 – 7.43 (m, 1H), 7.42 – 7.40 (m, 1H), 7.24 (d, *J* = 5.1 Hz, 1H), 7.21 (d, *J* = 8.2 Hz, 2H), 6.92 (d, *J* = 7.8 Hz, 1H), 6.67 (s, 1H), 3.94 (s, 3H), 3.86 – 3.80 (m, 2H), 3.79 (s, 3H), 3.59 – 3.47 (m, 2H), 3.01 (t, *J* = 7.1 Hz, 2H), 2.35 (s, 3H), 2.07 (pent, *J* = 7.2 Hz, 2H), 1.47 (s, H-48, 9H). ^13^C NMR (101 MHz, CDCl_3_) δ: 166.7, 157.8, 149.7, 144.5, 144.5, 142.3, 140.8, 139.1, 136.4, 132.3, 132.0, 130.1, 129.2, 128.5, 128.2, 122.0, 119.9, 118.0, 110.2, 106.9, 80.0, 77.2, 60.0, 55.6, 52.3, 28.5, 23.9, 21.6; **HRMS** *m/z*: calcd for C_38_H_40_N_4_O_7_S (M + H)^+^ 697.2684 found 697.2685.

### *tert*-Butyl 3-{[(4-methoxy-3-{4-[4-(methoxycarbonyl)­phenyl]-1-(4-methylbenzenesulfonyl)-1H-pyrrolo­[2,3-*b*]­pyridin-2-yl}­phenyl)­methyl]­amino}­azetidine-1-carboxylate (**36**)

To a solution of compound **32** (21 mg, 0.04 mmol, 1.0 equiv) in dichloroethane (0.4 mL, 0.1 M) was added 1-Boc-3-(amino)­azetidine (22 mg, 0.12 mmol, 3.0 equiv), Titanium isopropoxide (24 μL, 0.08 mmol, 2.0 equiv) and sodium triacetoxyborohydride (21 mg, 0.1 mmol, 2.5 equiv). The solution was allowed to stir for 3 h at room temperature, before another equivalent of sodium triacetoxyborohydride (8 mg, 0.04 mmol) was added, and stirred for 18 h. Ammonium hydroxide and water were then added, and the solution partitioned between the water and CH_2_Cl_2_. The aqueous layer was extracted with CH_2_Cl_2_ three times, and the organic layers dried over magnesium sulfate.

The filtrate was concentrated in vacuo and purified by automated flash column chromatography on an Isolera one (0–10% MeOH in CH_2_Cl_2_) yielding colorless oil **36** (18 mg, 67% yield); ^1^H NMR (400 MHz, CD_3_OD) δ: 8.35 (d, *J* = 5.1 Hz, 1H), 8.10 (d, *J* = 8.5 Hz, 2H), 7.81 (d, *J* = 8.4 Hz, 2H), 7.69 (d, *J* = 8.5 Hz, 2H), 7.65 – 7.58 (m, 2H), 7.32 (d, *J* = 5.1 Hz, 1H), 7.29 (d, *J* = 8.4 Hz, 2H), 7.16 (d, *J* = 8.4 Hz, 1H), 6.71 (s, 1H), 4.28 (s, 2H), 4.26 – 4.14 (m, 3H), 4.00 (s, 2H), 3.91 (s, 3H), 3.79 (s, 3H), 2.33 (s, 3H), 1.40 (s, 9H); ^13^C NMR (101 MHz, CD_3_OD) δ: 160.8, 157.6, 150.9, 146.8, 145.7, 143.1, 142.7, 139.7, 137.2, 133.8, 133.1, 131.6, 131.2, 130.5, 129.9, 129.8, 129.0, 124.3, 123.3, 121.1, 119.6, 112.4, 108.6, 81.9, 56.2, 52.8, 50.1, 47.5, 28.5, 21.5; * **HRMS** *m/z*: calcd for C_38_H_40_N_4_O_7_S [M + H]+, 697.2690; found, 697.2704. *Azetidine CH_2_s not visible due to amide rotamer broadening.

### 4-[2-(5-{[(Azetidin-3-yl)­amino]­methyl}-2-methoxyphenyl)-1H-pyrrolo­[2,3-*b*]­pyridin-4-yl]­benzoic acid (S2)

Compound **36** (22 mg, 0.035 mmol, 1.0 equiv) was dissolved in MeOH (0.7 mL, 0.05 M) and treated with KOH (18 mg, 0.35 mmol, 10.0 equiv) and heated to reflux for 18 h. The solution was cooled to room temperature and concentrated in vacuo, before being treated with TFA (1.0 mL) for 1 h. After concentration and coevaporation with MeOH, the resulting residue was taken forward to the next step without further purification.

### 4-{2-[2-Methoxy-5-({[1-(prop-2-enoyl)­azetidin-3-yl]­amino}­methyl)­phenyl]-1H-pyrrolo­[2,3-*b*]­pyridin-4-yl}­benzoic acid (**40**)

To a solution of **S2** (22 mg, 0.03 mmol, 1.0 equiv) was dissolved in anhydrous DMF (632 μL, 0.05 M), and treated with DIPEA (32 μL, 0.18 mmol, 6.0 equiv) and acryloyl chloride (4 μL, 0.05 mmol, 1.5 equiv) and left to stir at room temperature for 1 h. Another batch of acryloyl chloride (2.6 μL, 0.03 mmol, 1.0 equiv) was added and left to stir for a further hour. The reaction was quenched with water and the crude reaction mixture was then purified by reverse phase flash column chromatography on an Isolera one using a 30g C18 column (5–95% ACN 0.1% TFA in H_2_O 0.1%) to yield a yellow solid (3.7 mg, 24% yield); ^**1**^ **H NMR (400 MHz, DMSO)** δ: 11.97 (d, *J* = 2.1 Hz, 1H), 9.56 (s, 1H), 8.35 (d, *J* = 5.0 Hz, 1H), 8.18 – 8.11 (d, *J* = 8.4 Hz, 2H), 7.98 (d, *J* = 2.2 Hz, 1H), 7.93 (d, *J* = 8.4 Hz, 2H), 7.49 (dd, *J* = 8.5, 2.2 Hz, 1H), 7.31 – 7.23 (m, 2H), 7.08 (d, *J* = 2.1 Hz, 1H), 6.33 (dd, *J* = 17.0, 10.3 Hz, 1H), 6.29 (d, *J* = 10.3 Hz, 1H), 6.13 (dd, *J* = 17.0, 2.2 Hz, 1H), 5.72 (dd, *J* = 10.3, 2.2 Hz, 1H), 4.54 – 4.46 (m, 1H), 4.28 – 4.22 (m, 1H), 4.18 – 4.10 (m, 4H), 4.00 (d, *J* = 6.2, 1H), 3.96 (s, 3H), 3.91 (d, *J* = 6.8, 1H); ^**13**^ **C NMR (101 MHz, DMSO)** δ: 167.0, 164.8, 156.9, 149.5, 143.2, 142.6, 138.9, 136.0, 131.3, 130.5, 130.0, 128.4, 127.2, 126.6, 123.5, 120.1, 118.0, 114.8, 112.3, 99.0, 55.9, 53.2, 51.0, 48.5, 47.8, 45.6; **HRMS** *m*/*z*: calcd for C_28_H_26_N_4_O_4_ [M + H]+, calcd for 483.2027; found, 484.2023; **Retention Time** (min) 21.93 (5–95% ACN 0.1% TFA in H_2_O 0.1% over 50 min), 96% purity.

### 4-(2-{2-Methoxy-5-[({1-[(2*E*)-4-methoxy-4-oxobut-2-enoyl]­azetidin-3-yl}­amino)­methyl]­phenyl}-1H-pyrrolo­[2,3-*b*]­pyridin-4-yl)­benzoic acid (**41**)

A solution of monomethyl fumarate (12 mg, 0.1 mmol, 5.0 equiv), DIPEA (33 μL, 0.19 mmol, 10.0 equiv) and propanephosphonic acid anhydride (50% v/v solution in EtOAc, 57 μL, 0.1 mmol, 5.0 equiv) in DMF (380 μL, 0.05 M) was stirred at room temperature for 30 min. **S2** (8 mg, 0.019 mmol, 1.0 equiv) was added and the solution stirred for a further 18 h. The crude mixture was then purified by reverse phase flash column chromatography on an Isolera one using a 30 g C18 column (5–60% MeCN 0.1% TFA in H_2_O 0.1%) to yield a yellow solid (7.2 mg, 93% yield); ^**1**^ **H NMR (400 MHz, DMSO)** δ: 12.04 (d, *J* = 2.1 Hz, 1H), 9.67 (s, 2H), 8.36 (d, *J* = 5.0 Hz, 1H), 8.15 (d, *J* = 8.4 Hz, 2H), 7.99 (d, *J* = 2.2 Hz, 1H), 7.93 (d, *J* = 8.4 Hz, 2H), 7.50 (dd, *J* = 8.6, 2.2 Hz, 1H), 7.29 (d, *J* = 5.0 Hz, 1H), 7.27 (d, *J* = 8.6 Hz, 1H), 7.10 (d, *J* = 2.1 Hz, 1H), 6.99 (d, *J* = 15.5 Hz, 1H), 6.62 (d, *J* = 15.5 Hz, 1H), 4.67 – 4.58 (m, 1H), 4.39 (dd, *J* = 10.6, 4.0 Hz, 1H), 4.26 – 4.09 (m, 4H), 4.05 (dd, *J* = 10.6, 3.6 Hz, 1H), 3.96 (s, 3H), 3.73 (s, 3H); ^**13**^ **C NMR (101 MHz, DMSO)** δ: 167.0, 165.3, 162.9, 156.9, 149.2, 142.8, 142.6, 139.3, 136.2, 132.2, 131.4, 130.7, 130.6, 130.0, 129.5, 128.5, 123.6, 120.0, 118.2, 114.8, 112.4, 99.0, 56.0, 53.5, 52.2, 51.5, 47.8, 45.6; **HRMS** *m*/*z*: calcd for C_30_H_28_N_4_O_6_ [M + H]+, calcd for 541.2082; found, 541.2076; **Retention Time** (min) 22.64 (5–95% ACN 0.1% TFA in H_2_O 0.1% over 50 min), 99% purity. *Azetidine CH_2_ signals nonequivalent.

### 4-{2-[5-({[1-(But-2-ynoyl)­azetidin-3-yl]­amino}­methyl)-2-methoxyphenyl]-1H-pyrrolo­[2,3-*b*]­pyridin-4-yl}­benzoic acid (**42**)

A solution of 2-butynoic acid (8 mg, 0.1 mmol, 5.0 equiv), DIPEA (33 μL, 0.19 mmol, 10.0 equiv) and T3P (50% v/v solution in EtOAc, 57 μL, 0.1 mmol, 5.0 equiv) in DMF (380 μL, 0.05 M) was stirred at room temperature for 30 min. Compound **S2** (0.019 mmol, 1.0 equiv) was added and the solution stirred for a further 18 h. The crude mixture was then purified by reverse phase flash column chromatography on an Isolera one using a 30g C18 column (5–60% ACN 0.1% TFA in H_2_O 0.1%) to yield a yellow solid (7.2 mg, 77% yield); ^**1**^ **H NMR (400 MHz, DMSO)** δ: 12.01 (d, *J* = 2.0 Hz, 1H), 9.61 (m, 2H), 8.36 (d, *J* = 5.0 Hz, 1H), 8.14 (d, *J* = 8.3 Hz, 2H), 7.99 (d, *J* = 2.2 Hz, 1H), 7.93 (d, *J* = 8.3 Hz, 2H), 7.50 (dd, *J* = 8.6, 2.2 Hz, 1H), 7.29 (d, *J* = 5.0 Hz, 1H), 7.26 (d, *J* = 8.6 Hz, 1H), 7.09 (d, *J* = 2.0 Hz, 1H), 4.39 (dd, *J* = 10.4, 6.6 Hz, 1H), 4.25 – 4.08 (m, 5H), 3.96 (m, 4H), 2.01 (s, 3H); ^**13**^ **C NMR (101 MHz, DMSO)** δ: 167.0, 156.9, 153.4, 149.3, 143.0, 142.6, 139.1, 136.1, 131.4, 130.7, 130.6, 130.0, 128.5, 123.6, 120.1, 118.1, 114.8, 112.4, 99.0, 89.0, 71.9, 56.0, 53.2, 51.4, 47.8, 45.5, 3.2; * **HRMS** *m*/*z*: calcd for C_29_H_26_N_4_O_4_ [M + H]+, calcd for 495.2027; found, 495.2029; **Retention Time** (min) 22.38 (5–95% ACN 0.1% TFA in H_2_O 0.1% over 50 min), 99% purity. *Azetidine CH_2_ signals nonequivalent.

### 4-[2-(5-{[(1-Cyanoazetidin-3-yl)­amino]­methyl}-2-methoxyphenyl)-1H-pyrrolo­[2,3-*b*]­pyridin-4-yl]­benzoic acid (**43**)

To a solution of 4-[2-(5-{[(azetidin-3-yl)­amino]­methyl}-2-methoxyphenyl)-1H-pyrrolo­[2,3-*b*]­pyridin-4-yl]­benzoic acid (10 mg, 0.024 mmol, 1.0 equiv) in DMF (480 μL, 0.05 M) was added triethylamine (3.6 μL, 0.026 mmol, 1.1 equiv) and cyanogen bromide (2.5 mg, 0.024 mmol, 1.0 equiv) and stirred at room temperature for 18 h. The crude mixture was then purified by reverse phase HPLC (5–95% MeCN 0.1% TFA in H_2_O 0.1% over 90 min) to yield yellow solid **60** (2.2 mg, 20% yield); ^**1**^ **H NMR (400 MHz, DMSO-*d***_**6**_ **)** δ: 12.04 (d, *J* = 2.2 Hz, 1H, NH), 8.82 (s, 1H, CO_2_H), 8.34 (d, *J* = 5.0 Hz, 1H, H-6), 8.15 (d, *J* = 8.4 Hz, 2H, H-2’’& H-6″), 7.94 (d, *J* = 8.4 Hz, 2H, H-3′’& H-5′’), 7.90 (d, *J* = 2.2 Hz, 1H, H-6′), 7.39 (dd, *J* = 8.5, 2.2 Hz, 1H, H-4′), 7.27 (d, *J* = 5.0 Hz, 1H, H-5), 7.22 (d, *J* = 8.5 Hz, 1H, H-3′), 7.14 (d, *J* = 2.1 Hz, 1H, H-3), 4.36 – 4.26 (m, 3H, CH, CH_2_), 4.18 – 4.08 (s, 2H, CH_2_), 4.03 – 3.91 (m, 5H, CH_2,_ CH_3_); ^**13**^ **C NMR (101 MHz, DMSO-*d***_**6**_ **)** δ: 167.1 (C), 156.6 (C), 149.6 (C), 143.2 (C), 142.7 (CH), 138.9 (C), 135.9 (C), 130.5 (C), 130.1 (CH), 130.0 (CH), 129.1 (CH), 128.5 (CH), 127.0 (C), 120.1 (C), 118.1 (C), 114.8 (C), 114.6 (CH), 112.3 (CH), 99.5 (CH), 55.9 (CH_3_), 53.4 (CH_2_), 51.0 (2 x CH_2_), 50.2 (CH); **HRMS** *m/z*: calcd for C_26_H_23_N_5_O_3_ [M + H]+, 454.1873; found, 454.1869; **Retention Time** (min) 21.64 (5–95% MeCN 0.1% TFA in H_2_O 0.1% over 60 min, 254 nm), 97% purity.

### *tert*-Butyl [2-(3-bromo-4-methoxyphenoxy)­ethyl]­carbamate (**47**)

To a solution of *N*-Boc-ethanolamine **44** (500 mg, 3.1 mmol, 1.0 equiv) in anhydrous DCM (6.2 mL, 0.5 M) at 0 °C was added triethylamine (0.5 mL, 3.7 mmol, 1.2 equiv) and methylene sulfonyl chloride (0.29 mL, 3.7 mmol, 1.2 equiv) and the reaction was left to stir at room temperature for 2 h. Another 0.1 mL (0.74 mmol, 0.24 equiv) of methylene sulfonyl chloride was then added to the reaction. After a further two ours, the reaction was partitioned between DCM and H_2_O, and the aqueous layer was extracted three times with DCM. The organic layers were then dried over sodium sulfate and concentrated in vacuo to yield brown oil **45**.

The crude mixture **45** (721 mg, 3.0 mmol, 2.0 equiv) was then dissolved in anhydrous DMF (15 mL, 0.2 M) and treated with 3-bromo-4-methoxy phenol (306 mg, 1.5 mmol, 1.0 equiv) and cesium carbonate (1.96 g, 6.0 mmol, 4.0 equiv) and then heated at 70 °C for 72 h. The mixture was left to cool to room temperature, concentrated in vacuo and then partitioned between ethyl acetate and H_2_O. The aqueous layer was extracted three times with ethyl acetate and the organic layers were dried over sodium sulfate then concentrated in vacuo once again to yield compound **47** as a brown oil (526 mg, 49% yield); ^1^H NMR (400 MHz, CDCl_3_) δ: 7.09 (d, *J* = 2.4 Hz, 1H), 6.83 – 6.72 (m, 2H), 5.04 (br, 1H), 3.92 (t, *J* = 5.2 Hz, 2H), 3.81 (s, 3H), 3.47 (q, *J* = 5.2 Hz, 2H), 1.43 (s, 9H); ^13^C NMR (101 MHz, CDCl_3_) δ: 156.0, 153.0, 150.6, 120.0, 114.2, 112.9, 112.0, 79.6, 68.0, 56.9, 40.1, 28.4; ; **HRMS** *m*/*z*: calcd for C_9_H_12_NO_3_Br [M-Boc + H]+ calc for 246.0124 found 246.132.

### *tert*-Butyl N-{2-[4-methoxy-3-(4,4,5,5-tetramethyl-1,3,2-dioxaborolan-2-yl)­phenoxy]­ethyl}­carbamate (**48**)

To a solution of compound **47** (100 mg, 0.3 mmol, 1.0 equiv) in anhydrous dioxane (3 mL, 0.1 M) was added B_2_Pin_2_ (110 mg, 0.45 mmol, 1.5 equiv) and KOAc (85 mg, 0.9 mmol, 3.0 equiv). The solution was sparged with nitrogen for 5 min and Pd­(dppf)­Cl_2_·CH_2_Cl_2_ (24 mg, 0.03 mmol, 10 mol %) was added, before heating at reflux for 18 h. The resulting solution was partitioned between ethyl acetate and H_2_O, and the aqueous layer was extracted three times with ethyl acetate. The organic layers were dried over sodium sulfate and the filtrate was concentrated in vacuo. Purification by automated flash column chromatography on an Isolera one (30–60% EtOAc in petroleum ether) yielded colorless oil **48** (54.5 mg, 48% yield); ^1^H NMR (400 MHz, CDCl_3_) δ: 7.20 (d, *J* = 3.2 Hz, 1H), 6.91 (dd, *J* = 8.9, 3.2 Hz, 1H), 6.78 (d, *J* = 8.9 Hz, 1H), 5.02 (br s, 1H), 3.97 (t, *J* = 5.1 Hz, 2H), 3.77 (s, 3H), 3.48 (q, *J* = 5.1 Hz, 2H), 1.44 (s, 9H), 1.34 (s, 12H); ^13^C NMR (101 MHz, CDCl_3_) δ: 158.9, 156.0, 152.3, 122.4, 118.4, 112.2, 83.7, 79.5, 67.9, 60.5, 56.8, 40.4, 28.5, 24.7; **HRMS** *m*/*z*: calcd for C_20_H_32_BNO_6_Na [M + Na]+ calc for 416.2219 found 416.2221.

### N-(2-{3-[4-Bromo-1-(4-methylbenzenesulfonyl)-1H-pyrrolo­[2,3-*b*]­pyridin-2-yl]-4-methoxyphenoxy}­ethyl)­carbamate (**50**)

To a solution of compound **49**, synthesized according to the protocol of our previous work,^4,19^ (165 mg, 0.35 mmol, 1.0 equiv) in dioxane (3.5 mL, 0.1 M) and 2 M Na_2_CO_3_ (1.2 mL, 2.45 mmol, 7.0 equiv) was added compound **48** (150 mg, 0.38 mmol, 1.1 equiv). The solution was sparged with nitrogen for 5 min before Pd­(PPh_3_)_4_ (40 mg, 0.035 mmol, 10 mol %) was added. The solution was then heated for 18 h at 110 °C, before being allowed to cool to room temperature and partitioned between ethyl acetate and H_2_O. The aqueous layer was extracted three times with ethyl acetate and the organic layers were washed with brine and dried over magnesium sulfate. The filtrate was concentrated in vacuo and purified by automated flash column chromatography on an Isolera one (20–60% EtOAc in petroleum ether) yielded colorless oil **50** (192 mg, 90% yield); ^1^H NMR (400 MHz, CDCl_3_) δ: 8.20 (d, *J* = 5.3 Hz, 1H), 7.88 (d, *J* = 8.4 Hz, 2H), 7.33 (d, *J* = 5.3 Hz, 1H), 7.20 (d, *J* = 8.4 Hz, 2H), 6.99 (dd, *J* = 8.9, 3.0 Hz, 1H), 6.94 (d, *J* = 3.0 Hz, 1H), 6.89 (d, *J* = 8.9 Hz, 1H), 6.53 (s, 1H), 5.05 (br s, 1H), 4.03 (t, *J* = 5.1 Hz, 2H), 3.75 (s, 3H), 3.55 (q, *J* = 5.1 Hz, 2H), 2.35 (s, 3H), 1.46 (s, 9H); ^13^C NMR (101 MHz, CDCl_3_) δ: 156.0, 152.9, 152.0, 148.8, 145.0, 144.6, 139.0, 136.2, 129.3, 128.3, 125.0, 123.5, 122.6, 122.3, 118.0, 116.1, 111.4, 107.5, 68.0, 56.0, 40.4, 28.2, 25.0, 21.7; **HRMS** *m*/*z*: calcd for C_28_H_30_N_3_O_6_BrS [M + H]+, calcd for 618.1095; found, 618.1100.

### Methyl 4-{2-[5-(2-{[(tert-butoxy)­carbonyl]­amino}­ethoxy)-2-methoxyphenyl]-1-(4-methylbenzenesulfonyl)-1H-pyrrolo­[2,3-*b*]­pyridin-4-yl}­benzoate (**51**)

To a solution of compound **50** (134 mg, 0.22 mmol, 1.0 equiv) in dioxane (2.2 mL, 0.1 M) and 2 M Na_2_CO_3_ (0.55 mL, 1.1 mmol, 5.0 equiv) was added methyl 4-(4,4,5,5-tetramethyl-1,3,2-dioxaborolan-2-yl)­benzoate **31** (86 mg, 0.33 mmol, 1.5 equiv). The solution was sparged with nitrogen for 5 min before Pd­(dppf)­Cl_2_·C_2_H_2_ (9 mg, 0.01 mmol, 5 mol %) was added. The solution was then heated in the microwave for 45 min at 110 °C, before being allowed to cool to room temperature and partitioned between ethyl acetate and H_2_O. The aqueous layer was extracted three times with ethyl acetate and the organic layers were washed with brine and dried over magnesium sulfate. The filtrate was concentrated in vacuo and purified by automated flash column chromatography on an Isolera one (40–70% EtOAc in petroleum ether) yielding colorless oil **51** (108 mg, 74% yield); ^1^H NMR (400 MHz, CDCl_3_) δ: 8.48 (d, *J* = 5.0 Hz, 1H), 8.13 (d, *J* = 8.4 Hz, 2H), 7.95 (d, *J* = 8.5 Hz, 2H), 7.67 (d, *J* = 8.4 Hz, 2H), 7.25 (d, *J* = 5.8 Hz, 1H), 7.22 (d, *J* = 8.5 Hz, 2H), 6.98 (dd, *J* = 8.8, 3.0 Hz, 1H), 6.94 (d, *J* = 3.0 Hz, 1H), 6.88 (d, *J* = 8.8 Hz, 1H), 6.65 (s, 1H), 5.04 (br s, 1H), 4.02 (t, *J* = 5.2 Hz, 2H), 3.94 (s, 3H), 3.75 (s, 3H), 3.54 (q, *J* = 5.2 Hz, 2H), 2.35 (s, 3H), 1.46 (s, 9H); **HRMS** *m*/*z*: calcd for C_36_H_37_N_3_O_8_S [M + H]+, calcd for 671.2534; found, 671.2416.

### 4-{2-[5-(2-Aminoethoxy)-2-methoxyphenyl]-1H-pyrrolo­[2,3-*b*]­pyridin-4-yl}­benzoic acid (**52**)

Compound **51** (81 mg, 0.12 mmol, 1.0 equiv) was dissolved in MeOH (1.5 mL, 0.08 M) and treated with potassium hydroxide (34 mg, 0.6 mmol, 5.0 equiv) and heated to reflux for 18 h. The crude mixture was then concentrated in vacuo and then treated with TFA (2.5 mL, 0.05 M). After two hours, the solution was concentrated in vacuo and then coevaporated with MeOH. The resulting residue was taken forward to the next step without further purification.

### 4-(2-{2-Methoxy-5-[2-(prop-2-enamido)­ethoxy]­phenyl}-1H-pyrrolo­[2,3-*b*]­pyridin-4-yl)­benzoic acid (**53**)

Compound **52** (12 mg, 0.03 mmol, 1.0 equiv) was dissolved in anhydrous DMF (0.5 mL, 0.06 M) and was treated with triethylamine (30 μL, 0.18 mmol, 6.0 equiv) and acryloyl chloride (7.5 μL, 0.09 mmol, 3.0 equiv) and stirred at room temperature. After 1 h, the reaction was resubjected to the conditions above. After a further hour of stirring at room temperature, the solution was quenched with H_2_O and the crude reaction mixture was then purified by reverse phase HPLC (20–60% MeCN 0.1% TFA in H_2_O) to yield yellow solid **53** (4.1 mg, 30% yield); ^**1**^ **H NMR (400 MHz, DMSO)** δ: 12.06 (s, 1H), 8.39 (t, *J* = 5.6 Hz, 1H), 8.14 (d, *J* = 8.4 Hz, 2H), 7.94 (d, *J* = 8.4 Hz, 2H), 7.54 (d, *J* = 3.0 Hz, 1H), 7.27 (d, *J* = 4.8 Hz, 1H), 7.23 (s, 1H), 7.10 (d, *J* = 9.0 Hz, 1H), 6.96 (dd, *J* = 9.0, 3.0 Hz, 1H), 6.29 (dd, *J* = 17.1, 10.1 Hz, 1H), 6.12 (dd, *J* = 17.1, 2.3 Hz, 1H), 5.60 (dd, *J* = 10.1, 2.3 Hz, 1H), 4.09 (t, *J* = 5.7 Hz, 2H), 3.86 (s, 3H), 3.54 (q, *J* = 5.7 Hz, 2H); ^**13**^ **C NMR (101 MHz, DMSO)** δ: 167.0, 164.9, 152.4, 151.0, 149.3, 142.9, 142.6, 138.9, 136.1, 131.6, 130.5, 130.0, 128.5, 125.3, 120.3, 118.3, 115.7, 114.7, 113.8, 113.3, 99.9, 66.9, 56.1, 38.4; **HRMS** *m*/*z*: calcd for C_26_H_23_N_3_O_5_ [M + H]+, calcd for 458.1710; found, 458.1718; **Retention Time** (min) 24.95 (5–95% ACN 0.1% TFA in H_2_O 0.1% over 50 min), 99% purity.

### 4-[2-(2-Methoxy-5-{2-[(2*E*)-4-methoxy-4-oxobut-2-enamido]­ethoxy}­phenyl)-1H-pyrrolo­[2,3-*b*]­pyridin-4-yl]­benzoic acid (**54**)

A solution of monomethyl fumarate (13 mg, 0.14 mmol, 5.0 equiv), DIPEA (46 μL, 0.27 mmol, 10.0 equiv) and HATU (46 mg, 0.14 mmol, 5.0 equiv) in DMF (540 μL, 0.25 M) was stirred at room temperature for 30 min. Compound **52** (11 mg, 0.027 mmol, 1.0 equiv) was added and the solution was stirred overnight. The crude reaction mixture was then purified by reverse phase flash column chromatography on an Isolera one using a 30g C18 column (5–95% ACN 0.1% TFA in H_2_O 0.1%) to yield a yellow solid (3.1 mg, 22% yield); ^**1**^ **H NMR (400 MHz, DMSO)** δ: 12.06 (d, *J* = 2.1 Hz, 1H), 8.84 (t, *J* = 5.6 Hz, 1H), 8.33 (d, *J* = 5.0 Hz, 1H), 8.14 (d, *J* = 8.5 Hz, 2H), 7.94 (d, *J* = 8.5 Hz, 2H), 7.54 (d, *J* = 2.9 Hz, 1H), 7.27 (d, *J* = 5.0 Hz, 1H), 7.22 (d, *J* = 2.1 Hz, 1H), 7.10 (d, *J* = 9.0 Hz, 1H), 7.09 (d, *J* = 15.5 Hz, 1H), 6.96 (dd, *J* = 9.0, 2.9 Hz, 1H), 6.62 (d, *J* = 15.5 Hz, 1H), 4.12 (t, *J* = 5.6 Hz, 2H), 3.86 (s, 3H), 3.72 (s, 3H), 3.58 (q, *J* = 5.6 Hz, 2H); ^**13**^ **C NMR (101 MHz, DMSO)** δ: 167.0, 165.5, 163.1, 152.3, 151.1, 149.3, 142.8, 142.6, 138.9, 137.5, 136.1, 130.5, 130.0, 128.4, 128.2, 120.3, 118.3, 115.7, 114.6, 113.9, 113.3, 99.9, 66.7, 56.1, 52.0, 38.8; HRMS *m*/*z*: calcd for C_28_H_25_N_3_O_7_ [M + H]+, calcd for 516.1765; found, 516.1761; **Retention Time** (min) 27.60 (5–95% MeCN 0.1% TFA in H_2_O 0.1% over 50 min, 254 nm), 95% purity.

### Protein Purification

As previously described full-length *Pf*CLK3 construct was expressed in*E. coli*strain C43 (DE3).^4^ The protein was purified using IMAC, TEV cleavage, and a second IMAC step before dialyzing the protein into a final buffer containing 20 mM HEPES pH 7.4, 150 mM NaCl, 1 mM TCEP and 1 mM MgCl_2_. PfCLK3 kinase domain (residues 334–699 with a C-terminal TEV cleavage sequence and His6-tag) was cloned into pFastBac vector and expressed and purified from Sf21 insect cells. Cells were infected using P2 BIICs at an MOI of 0.2 and left to express for 72 h. Harvested cells were lysed and centrifuged before purifying using IMAC and SEC in a final buffer containing 20 mM HEPES pH 7.4, 150 mM NaCl, 1 mM TCEP, and 1 mM MgCl_2_.

### Protein Mass Spectrometry

For intact protein mass spectrometry, compounds were incubated with a 5-fold excess of compound in buffer containing 20 mM HEPES pH 7.4, 150 mM NaCl, 1 mM TCEP, and 1 mM MgCl_2_. Five μL of kinase domain (1 mg/mL) was added to 25 μL of buffer before 0.12 μL of compound in a 10 mM DMSO stock was added to start the incubation. Samples were incubated at room temperature for 4 h before 10 μL alliquots were analyzed by LC–MS. Intact protein LC–MS experiments were performed on a Synapt G2 Q-ToF instrument equipped with electrospray ionization (Waters Corp., Manchester, UK). LC separation was achieved using an Acquity UPLC equipped with a reverse phase C4 Aeris Widepore 50 × 2.1 mm HPLC column (Phenomenex, CA, USA) and a gradient of 5–95% acetonitrile (0.1% formic acid) over 10 minutes was employed. Data analysis was performed using MassLynx v4.1 and deconvolution was performed using MaxEnt. Mass adduct percentages were determined by calculating the percentage of protein covalently modified from the MaxEnt deconvoluted LC–MS data.

### Time Resolved Förster Resonance Energy Transfer Assay

Time resolved förster resonance energy transfer (Tr-FRET) technology was used to determine kinase activity and Km ATP before profiling compounds. An 11-point half-log serial dilution of each test compound was prepared in 100% DMSO at 100x the final test concentration. The dilution series were dispensed on to assay plates with liquid handling machinery using positive displacement. The final DMSO concentration in the assay was 1%. To obtain IC_50_ values, full length recombinant *Pf*CLK3 was prepared in 1x assay buffer (50 mM Hepes, 10 mM MgCl_2_, 1 mM EGTA, 0.01% Tween20, 2 mM TCEP) at a 2x concentration (50 nM) and incubated with compounds for 15 min at room temperature before addition of ULight-labeled peptide substrate (MBP) with Km ATP (at 2x concentration). The reaction was centrifuged and incubated for 2 h at 37 °C. To stop the reaction, 1x Lance detection buffer was added with 1 nM Eu-labeled anti-MBP and 10 mM EDTA, and the reaction was incubated in the dark for 2 h at room temperature to allow detection to take place. Plate output was read on a Pherastar FSX and data analyzed in Graphpad Prism to a 4-parameter curve fit. Reactions were normalized to kinase and no kinase control wells.

### *P. falciparum* (3D7 and Dd2) Culture and Synchronization

*P. falciparum* cultures were maintained in RPMI-1640 media (with l-glutamine) (Invitrogen) supplemented with Albumax II (0.25%), hypoxanthine (0.05 mg/mL), HEPES (25 mM) and gentamicin (0.01 mg/mL). For continuous culture, the parasites were maintained at 4% hematocrit in human erythrocytes from O+ male blood donors and between 0.5–3% parasitaemia in an incubator at 37 °C, 5% CO2, 5% O2 and 90% N2. To obtain highly synchronous ring stage parasites for assays, cultures were double synchronized using Percoll and D-sorbitol synchronization. First, highly segmented schizonts were enriched by centrifugation on a 70% Percoll (GE Healthcare) cushion gradient. The schizont pellet was collected and washed twice before fresh erythrocytes were added to a final hematocrit of 4%, and incubated for about 3 h to enable merozoites to egress and reinvade new erythrocytes. Residual schizonts were then removed by a second Percoll purification followed by treating the ring pellet with D-sorbitol to select for 0–3 h old ring-stage parasites.

### *P. Falciparum* (3D7 and Dd2) Inhibition Assay

To determine the EC50 of the molecules of asexual blood stage*P. falciparum*3D7 and Dd2 a Mosquito Liquid Hangling System (sptlabtech) was used to perform 7-point (one in ten) serial dilutions of each compound, dispensing 300 nL per well, in triplicate across black, clear-bottomed experimental 384-well plates to give final assay concentrations ranging from 100 μM–100 pM. TCMDC-135051 and artemisinin were used as positive controls and DMSO as a negative control. Chloroquine was used as an additional control for the chloroquine-resistant Dd2 line. Thirty microlitres of 0.4%*P. falciparum*rings (0–3 h) in culture media at 4% hematocrit were added per well and mixed with compound by pipetting up and down. Final DMSO concentration in the assay was 1%. Plates were then incubated at 37 °C, 5% CO2, 5% O2 and 90% N2 for 72 ± 2 h (1.5 life cycles) before being frozen (−20 °C). To quantify growth inhibition, plates were thawed at room temperature for at least 1 h prior to the addition of 30 μL of lysis buffer (20 mM Tris–HCl; 5 mM EDTA; 0.004% saponin and triton X-100 in 1x PBS) containing Sybr-green (1 μL per 5 mL) to each well. Contents of each well were pipetted up and down to ensure mixing. Plates were incubated in the dark at room temperature for 1 h before reading the absorbance at excitation 485 nM and emission 520 nM using a PHERAstar *FSX* (BMG LABTECH). Four-parameter nonlinear regression curves were fitted to generate EC50 values using GraphPad Prism 10. Data were normalized to TCMDC-135051 controls. Three biological replicates, each with three technical replicates were performed per compound.

### Human Cell Viability Assay

Mycoplasma tested HepG2 cells were cultured in Dulbecco’s Modified Eagle Medium (DMEM) with 10% fetal bovine serum, 1% nonessential AA, 1% sodium pyruvate, 1% Penstrep and 100 μg/mL Normocin. THP-1 cells were cultured in Roswell Park Memorial Institute (RPMI) 1640 medium (+l-glutamine) with 10% fetal bovine serum and 1% Penstrep. Cultures were incubated at 37 °C, 5% CO2. HepG2 cells were detached using 0.05% trypsin–EDTA. 500 nL of drug compound dilutions, in triplicate, were added to 384-well, black, clear bottom assay plates using a Mosquito liquid handling machine. Assay plates, containing compound dilutions, were seeded at 5000 cells/well and incubated for 24 (THP-1) or 48 h (HepG2). 40 μM final concentration resazurin, diluted in DPBS, was added to the assay plates which were then incubated for 4 h and analyzed using a ClariostarTM plate reader to measure fluorescence Intensity (545–20 nm/600–40 nm). Control compounds included on every HepG2 assay plate were Tamoxifen, Puromycin and TCMDC-135051. Maximum signal control was obtained from wells with DMSO only and minimum signal control with Tamoxifen 100 μM. Control compounds included on every THP-1 assay plate were Puromycin and Staurosporine. Maximum signal control was obtained from wells with DMSO only and minimum signal control with Puromycin 100 μM. These were used to normalize data and give percentage inhibition of metabolic activity. Experiments were performed *N* = 3 and normalized data was grouped and a nonlinear regression curve with four parameters was plotted using GraphPad Prism, generating activity data.

### Metabolic Stability

Stability in human serum was evaluated using HPLC. To an microfuge tube containing 15 μL compound (10 mM in DMSO) in a preheated block (37 °C) was added 135 μL PBS buffer and 150 μL serum from human male AB plasma (USA origin, sterile-filtered, H4522) to initiate the reaction (0.5 mM compound and 5% DMSO end concentration). The reaction was incubated at 37 °C. At each time point (2 min–24 h), 12.5 μL was removed and 87.5 μL of cold MeOH (−20 °C, 2% TFA) was added to quench the reaction. Samples were centrifuged (1 min, 4000*g*), and 10 μL was then injected onto a Shimadzu HPLC to run over 15 min (5–95% ACN +0.1% TFA in H2O + 0.1% TFA). Consumption of starting material was then monitored by UV compound **4** was monitored by a wavelength of 254 nm. The natural log of the peak area was plotted against time to yield a straight line. The gradient of this line was taken as the pseudo-first order rate constant, and *t*_1/2_ was obtained by dividing −ln (2) by this value.

Glutathione stability was determined using HPLC. To an microfuge containing 125 μL compound (10 mM in DMSO) in a preheated block (37 °C) was added 1125 μL GSH (11.1 mM in 5 mM phosphate buffer, pH 7.5) to initiate the reaction. The reaction was incubated at 37 °C, and 100 μL aliquots were removed at time points (2 min–72 h). To each time point sample, 900 μL cold MeOH (−20 °C, 0.1% formic acid) was added to quench the reaction. 40 μL was then injected onto a Shimadzu HPLC to run over 15 min (5–95% ACN + 0.1% TFA in H2O + 0.1% TFA). Consumption of starting material was then monitored by UV (214 nm) and the natural log of the peak area was plotted against time to yield a straight line. The gradient of this line was taken as the pseudo-first order rate constant, and *t*_1/2_ was obtained by dividing −ln (2) by this value.

Stability assays in hepatocytes were carried out by Pharmaron Ltd. For hepatocyte stability, 198 μL of hepatocytes and boiled hepatocytes in William’s E Medium supplemented with GlutaMAX were added to a 96-well noncoated plate, and incubated at 37 °C for 10 min. Two μL of 100 μM test compound or positive control was added start the reaction, followed by further incubation. Well contents were transferred in 25 μL aliquots at time points of 0.5, 15, 30, 60, 90, and 120 min. The aliquots were then mixed with 6 volumes (150 μL) of acetonitrile containing with internal standard, IS (100 nM alprazolam, 200 nM caffeine and 100 nM tolbutamide) to terminate the reaction. Samples were vortexed for 5 min and centrifuged for 45 min at 3220*g*. 100 μL of the supernatant was diluted in 100 μL ultrapure water, and the mixture was used for LC/MS/MS analysis. All incubations were performed in duplicate. Peak areas were determined from extracted ion chromatograms. The slope value, *k*, was determined by linear regression of the natural logarithm of the remaining percentage of the parent drug vs incubation time curve.

## Supplementary Material









## Notes

Experimental procedures and characterization data for all compounds; metabolic stability data for all tested compounds (PDF)
Molecular docking of compounds **13**, **15** and **16** (PDB)
Molecular docking of compounds **2**, **17**, **19**, **20**, **22** and **25** (PDB)
Molecular formula strings (CSV)

## Abbreviations

ATP: adenosine triphosphate
DMSO: dimethysulfoxide
MOE: molecular operating environment
PfCLK3: *Plasmodium falciparum* cyclin-dependent like kinase
TCMDC: Tres Cantos antimalarial set
TR-FRET: time resolved forster resonance energy transfer.
