# Supplementary material for: Shaping Antimalarials: A Geometry-First Approach to PfCLK3 Covalent Inhibitors
Source: J Med Chem. 2026 Jan 31;69(3):3378–95. doi: 10.1021/acs.jmedchem.5c03342 (PMC12910650; doi:10.1021/acs.jmedchem.5c03342)

Supporting Information

Shaping Antimalarials: a Geometry-First Approach to *Pf*CLK3 Covalent Inhibitors

Skye B. Brettell,† Carla Fuentes-Guerra Bustos,† Saumya Sharma,§ Gillian Cann,§ Lauren V. Carruthers,§ Abbey Begen,‡§ Graeme Milligan,‡ § David J Clarke,¥ Andrew B. Tobin,‡ § and Andrew G. Jamieson†§*

†School of Chemistry, The Advanced Research Centre, University of Glasgow, 11 Chapel Lane, G11 6EW, U.K. ‡Centre for Translational Pharmacology, The Advanced Research Centre, University of Glasgow, 11 Chapel Lane, G11 6EW, U.K. §Keltic Pharma Therapeutics, The Advanced Research Centre, University of Glasgow, 11 Chapel Lane, G11 6EW, U.K. ¥EaSTCHEM School of Chemistry, University of Edinburgh, Joseph Black Building, David, Brewster Road, Edinburgh, EH9 3FJ, U.K. *andrew.jamieson.2@glasgow.ac.uk

Contents

Small-Molecule Synthesis and Characterization 1

Metabolic Stability..16

NMR Spectra for Novel Compounds 18

HPLC traces for Novel Compounds...45

Small-Molecule Synthesis and Characterization.

Small molecules mentioned in this study were synthesized, with their purity and identity validated using ^{1}H and ^{13}C NMR, HPLC and HRMS. All tested compounds are >95% pure by HPLC Analysis. Methods and characterization of newly synthesized small molecules are supplied in the Chemical Synthesis and Characterization Data section of the Supporting Information.

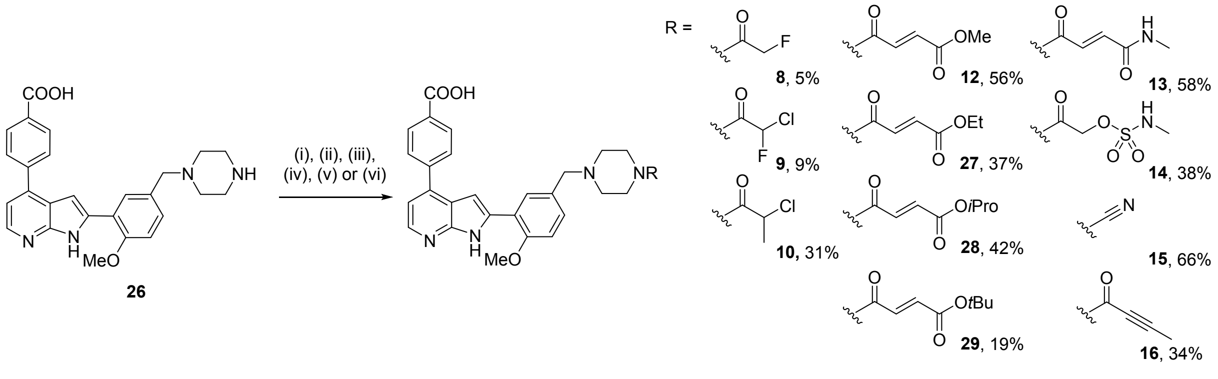

4-(2-{2-Methoxy-5-[(piperazin-1-yl)methyl]phenyl}-1H-pyrrolo[2,3-b]pyridin-4-yl)benzoic acid (26)

Intermediate 26 was synthesised using the protocol from our previous work. All characterisation was in accordance with this literature.[4]

4-[2-(5-{[4-(2-Fluoroacetyl)piperazin-1-yl]methyl}-2-methoxyphenyl)-1H-pyrrolo[2,3-b]pyridin-4-yl]benzoic acid (8)

Compound **26** (16 mg, 0.04 mmol, 1.0 equiv.) was dissolved in anhydrous DMF and treated with chloroacetyl chloride (4 µL, 0.06 mmol, 1.5 equiv.) and triethylamine (30 µL, 0.24 mmol, 6 equiv.) and stirred at room temperature for 2 hours. The reaction was then treated with tetrabutylammonium fluoride (TBAF) (2.0 M in THF, 113 µL, 0.22 mmol, 5.0 equiv.). After 18 hours the crude reaction mixture was therefore purified by reverse phase flash column chromatography on an Isolera one with a 25 g C18 column (5-95% MeCN 0.1% TFA in H_2O 0.1%) to yield yellow solid **49** (1.0 mg, 4.5% yield). **^{1}H NMR (400 MHz, DMSO)** δ 12.01 (s, 1H), 8.35 (d, *J* = 4.9 Hz, 1H), 8.14 (dd, *J* = 8.2, 1.4 Hz, 2H), 7.96 – 7.89 (m, 3H), 7.49 (d, *J* = 8.5 Hz, 1H), 7.32 – 7.24 (m, 2H), 7.08 (s, 1H), 5.16 (dd, *J* = 43.8, 14.6 Hz, 2H), 4.33 (s, 2H), 3.96 (s, 3H), 3.42 (br s, 4H), 3.00 (br s, 4H); **^{19}F NMR (377 MHz, DMSO)** δ -229.58; **HRMS** m/z calcd for $C_{28}H_{27}N_4O_4F$ [M+H]+ calc for 503.2089 found 503.2103; **Retention Time** (min) 21.62 (5-95% ACN 0.1% TFA in H_2O 0.1% over 50 minutes), 96% purity.

4-[2-(5-{[4-(2-Chloro-2-fluoroacetyl)piperazin-1-yl]methyl}-2-methoxyphenyl)-1H-pyrrolo[2,3-b]pyridin-4-yl]benzoic acid (9)

A solution of racemic chlorofluoroacetic acid (60 µL, 0.81 mmol, 18 equiv.) and DIPEA (154 µL, 0.90 mmol, 20 equiv.) was added T3P (50% v/v solution in EtOAc, 482 µL, 18 equiv.) and stirred at 0 °C for 1 hour. Compound **26** (20 mg, 0.045 mmol, 1 equiv.) was then added and the mixture was heated at 50 °C. After 18 hours, conversion to product was determined to be 25%, with conversion to an unknown side product at 20%. The crude reaction mixture was therefore purified by reverse phase flash column chromatography on an Isolera one with a 25g C18 column (5-95 ACN 0.1% TFA in H_2O 0.1%) to yield a yellow solid (2.1 mg, 9% yield). **^{1}H NMR (400 MHz, DMSO)** δ 12.02 (s, 1H), 8.35 (d, *J* = 5.0 Hz, 1H), 8.14 (d, *J* = 8.0 Hz, 2H), 7.93 (d, *J* = 8.4 Hz, 3H), 7.50 (d, *J* = 8.5 Hz, 1H), 7.36 – 7.19 (m, 3H), 7.09 (s, 1H), 4.35 (s, 2H), 3.96 (s, 3H), 3.44 (br s, 4H), 3.26 – 2.99 (br s, 4H); **^{19}F NMR (377 MHz, DMSO)** δ -145.33. **HRMS** m/z calcd for $C_{28}H_{26}N_4O_4ClF$ [M+H]+ calc for 537.1699 found 537.1697; **Retention Time** (min) 23.20 (5-95% ACN 0.1% TFA in H_2O 0.1% over 50 minutes), 95% purity.

4-[2-(5-{[4-(2-Chloropropanoyl)piperazin-1-yl]methyl}-2-methoxyphenyl)-1H-pyrrolo[2,3-b]pyridin-4-yl]benzoic acid (10)

Compound **26** (13 mg, 0.0275 mmol, 1.0 equiv.) in anhydrous DMF (500 µL, 0.1 M) was treated with NEt_3 (4 µL, 0.034 mmol, 1.25 equiv.) and 2-chloropropionyl chloride (3 µL, 0.034 mmol, 1.25 equiv.). After 1 hour at room temperature, the reaction was re-treated with 2 equiv.alents (0.055 mmol) of each reagent. A further 5 equivalents (0.138 mmol) of each reagent was added one hour later, and after a subsequent hour, the reaction was determined to have completed by LCMS analysis.

The crude reaction mixture was then purified by reverse phase flash column chromatography on an Isolera one using a 30g C18 column (5-70 ACN 0.1% TFA in H_2O 0.1%) to yield a yellow solid (4.5 mg, 31% yield). **^{1}H NMR (400 MHz, DMSO)** δ 12.06 (d, *J* = 2.1 Hz, 1H), 10.14 (s, 1H), 8.36 (d, *J* = 5.0 Hz, 1H), 8.15 (d, *J* = 8.4 Hz, 2H), 7.97 – 7.89 (m, 3H), 7.51 (dd, *J* = 8.7, 2.0 Hz, 1H), 7.32 – 7.25 (m, 2H), 7.09 (d, *J* = 2.1 Hz, 1H), 5.08 (s, 1H), 4.36 (s, 2H), 3.96 (s, 3H), 3.55 – 2.95 (m, 6H), 1.52 (d, *J* = 6.4 Hz, 3H**)**; **^{13}C NMR (101 MHz, DMSO)** δ 167.0, 157.3, 149.4, 143.1, 142.6, 139.0, 135.8, 132.7, 131.9, 130.5, 130.0, 128.4, 120.2, 118.0, 114.8, 112.4, 99.3, 82.5, 58.5, 56.0, 50.5, 50.2, 50.1. 42.2, 20.7; **HRMS** m/z calcd for $C_{29}H_{29}N_4O_4Cl$ [M+H]+ calc for 533.1950 found 533.1957; **Retention Time** (min) 23.22 (5-95% ACN 0.1% TFA in H_2O 0.1% over 60 minutes), 99% purity.

4-{2-[2-Methoxy-5-({4-[(2*E*)-4-methoxy-4-oxobut-2-enoyl]piperazin-1-yl}methyl)phenyl]-1H-pyrrolo[2,3-b]pyridin-4-yl}benzoic acid (12)

To a solution of monomethyl fumarate (24 mg, 0.18 mmol, 4 equiv.) in DMF (1.5 ml, 0.12 M) was added DIPEA (31 µL, 0.18 mmol, 4 equiv.) and HATU (69 mg, 0.18 mmol, 4 equiv.). The reaction was stirred at room temperature for 15 minutes, during which time the yellow solution went golden brown. Compound **26** (20 mg, 0.045 mmol, 1 equiv.) was then added, and the reaction stirred for a further hour. The crude reaction mixture was then purified by reverse phase flash column chromatography on an Isolera one using a 30g C18 column (10-60% ACN 0.1% TFA in H_2O 0.1%) to yield a yellow solid (13.5 mg, 56% yield); **^{1}H NMR (400 MHz, DMSO)** δ 12.05 (d, *J* = 2.2 Hz, 1H), 10.16 (s, 1H), 8.36 (d, *J* = 5.0 Hz, 1H), 8.15 (d, *J* = 8.4 Hz, 2H), 7.96 – 7.89 (m, 3H), 7.50 (dd, *J* = 8.5, 2.2 Hz, 1H), 7.48 (d, *J* = 15.4, 1H), 7.32 – 7.25 (m, 2H), 7.09 (d, *J* = 2.0 Hz, 1H), 6.61 (d, *J* = 15.4 Hz, 1H), 4.34 (s, 2H), 3.96 (s, 3H), 3.73 (s, 3H), 3.44 (br s, 4H), 3.07 (br s, 4H); **^{13}C NMR (101 MHz, DMSO)** δ 167.0, 165.4, 162.9, 157.3, 149.4, 143.0, 142.6, 139.1, 135.8, 134.2, 132.7, 131.9, 130.6, 130.2, 130.0, 128.4, 121.4, 120.2, 118.1, 114.8, 112.4, 99.3, 58.5, 54.0, 52.2, 50.7, 50.1. ; **HRMS** m/z calcd for $C_{31}H_{30}N_4O_6$ [M+H]+ calc for 555.2238 found 555.2236; **Retention Time** (min) 23.60 (5-95% ACN 0.1% TFA in H_2O 0.1% over 50 minutes), 99% purity.

4-{2-[5-({4-[(2*E*)-4-Ethoxy-4-oxobut-2-enoyl]piperazin-1-yl}methyl)-2-methoxyphenyl]-1H-pyrrolo[2,3-b]pyridin-4-yl}benzoic acid (27)

To a solution of mono-ethyl fumarate (20 mg, 0.125 mmol, 5 equiv.) in DMF (500 µL, 0.25 M) was added DIPEA (43 µL, 0.25 mmol, 10 equiv.) and T3P (50 wt% in EtOAc, 74 µL, 0.125 mmol, 5 equiv.). The reaction was stirred at room temperature for 15 minutes, during which time the yellow solution went golden brown. Compound **26** (11 mg, 0.025 mmol, 1 equiv.) was then added, and the reaction stirred for a further hour. The crude reaction mixture was then purified by reverse phase HPLC (20-70% ACN 0.1% TFA in H_2O 0.1%) to yield a yellow solid (6.7 mg, 37% yield); **^{1}H NMR (400 MHz, DMSO)** δ 12.02 (s, 1H), 8.35 (d, *J* = 5.0 Hz, 1H), 8.14 (d, *J* = 8.4 Hz, 2H), 7.95 (d, J = 2.2, 1H), 7.93 (d, *J* = 8.4, 2H), 7.51 (dd, *J* = 8.5, 2.2 Hz, 1H), 7.46 (d, *J* = 15.4 Hz, 1H), 7.32 – 7.25

(m, 2H), 7.10 (d, *J* = 1.9 Hz, 1H), 6.69 (d, *J* = 1.1 Hz, 1H), 6.59 (d, *J* = 15.4 Hz, 1H), 4.34 (s, 2H), 4.19 (q, *J* = 7.1 Hz, 2H), 3.96 (s, 3H), 1.24 (t, *J* = 7.1 Hz, 3H);* **^{13}C NMR (101 MHz, DMSO)** δ 167.1, 165.7, 164.9, 163.0, 157.3, 149.4, 143.1, 142.6, 139.1, 135.9, 134.6, 134.1, 132.7, 131.9, 130.6, 130.0, 128.5, 120.2, 118.1, 114.8, 112.4, 99.3, 61.0, 58.6, 56.0, 14.0; **HRMS** m/z calcd for $C_{32}H_{32}N_4O_6$ [M+H]+ calc for 569.2395 found 569.2391; **Retention Time** (min) 24.08 (5-95% ACN 0.1% TFA in H_2O 0.1% over 50 minutes), 99% purity *Piperazine peaks occluded by H_2O signals

4-{2-[5-({4-[(2*E*)-4-*iso*-Propoxy-4-oxobut-2-enoyl]piperazin-1-yl}methyl)-2-methoxyphenyl]-1H-pyrrolo[2,3-b]pyridin-4-yl}benzoic acid (28)

To a solution of mono-ispropyl fumarate (22 mg, 0.138 mmol, 5 equiv.) in DMF (550 µL, 0.5 M) was added DIPEA (47 µL, 0.275 mmol, 10 equiv.) and T3P (50 wt% in EtOAc, 81 µL, 0.138 mmol, 5 equiv.). The reaction was stirred at room temperature for 30 minutes, during which time the yellow solution went golden brown. Compound **26** (13 mg, 0.0275 mmol, 1 equiv.) was then added, and the reaction stirred for a further hour. The crude reaction mixture was then purified by reverse phase HPLC (25-75% ACN 0.1% TFA in H_2O 0.1%) to yield a yellow solid (6.4 mg, 42% yield); **^{1}H NMR (400 MHz, DMSO)** δ 12.03 (s, 1H), 8.36 (d, *J* = 4.7 Hz, 1H), 8.15 (d, *J* = 8.4, 2H), 7.97 – 7.89 (m, 3H), 7.51 (dd, *J* = 8.5, 2.2 Hz, 1H), 7.43 (d, *J* = 15.4 Hz, 1H), 7.32 – 7.25 (m, 2H), 7.09 (d, *J* = 1.9 Hz, 1H), 6.56 (d, *J* = 15.4 Hz, 1H), 5.00 (hept, *J* = 6.3 Hz, 1H), 4.34 (s, 3H), 3.96 (s, 3H), 3.47 (br s, 4H), 3.10 (br s, 4H), 1.24 (d, *J* = 6.3 Hz, 6H); **^{13}C NMR (101 MHz, DMSO)** δ 167.0, 164.4, 163.0, 157.3, 146.9, 143.1, 142.6, 139.0, 135.8, 133.9, 132.6, 131.9, 130.9, 130.5, 130.0, 128.4, 121.3, 120.2, 118.0, 114.8, 112.4, 99.3, 68.4, 58.5, 56.0, 50.6, 50.1, 21.5; **HRMS** m/z calcd for $C_{33}H_{34}N_4O_6$ [M+H]+ calc for 583.2551 found 583.2552; **Retention Time** (min) 25.37 (5-95% ACN 0.1% TFA in H_2O 0.1% over 50 minutes), 100% purity

4-{2-[5-({4-[(2*E*)-4-*tert*-Butoxy-4-oxobut-2-enoyl]piperazin-1-yl}methyl)-2-methoxyphenyl]-1H-pyrrolo[2,3-b]pyridin-4-yl}benzoic acid (29)

To a solution of mono-*tert*-butyl fumarate (23 mg, 0.134 mmol, 5 equiv.) in DMF (1.07 mL, 0.125 M) was added DIPEA (45 µL, 0.267 mmol, 10 equiv.) and HATU (51 mg, 0.134 mmol, 5 equiv.). The reaction was stirred at room temperature for 30 minutes, during which time the yellow solution went golden brown. Compound **26** (12 mg, 0.0267 mmol, 1 equiv.) was then added, and the reaction stirred for a further hour. The crude reaction mixture was then purified by reverse phase HPLC (20-60% ACN 0.1% TFA in H_2O 0.1%) to yield a yellow solid (3.1 mg, 19% yield); **^{1}H NMR (400 MHz, DMSO)** δ 12.01 (d, *J* = 2.1 Hz, 1H), 10.00 (s, 1H), 8.35 (d, *J* = 5.0 Hz, 1H), 8.14 (d, *J* = 8.4, 2H), 7.92 (m, 3H), 7.50 (dd, *J* = 8.5, 2.2 Hz, 1H), 7.36 (d, *J* = 15.4 Hz, 1H), 7.31 – 7.25 (m, 2H), 7.08 (d, *J* = 2.1 Hz, 1H), 6.50 (d, *J* = 15.4 Hz, 1H), 4.34 (s, 2H), 3.96 (s, 3H), 1.46 (s, 9H);* **^{13}C NMR (101**

MHz, DMSO) δ 167.0, 164.1, 163.1, 158.2, 157.3, 149.5, 143.2, 142.6, 139.0, 135.8, 133.2, 132.6, 132.2, 131.8, 130.5, 130.0, 128.4, 120.3, 118.0, 114.8, 112.4, 99.3, 81.2, 58.6, 56.0, 27.6;* **HRMS** m/z calcd for $C_{34}H_{36}N_4O_6$ [M+H]+ calc for 597.2708 found 597.2717; **Retention Time** (min) 26.76 (5-95% ACN 0.1% TFA in H_2O 0.1% over 50 minutes), 99% purity. *Piperazine peaks not visible due to amide rotamer broadening.

4-{2-[2-Methoxy-5-({4-[(2*E*)-3-(methylcarbamoyl)prop-2-enoyl]piperazin-1-yl}methyl)phenyl]-1H-pyrrolo[2,3-b]pyridin-4-yl}benzoic acid (13)

To a solution of (E)-4-(methylamino)-4-oxobut-2-enoic acid (17 mg, 0.134 mmol, 5 equiv.) in DMF (1.07 mL, 0.125 M) was added DIPEA (45 µL, 0.267 mmol, 10 equiv.) and HATU (46 mg, 0.121 mmol, 4.5 equiv.). The reaction was stirred at room temperature for 30 minutes, during which time the colourless solution became deep purple. Compound **26** (12 mg, 0.0267 mmol, 1 equiv.) was then added, and the reaction stirred for a further 18 hours. The conversion was determined to be 50% by LCMS, and so the reaction was re-subjected to the conditions stated above. After a further 18 hours, the crude reaction mixture was then purified by reverse phase flash column chromatography on an Isolera one using a 30g C18 column (5-95% ACN 0.1% TFA in H_2O 0.1%) to yield a yellow solid (10.3 mg, 58% yield); **^{1}H NMR (400 MHz, DMSO)** δ 12.06 (d, *J* = 2.1 Hz, 1H), 10.15 (s, 1H), 8.43 (q, *J* = 4.7 Hz, 1H), 8.36 (d, *J* = 5.0 Hz, 1H), 8.15 (d, *J* = 8.4 Hz, 2H), 7.96 – 7.89 (m, 3H), 7.50 (dd, *J* = 8.6, 2.1 Hz, 1H), 7.32 – 7.23 (m, 3H), 7.09 (d, *J* = 2.1 Hz, 1H), 6.85 (d, *J* = 15.0 Hz, 1H), 4.34 (s, 2H), 3.95 (s, 3H), 3.43 (s, 4H), 3.04 (s, 4H), 2.69 (d, *J* = 4.7 Hz, 3H); **^{13}C NMR (101 MHz, DMSO)** δ 167.0, 164.0, 163.6, 157.3, 149.4, 143.1, 142.6, 139.1, 135.9, 135.5, 132.6, 131.9, 130.6, 130.1, 128.5, 127.9, 121.4, 120.2, 118.1, 114.8, 112.4, 99.3, 58.5, 56.0, 25.8;* **HRMS** m/z calcd for $C_{31}H_{31}N_5O_5$ [M+H]+ calc for 554.2398 found 554.2400; **Retention Time** (min) 21.84 (5-95% ACN 0.1% TFA in H_2O 0.1% over 50 minutes), 95% purity. *Piperazine peaks not visible due to amide rotamer broadening.

4-(2-{5-[(4-{2-[(*tert*-Butyldiphenylsilyl)oxy]acetyl}piperazin-1-yl)methyl]-2-methoxyphenyl}-1H-pyrrolo[2,3-b]pyridin-4-yl)benzoic acid (S1)

To a solution of 2-((t-butyldiphenylsilyl)oxy)acetic acid (173 mg, 0.55 mmol, 5.0 equiv.) in DMF (1.0 mL, 0.5 M) was added DIPEA (190 µL, 1.1 mmol, 10.0 equiv.) and HATU (168 mg, 0.44 mmol, 4.0 equiv.). The mixture was stirred at room temperature for 30 minutes before compound **26** (50 mg, 0.11 mmol, 1.0 equiv.) was added, and the reaction was stirred for a further 18 hours at room temperature. The crude reaction mixture was then purified by reverse phase flash column chromatography on an Isolera one using a 30g C18 column (5-95 ACN 0.1% TFA in H_2O 0.1%) to yield a yellow solid (43 mg, 81% yield). **^{1}H NMR (400 MHz, DMSO)** δ 12.06 – 12.01 (m, 1H), 10.16 (s, 1H), 8.36 (d, *J* = 5.0 Hz, 1H), 8.15 (d, *J* = 8.6 Hz, 2H), 7.97 – 7.87 (m, 3H), 7.66 – 7.59 (m, 4H), 7.51 – 7.35 (m, 7H), 7.28 (m, 2H), 7.09 (d, *J* = 2.0 Hz, 1H), 4.42 (s, 2H), 4.33 (s, 2H), 3.95 (s,

3H), 3.52 – 3.25 (br s, 4H), 2.96 (br s, 4H), 0.97 (s, 9H); **^{13}C NMR (101 MHz, DMSO)** δ 168.1, 167.0, 157.3, 149.4, 143.1, 142.6, 139.0, 135.8, 135.1, 134.5, 132.6, 130.5, 130.0, 128.4, 127.9, 127.5, 121.2, 120.2, 118.0, 114.8, 112.4, 99.3, 62.8, 58.5, 56.0, 26.5, 18.9; **HRMS** m/z calcd for $C_{44}H_{46}N_4O_5Si$ [M-H]- calc for 737.3165 found 737.3157.

4-(2-{2-Methoxy-5-[(4-{2-[(methylsulfamoyl)oxy]acetyl}piperazin-1-yl)methyl]phenyl}-1H-pyrrolo[2,3-b]pyridin-4-yl)benzoic acid (14)

To a solution of **S1** (15 mg, 0.02 mmol, 1 equiv.) in DMF (676 μL, 0.03 M) was added (31 mg, 0.2 mmol, 10.0 equiv.) and the reaction was heated at 60 ℃ for 6 hours under inert atmosphere. After cooling to room temperature, the crude reaction mixture was then concentrated *in vacuo*, redissolved in anhydrous DMF (100 μL, 0.2 M) and treated with DIPEA (84 μL, 0.48 mmol, 24.0 equiv.) and methyl sulfamoyl chloride (126 mmol, 1.44 mmol, 72.0 equiv.). The reaction was stirred at room temperature for 1 hour. The crude reaction mixture was then quenched with H_2O (100 μL) and purified by reverse phase flash column chromatography on an Isolera one using a 30 g C18 column (5-95 MeCN 0.1% TFA in H_2O 0.1%). The crude reaction mixture was then purified by reverse phase flash column chromatography on an Isolera one using a 30 g C18 column (5-95 MeCN 0.1% TFA in H_2O 0.1%) to yield yellow solid (5.6 mg, 47% yield, 38% yield from compound **26**). **^{1}H NMR (400 MHz, DMSO)** δ 12.03 (s, 1H), 8.36 (d, *J* = 5.0 Hz, 1H), 8.14 (d, *J* = 8.2 Hz, 2H), 7.97 – 7.89 (m, 3H), 7.83 (q, *J* = 4.8 Hz, 1H), 7.51 (dd, *J* = 8.6, 2.2 Hz, 1H), 7.32 – 7.25 (m, 2H), 7.09 (d, *J* = 2.0 Hz, 1H), 4.81 (s, 2H), 4.35 (s, 2H), 3.96 (s, 3H), 3.42 (br s, 4H), 3.01 (br s, 4H), 2.60 (d, *J* = 4.8 Hz, 3H); **^{13}C NMR (101 MHz, DMSO)** δ 167.0, 164.1, 157.3, 149.5, 142.6, 139.0, 135.8, 132.7, 130.5, 130.0, 128.4, 120.3, 118.0, 114.8, 112.4, 107.2, 101.1, 99.3, 88.5, 75.0, 65.6, 56.0, 50.1, 28.9; **HRMS** m/z calcd for $C_{29}H_{31}N_5O_7S$ [M-H]- calc for 592.1871 found 592.1874; **Retention Time** (min) 23.62 (5-95% ACN 0.1% TFA in H_2O 0.1% over 50 minutes), 97% purity.

4-(2-{5-[(4-Cyanopiperazin-1-yl)methyl]-2-methoxyphenyl}-1H-pyrrolo[2,3-b]pyridin-4-yl)benzoic acid (15)

Compound **26** (13 mg, 0.0275 mmol, 1.0 equiv.) in DMF (500 μL, 0.1 M) and aqueous sodium hydrogen carbonate (36 μL) was treated with cyanogen bromide (3.2 mg, 0.025 mmol, 0.9 equiv.) and stirred at room temperature for 18 hours, during which time the yellow reaction mixture turned vivid orange. The crude reaction mixture was then purified by reverse phase flash column chromatography on an Isolera one using a 30g C18 column (10-60% ACN 0.1% TFA in H_2O 0.1%) to yield a yellow solid (8.15 mg, 66% yield); **^{1}H NMR (400 MHz, DMSO)** δ 12.06 (s, 1H), 8.36 (d, *J* = 5.0 Hz, 1H), 8.14 (d, *J* = 8.4 Hz, 2H), 7.97 – 7.89 (m, 3H), 7.50 (dd, *J* = 8.5, 2.2 Hz, 1H), 7.32 – 7.24 (m, 2H), 7.10 (d, *J* = 1.7 Hz, 1H), 4.35 (s, 2H), 3.95 (s, 3H), 3.29 (br s, 4H);* **^{13}C NMR (101 MHz, DMSO)** δ

167.2, 157.4, 149.2, 143.0, 142.6, 139.4, 136.1, 132.9, 132.0, 130.7, 130.2, 128.6, 121.4, 120.3, 118.3, 116.4, 115.0, 112.5, 99.4, 58.8, 56.1, 49.5, 45.6; **HRMS** m/z calcd for $C_{27}H_{25}N_5O_3$ [M+H]+ calc for 468.2030 found 468.2027; **Retention Time** (min) 22.17 (5-95% ACN 0.1% TFA in H_2O 0.1% over 50 minutes), 99% purity. *One piperazine peak occluded by H_2O signal.

4-[2-(5-{[4-(But-2-ynoyl)piperazin-1-yl]methyl}-2-methoxyphenyl)-1H-pyrrolo[2,3-b]pyridin-4-yl]benzoic acid (16)

A solution of 2-butynoic acid (22 mg, 0.27 mmol, 5.0 equiv), DIPEA (94 µL, 0.54 mmol, 10.0 equiv) and T3P (50% v/v solution in EtOAc, 163 µL, 0.27 mmol, 5.0 equiv) was stirred in DMF (1.08 mL, 0.25 M) at room temperature for 30 minutes before compound **26** (24 mg, 0.054 mmol, 1 equiv.) was added. After 1 hour, the crude reaction mixture was then purified by reverse phase flash column chromatography on an Isolera one using a 30g C18 column (5-70% ACN 0.1% TFA in H_2O 0.1%) to yield a yellow solid (9.3 mg, 34% yield); **^{1}H NMR (400 MHz, DMSO)** δ 12.10 (d, *J* = 2.0 Hz, 1H), 10.21 (s, 1H), 8.37 (d, *J* = 5.0 Hz, 1H), 8.15 (d, *J* = 5.0 Hz, 2H), 7.98 – 7.89 (m, 3H), 7.51 (dd, *J* = 8.6, 2.1 Hz, 1H), 7.32 – 7.25 (m, 2H), 7.10 (d, *J* = 2.0 Hz, 1H), 4.34 (s, 2H), 3.96 (s, 3H), 3.44 (br s, 4H), 3.06 (br s, 4H), 2.03 (s, 3H); **^{13}C NMR (101 MHz, DMSO)** δ 167.0, 157.3, 152.0, 149.2, 142.9, 142.6, 139.2, 135.9, 132.7, 131.9, 130.6, 130.1, 128.5, 121.4, 120.2, 118.2, 114.8, 112.4, 99.4, 90.6, 72.2, 58.5, 56.0, 50.5, 50.0, 3.4; **HRMS** m/z calcd for $C_{30}H_{28}N_4O_4$ $[M+2H]^{2+}$ calc for 255.1128 found 255.1124; **Retention Time** (min) 22.90 (5-95% ACN 0.1% TFA in H_2O 0.1% over 50 minutes), 99% purity.

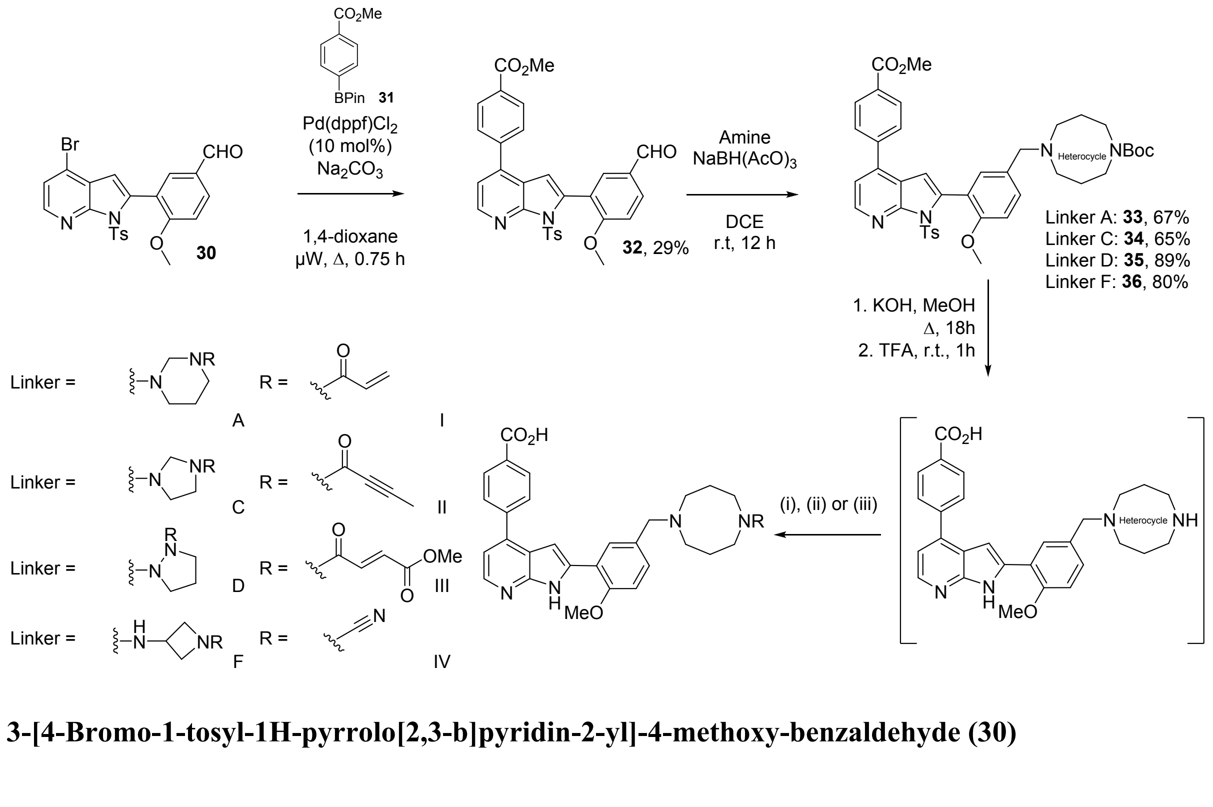

3-[4-Bromo-1-tosyl-1H-pyrrolo[2,3-b]pyridin-2-yl]-4-methoxy-benzaldehyde (30)

Intermediate **30** was synthesised using the protocol from our previous work. All characterisation was in accordance with this literature.[4, 19]

Methyl-4-[2-(5-formyl-2-methoxyphenyl)-1-(4-methylbenzenesulfonyl)-1H-pyrrolo[2,3b]pyridin-4-yl]benzoate (32)

To a 35 mL microwave vial containing **30** (2.3 g, 4.8 mmol, 1.0 eq), methyl 4-(4,4,5,5-tetramethyl-1,3,2-dioxaborolan-2-yl)benzoate **31**, (1.4 g, 5.3 mmol, 1.1 eq) and Pd(dppf)Cl_2·CH_2Cl_2 complex (0.2 g, 0.2 mmol, 0.05 eq) in 1,4-dioxane under nitrogen was added 2 M aqueous sodium carbonate (12.0 mL, 23.9 mmol, 5.0 eq). The reaction mixture was purged with nitrogen for 10 min and microwaved at 110 ºC for 45 min. The reaction mixture was diluted with 1:1 water:ethyl acetate and two layers were separated. The aqueous layer was extracted with ethyl acetate and the combined organic layers were dried over magnesium sulphate and concentrated *in vacuo* to give a brown solid. This was purified using automated flash column chromatography eluting with 25-50% ethyl acetate:petroleum ether. The desired fractions were combined and concentrated *in vacuo* to give compound **32** as a light brown solid (0.7 g, 29%). **R_f**: 0.5 (50% EtOAc in petroleum ether); **^{1}H NMR (400 MHz, $CDCl_3$)** δ 9.96 (s, 1H), 8.50 (d, *J* = 5.0 Hz, 1H), 8.14 (dt, *J* = 8.4, 1.8 Hz, 2H), 8.00 (dd, *J* = 8.5, 2.1 Hz, 1H), 7.91 (d, *J* = 2.1 Hz, 1H), 7.87 (dt, *J* = 8.2, 1.8 Hz, 2H), 7.68 (dt, *J* = 8.4, 1.8 Hz, 2H), 7.27 (d, *J* = 5.0 Hz, 1H), 7.22 (d, *J* = 8.2 Hz, 2 x CH, 2H), 7.11 (d, *J* = 8.5 Hz, 1H), 6.70 (s, 1H), 3.94 (s, 3H), 3.93 (s, 3H), 2.36 (s, 3H).**^{13}C NMR (101 MHz, $CDCl_3$)** δ 190.5, 166.6, 163.5, 149.7, 145.0, 144.9, 142.0, 141.2, 137.6, 136.0, 134.3, 131.2, 130.3, 130.2, 129.3, 129.3, 128.5, 128.1, 123.5, 119.7, 118.2, 110.5, 107.4, 56.1, 52.3, 21.6. **HRMS** *m/z calcd* for $C_{30}H_{24}N_2O_6S$ $(M + H)^+$ 541.1421 *found* 541.1423.

tert-butyl 3-[(4-Methoxy-3-{4-[4-(methoxycarbonyl)phenyl]-1-(4-methylbenzenesulfonyl)-1H-pyrrolo[2,3-b]pyridin-2-yl}phenyl)methyl]-1,3-diazinane-1-carboxylate (33)

To a solution of **32** (0.05 g, 0.09 mmol, 1.0 eq) in anhydrous dichloroethane (3 mL) was added tert-butyl-1,3-diazinane-1-carboxylate (0.05 g, 0.28 mmol, 3.0 eq) and titanium isopropoxide (0.05 mL, 0.19 mmol, 2.0 eq) and the solution was stirred under argon at rt for 10 min. Sodium triacetoxyborohydride (0.05 mg, 0.23 mmol, 2.5 eq) was added and the solution was stirred under argon at rt for 3 hours. Sodium triacetoxyborohydride (0.02 mg, 0.09 mmol, 1.0 eq) was added and the solution was stirred under argon at rt for 18 hours. The solution was then quenched by the addition of ammonium hydroxide solution (5 mL) and the reaction mixture was diluted with 1:1 water:dichloromethane and two layers were separated. The aqueous layer was extracted with dichloromethane, and the combined organic layers were washed with brine, dried over magnesium sulphate, and concentrated *in vacuo* to give a colourless oil. This oil was purified using automated flash column chromatography eluting with 45-80% ethyl acetate:petroleum ether. The desired fractions were combined and concentrated *in vacuo* to give compound **33** as a colourless oil (0.04 g, 65%). **R_f:** 0.48 (60% EtOAc in petroleum ether). **^{1}H**

NMR (400 MHz, CDCl$_3$) δ 8.48 (d, *J* = 5.1 Hz, 1H), 8.13 (dt, *J* = 8.4, 2.0 Hz, 2H), 7.91 (dt, *J* = 8.2, 2.9 Hz, 2H), 7.68 (dt, *J* = 8.4, 2.0 Hz, 2H), 7.40 (dd, *J* = 8.4, 2.2 Hz, 1H), 7.32 (d, *J* = 2.2 Hz, 1H), 7.25 (d, *J* = 5.1 Hz, 1H), 7.20 (d, *J* = 8.2 Hz, 2H), 6.92 (d, *J* = 8.4 Hz, 1H), 6.66 (s, 1H), 4.20 (br s, 1H), 4.12 (br s, 1H), 3.94 (s, 3H), 3.78 (s, 3H), 3.63 (s, 2H), 3.51 (m, 2H), 2.75 (s, 2H), 2.35 (s, 3H), 1.65 (s, 2H), 1.41 (s, 9H). **^{13}C NMR (101 MHz, CDCl$_3$)** δ 166.7, 157.6, 154.5, 149.8, 144.5, 142.3, 140.8, 139.3, 136.4, 131.7, 131.4, 130.2, 129.2, 128.5, 128.2, 122.0, 119.9, 118.0, 110.2, 106.8, 79.7, 77.3, 55.6, 52.3, 51.3, 28.4, 21.6. **HRMS** *m/z calcd* for $C_{39}H_{42}N_4O_7S$ $(M + H)^+$ 711.2840 *found* 711.2839.

***tert*-Butyl 3-[(4-methoxy-3-{4-[4-(methoxycarbonyl)phenyl]-1-(4-methylbenzenesulfonyl)-1H-pyrrolo[2,3-b]pyridin-2-yl}phenyl)methyl]imidazolidine-1-carboxylate (34)**

To a solution of **32** (0.05 g, 0.09 mmol, 1.0 eq) in anhydrous dichloroethane (3 mL) was added tert-butyl imidazolidine-1-carboxylate (0.05 g, 0.28 mmol, 3.0 eq) and the solution was stirred under argon at rt for 10 min. Sodium triacetoxyborohydride (0.05 mg, 0.23 mmol, 2.5 eq) was added and the solution was stirred under argon at rt for 3 hours. Sodium triacetoxyborohydride (0.02 mg, 0.09 mmol, 1.0 eq) was added and the solution was stirred under argon at rt for 18 hours. The solution was then quenched by the addition of ammonium hydroxide solution (5 mL) and the reaction mixture was diluted with 1:1 water:dichloromethane and two layers were separated. The aqueous layer was extracted with dichloromethane, and the combined organic layers were washed with brine, dried over magnesium sulphate and concentrated *in vacuo* to give a colourless oil. This oil was purified using automated flash column chromatography eluting with 45-80% ethyl acetate:petroleum ether. The desired fractions were combined and concentrated *in vacuo* to give compound **34** as a colourless oil (0.06 mg, 89%). **R_f:** 0.21 (45% EtOAc in petroleum ether). **^{1}H NMR (400 MHz, CDCl$_3$)** δ 8.49 (d, *J* = 5.1 Hz, 1H), 8.13 (d, *J* = 8.1 Hz, 2H), 7.91 (dt, *J* = 8.2, 2.0 Hz, 2H), 7.68 (d, *J* = 8.1 Hz, 2H), 7.40 (d, *J* = 7.8 Hz, 1H), 7.34 (d, *J* = 4.0 Hz, 1H), 7.25 (d, *J* = 5.1 Hz, 1H), 7.21 (d, *J* = 8.2 Hz, 2H), 6.94 (d, *J* = 7.8 Hz, 1H), 6.66 (s, 1H), 4.04 (d, *J* = 21.4 Hz, 2H), 3.94 (s, 3H), 3.79 (s, 3H), 3.67 (s, 2H), 3.47 (dt, *J* = 21.4, 7.0 Hz, 2H), 2.88 (t, *J* = 7.0 Hz, 2H), 2.35 (s, 3H), 1.44 (s, 9H). **^{13}C NMR (101 MHz, CDCl$_3$)** δ 166.6, 157.7, 153.6, 149.8, 144.6, 142.3, 140.9, 139.0, 136.4, 131.5, 131.1, 130.2, 129.2, 128.5, 128.2, 122.1, 119.9, 118.0, 110.3, 106.9, 77.2, 57.4, 55.6, 52.3, 28.5, 21.6. **HRMS** *m/z calcd* for $C_{38}H_{40}N_4O_7S$ $(M + H)^+$ 697.2675 *found* 697.2678.

tert-butyl 2-[(4-Methoxy-3-{4-[4-(methoxycarbonyl)phenyl]-1-(4-methylbenzenesulfonyl)-1H-pyrrolo[2,3-b]pyridin-2-yl}phenyl)methyl]pyrazolidine-1-carboxylate (35)

To a solution of **32** (0.1 g, 0.19 mmol, 1.0 eq) in anhydrous dichloroethane (5 mL) was added tert-butyl pyrazolidine-1-carboxylate (0.1 g, 0.56 mmol, 3.0 eq) and magnesium sulphate (0.1 g, 0.93 mmol, 5.0 eq) and the solution was stirred under argon at rt for 15 min. Sodium triacetoxyborohydride (0.1 g, 0.58 mmol, 2.5 eq) was added and the solution was stirred under argon at rt for 3 hours. Sodium

triacetoxyborohydride (0.04 g, 0.19 mmol, 1.0 eq) was added and the solution was stirred under argon at rt for 18 hours. The solution was then quenched by the addition of ammonium hydroxide solution (5 mL) and the reaction mixture was diluted with 1:1 water:dichloromethane and two layers were separated. The aqueous layer was extracted with dichloromethane, and the combined organic layers were washed with brine, dried over magnesium sulphate, and concentrated *in vacuo* to give a colourless oil. This oil was purified using automated flash column chromatography eluting with 40-70% ethyl acetate:petroleum ether. The desired fractions were combined and concentrated *in vacuo* to give compound **35** as a colourless oil (0.1 g, 80%). **R_f:** 0.33 (40% EtOAc in petroleum ether); **^{1}H NMR (400 MHz, $CDCl_3$)** δ 8.48 (d, *J* = 5.1 Hz, 1H), 8.13 (dt, *J* = 8.1 Hz, 2H), 7.91 (dt, *J* = 8.2, 2.0 Hz, 2H), 7.68 (d, *J* = 8.1 Hz, 2H), 7.46 – 7.43 (m, 1H), 7.42 – 7.40 (m, 1H), 7.24 (d, *J* = 5.1 Hz, 1H), 7.21 (d, *J* = 8.2 Hz, 2H), 6.92 (d, *J* = 7.8 Hz, 1H), 6.67 (s, 1H), 3.94 (s, 3H), 3.86 – 3.80 (m, 2H), 3.79 (s, 3H), 3.59 – 3.47 (m, 2H), 3.01 (t, *J* = 7.1 Hz, 2H), 2.35 (s, 3H), 2.07 (pent, *J* = 7.2 Hz, 2H), 1.47 (s, H-48, 9H). **^{13}C NMR (101 MHz, $CDCl_3$)** δ 166.7, 157.8, 149.7, 144.5, 144.5, 142.3, 140.8, 139.1, 136.4, 132.3, 132.0, 130.1, 129.2, 128.5, 128.2, 122.0, 119.9, 118.0, 110.2, 106.9, 80.0, 77.2, 60.0, 55.6, 52.3, 28.5, 23.9, 21.6; **HRMS** *m/z calcd* for $C_{38}H_{40}N_4O_7S$ $(M + H)^+$ 697.2684 *found* 697.2685.

***tert*-Butyl 3-{[(4-methoxy-3-{4-[4-(methoxycarbonyl)phenyl]-1-(4-methylbenzenesulfonyl)-1H-pyrrolo[2,3-b]pyridin-2-yl}phenyl)methyl]amino}azetidine-1-carboxylate (36)**

To a solution of compound **32** (21 mg, 0.04 mmol, 1.0 equiv.) in dichloroethane (0.4 mL, 0.1 M) was added 1-Boc-3-(amino)azetidine (22 mg, 0.12 mmol, 3.0 equiv.), Titanium isopropoxide (24 µL, 0.08 mmol, 2.0 equiv.) and sodium triacetoxyborohydride (21 mg, 0.1 mmol, 2.5 equiv.). The solution was allowed to stir for 3 hours at room temperature, before another equivalent of sodium triacetoxyborohydride (8 mg, 0.04 mmol) was added, and stirred for 18 hours. Ammonium hydroxide and water were then added, and the solution partitioned between the water and CH_2Cl_2. The aqueous layer was extracted with CH_2Cl_2 3 times, and the organic layers dried over magnesium sulfate.
The filtrate was concentrated *in vacuo* and purified by automated flash column chromatography on an Isolera one (0-10% MeOH in CH_2Cl_2) yielding colourless oil **36** (18 mg, 67% yield); **^{1}H NMR (400 MHz, CD_3OD)** δ 8.35 (d, *J* = 5.1 Hz, 1H), 8.10 (d, *J* = 8.5 Hz, 2H), 7.81 (d, *J* = 8.4 Hz, 2H), 7.69 (d, *J* = 8.5 Hz, 2H), 7.65 – 7.58 (m, 2H), 7.32 (d, *J* = 5.1 Hz, 1H), 7.29 (d, *J* = 8.4 Hz, 2H), 7.16 (d, *J* = 8.4 Hz, 1H), 6.71 (s, 1H), 4.28 (s, 2H), 4.26 – 4.14 (m, 3H), 4.00 (s, 2H), 3.91 (s, 3H), 3.79 (s, 3H), 2.33 (s, 3H), 1.40 (s, 9H); **^{13}C NMR (101 MHz, CD_3OD)** δ 160.8, 157.6, 150.9, 146.8, 145.7, 143.1, 142.7, 139.7, 137.2, 133.8, 133.1, 131.6, 131.2, 130.5, 129.9, 129.8, 129.0, 124.3, 123.3, 121.1, 119.6, 112.4, 108.6, 81.9, 56.2, 52.8, 50.1, 47.5, 28.5, 21.5;* **HRMS** *m/z calcd* for $C_{38}H_{40}N_4O_7S$ [M+H]+ 697.2690 *found* 697.2704. *Azetidine CH_2s not visible due to amide rotamer broadening.

4-[2-(5-{[(Azetidin-3-yl)amino]methyl}-2-methoxyphenyl)-1H-pyrrolo[2,3-b]pyridin-4-yl]benzoic acid (S2)

Compound **36** (22 mg, 0.035 mmol, 1.0 equiv.) was dissolved in MeOH (0.7 mL, 0.05 M) and treated with KOH (18 mg, 0.35 mmol, 10.0 equiv.) and heated to reflux for 18 hours. The solution was cooled to room temperature and concentrated *in vacuo*, before being treated with TFA (1.0 mL) for 1 hour. After concentration and co-evaporation with MeOH, the resulting residue was taken forward to the next step without further purification.

4-{2-[2-Methoxy-5-({[1-(prop-2-enoyl)azetidin-3-yl]amino}methyl)phenyl]-1H-pyrrolo[2,3-b]pyridin-4-yl}benzoic acid (40)

To a solution of **S2** (22 mg, 0.03 mmol, 1.0 equiv.) was dissolved in anhydrous DMF (632 µL, 0.05 M), and treated with DIPEA (32 µL, 0.18 mmol, 6.0 equiv.) and acryloyl chloride (4 µL, 0.05 mmol, 1.5 equiv.) and left to stir at room temperature for one hour. Another batch of acryloyl chloride (2.6 µL, 0.03 mmol, 1.0 equiv.) was added and left to stir for a further hour. The reaction was quenched with water and the crude reaction mixture was then purified by reverse phase flash column chromatography on an Isolera one using a 30g C18 column (5-95% ACN 0.1% TFA in H_2O 0.1%) to yield a yellow solid (3.7 mg, 24% yield); **^{1}H NMR (400 MHz, DMSO)** δ 11.97 (d, *J* = 2.1 Hz, 1H), 9.56 (s, 1H), 8.35 (d, *J* = 5.0 Hz, 1H), 8.18 – 8.11 (d, *J* = 8.4 Hz, 2H), 7.98 (d, *J* = 2.2 Hz, 1H), 7.93 (d, *J* = 8.4 Hz, 2H), 7.49 (dd, *J* = 8.5, 2.2 Hz, 1H), 7.31 – 7.23 (m, 2H), 7.08 (d, *J* = 2.1 Hz, 1H), 6.33 (dd, *J* = 17.0, 10.3 Hz, 1H), 6.29 (d, *J* = 10.3 Hz, 1H), 6.13 (dd, *J* = 17.0, 2.2 Hz, 1H), 5.72 (dd, *J* = 10.3, 2.2 Hz, 1H), 4.54 – 4.46 (m, 1H), 4.28 – 4.22 (m, 1H), 4.18 – 4.10 (m, 4H), 4.00 (d, *J* = 6.2, 1H), 3.96 (s, 3H), 3.91 (d, *J* = 6.8, 1H); **^{13}C NMR (101 MHz, DMSO)** δ 167.0, 164.8, 156.9, 149.5, 143.2, 142.6, 138.9, 136.0, 131.3, 130.5, 130.0, 128.4, 127.2, 126.6, 123.5, 120.1, 118.0, 114.8, 112.3, 99.0, 55.9, 53.2, 51.0, 48.5, 47.8, 45.6; **HRMS** m/z calcd for $C_{28}H_{26}N_4O_4$ [M+H]+ calc for 483.2027 found 484.2023; **Retention Time** (min) 21.93 (5-95% ACN 0.1% TFA in H_2O 0.1% over 50 minutes), 96% purity.

4-(2-{2-Methoxy-5-[({1-[(2*E*)-4-methoxy-4-oxobut-2-enoyl]azetidin-3-yl}amino)methyl]phenyl}-1H-pyrrolo[2,3-b]pyridin-4-yl)benzoic acid (41)

A solution of monomethyl fumarate (12 mg, 0.1 mmol, 5.0 equiv.), DIPEA (33 µL, 0.19 mmol, 10.0 equiv.) and propanephosphonic acid anhydride (50% v/v solution in EtOAc, 57 µL, 0.1 mmol, 5.0 equiv.) in DMF (380 µL, 0.05 M) was stirred at room temperature for 30 minutes. **S2** (8 mg, 0.019 mmol, 1.0 equiv.) was added and the solution stirred for a further 18 hours. The crude mixture was then purified by reverse phase flash column chromatography on an Isolera one using a 30 g C18 column (5-60% MeCN 0.1% TFA in H_2O 0.1%) to yield a yellow solid (7.2 mg, 93% yield); **^{1}H NMR (400 MHz, DMSO)** δ 12.04 (d, *J* = 2.1 Hz, 1H), 9.67 (s, 2H), 8.36 (d, *J* = 5.0 Hz, 1H), 8.15 (d,

J = 8.4 Hz, 2H), 7.99 (d, *J* = 2.2 Hz, 1H), 7.93 (d, *J* = 8.4 Hz, 2H), 7.50 (dd, *J* = 8.6, 2.2 Hz, 1H), 7.29 (d, *J* = 5.0 Hz, 1H), 7.27 (d, *J* = 8.6 Hz, 1H), 7.10 (d, *J* = 2.1 Hz, 1H), 6.99 (d, *J* = 15.5 Hz, 1H), 6.62 (d, *J* = 15.5 Hz, 1H), 4.67 – 4.58 (m, 1H), 4.39 (dd, *J* = 10.6, 4.0 Hz, 1H), 4.26 – 4.09 (m, 4H), 4.05 (dd, *J* = 10.6, 3.6 Hz, 1H), 3.96 (s, 3H), 3.73 (s, 3H); **^{13}C NMR (101 MHz, DMSO)** δ 167.0, 165.3, 162.9, 156.9, 149.2, 142.8, 142.6, 139.3, 136.2, 132.2, 131.4, 130.7, 130.6, 130.0, 129.5, 128.5, 123.6, 120.0, 118.2, 114.8, 112.4, 99.0, 56.0, 53.5, 52.2, 51.5, 47.8, 45.6; **HRMS** m/z calcd for $C_{30}H_{28}N_4O_6$ [M+H]+ calc for 541.2082 found 541.2076; **Retention Time** (min) 22.64 (5-95% ACN 0.1% TFA in H_2O 0.1% over 50 minutes), 99% purity. *Azetidine CH_2 signals non-equivalent.

4-{2-[5-({[1-(But-2-ynoyl)azetidin-3-yl]amino}methyl)-2-methoxyphenyl]-1H-pyrrolo[2,3-b]pyridin-4-yl}benzoic acid (42)

A solution of 2-butynoic acid (8 mg, 0.1 mmol, 5.0 equiv.), DIPEA (33 µL, 0.19 mmol, 10.0 equiv.) and T3P (50% v/v solution in EtOAc, 57 µL, 0.1 mmol, 5.0 equiv.) in DMF (380 µL, 0.05 M) was stirred at room temperature for 30 minutes. Compound **S2** (0.019 mmol, 1.0 equiv.) was added and the solution stirred for a further 18 hours. The crude mixture was then purified by reverse phase flash column chromatography on an Isolera one using a 30g C18 column (5-60% ACN 0.1% TFA in H_2O 0.1%) to yield a yellow solid (7.2 mg, 77% yield); **^{1}H NMR (400 MHz, DMSO)** δ 12.01 (d, *J* = 2.0 Hz, 1H), 9.61 (m, 2H), 8.36 (d, *J* = 5.0 Hz, 1H), 8.14 (d, *J* = 8.3 Hz, 2H), 7.99 (d, *J* = 2.2 Hz, 1H), 7.93 (d, *J* = 8.3 Hz, 2H), 7.50 (dd, *J* = 8.6, 2.2 Hz, 1H), 7.29 (d, *J* = 5.0 Hz, 1H), 7.26 (d, *J* = 8.6 Hz, 1H), 7.09 (d, *J* = 2.0 Hz, 1H), 4.39 (dd, *J* = 10.4, 6.6 Hz, 1H), 4.25 – 4.08 (m, 5H), 3.96 (m, 4H), 2.01 (s, 3H); **^{13}C NMR (101 MHz, DMSO)** δ 167.0, 156.9, 153.4, 149.3, 143.0, 142.6, 139.1, 136.1, 131.4, 130.7, 130.6, 130.0, 128.5, 123.6, 120.1, 118.1, 114.8, 112.4, 99.0, 89.0, 71.9, 56.0, 53.2, 51.4, 47.8, 45.5, 3.2;* **HRMS** m/z calcd for $C_{29}H_{26}N_4O_4$ [M+H]+ calc for 495.2027 found 495.2029; **Retention Time** (min) 22.38 (5-95% ACN 0.1% TFA in H_2O 0.1% over 50 minutes), 99% purity. *Azetidine CH_2 signals non-equivalent.

4-[2-(5-{[(1-Cyanoazetidin-3-yl)amino]methyl}-2-methoxyphenyl)-1H-pyrrolo[2,3-b]pyridin-4-yl]benzoic acid (43)

To a solution of 4-[2-(5-{[(azetidin-3-yl)amino]methyl}-2-methoxyphenyl)-1H-pyrrolo[2,3-b]pyridin-4-yl]benzoic acid (10 mg, 0.024 mmol, 1.0 equiv.) in DMF (480 µL, 0.05 M) was added triethylamine (3.6 µL, 0.026 mmol, 1.1 equiv.) and cyanogen bromide (2.5 mg, 0.024 mmol, 1.0 equiv.) and stirred at room temperature for 18 hours. The crude mixture was then purified by reverse phase HPLC (5-95% MeCN 0.1% TFA in H_2O 0.1% over 90 minutes) to yield yellow solid **60** (2.2 mg, 20% yield); **^{1}H NMR (400 MHz, DMSO-d6)** δ 12.04 (d, *J* = 2.2 Hz, 1H, N*H*), 8.82 (s, 1H, CO_2H), 8.34 (d, *J* = 5.0 Hz, 1H, H-6), 8.15 (d, *J* = 8.4 Hz, 2H, H-2''& H-6''), 7.94 (d, *J* = 8.4 Hz, 2H, H-3''& H-5''), 7.90 (d, *J* = 2.2 Hz, 1H, H-6'), 7.39 (dd, *J* = 8.5, 2.2 Hz, 1H, H-4'), 7.27 (d, *J* = 5.0 Hz, 1H, H-5), 7.22 (d, *J* = 8.5 Hz, 1H,

H-3'), 7.14 (d, J = 2.1 Hz, 1H, H-3), 4.36 – 4.26 (m, 3H, CH, CH_2), 4.18 – 4.08 (s, 2H, CH_2), 4.03 – 3.91 (m, 5H, CH_2, CH_3); **^{13}C NMR (101 MHz, DMSO-d6)** δ 167.1 (C), 156.6 (C), 149.6 (C), 143.2 (C), 142.7 (CH), 138.9 (C), 135.9 (C), 130.5 (C), 130.1 (CH), 130.0 (CH), 129.1 (CH), 128.5 (CH), 127.0 (C), 120.1 (C), 118.1 (C), 114.8 (C), 114.6 (CH), 112.3 (CH), 99.5 (CH), 55.9 (CH_3), 53.4 (CH_2), 51.0 (2 x CH_2), 50.2 (CH); **HRMS** *m/z calcd* for $C_{26}H_{23}N_5O_3$ [M+H]+ 454.1873 *found* 454.1869; **Retention Time** (min) 21.64 (5-95% MeCN 0.1% TFA in H_2O 0.1% over 60 minutes, 254 nm), 97% purity.

***tert*-Butyl [2-(3-bromo-4-methoxyphenoxy)ethyl]carbamate (47)**

To a solution of *N*-Boc-ethanolamine **44** (500 mg, 3.1 mmol, 1.0 equiv.) in anhydrous DCM (6.2 mL, 0.5 M) at 0 °C was added triethylamine (0.5 mL, 3.7 mmol, 1.2 equiv.) and methylene sulfonyl chloride (0.29 mL, 3.7 mmol, 1.2 equiv.) and the reaction was left to stir at room temperature for 2 hours. Another 0.1 mL (0.74 mmol, 0.24 equiv.) of methylene sulfonyl chloride was then added to the reaction. After a further two ours, the reaction was partitioned between DCM and H_2O, and the aqueous layer was extracted three times with DCM. The organic layers were then dried over sodium sulfate and concentrated *in vacuo* to yield brown oil **45**.
The crude mixture **45** (721 mg, 3.0 mmol, 2.0 equiv) was then dissolved in anhydrous DMF (15 ml, 0.2 M) and treated with 3-bromo-4-methoxy phenol (306 mg, 1.5 mmol, 1.0 equiv) and caesium carbonate (1.96 g, 6.0 mmol, 4.0 equiv.) and then heated at 70 °C for 72 hours. The mixture was left to cool to room temperature, concentrated *in vacuo* and then partitioned between ethyl acetate and H_2O. The aqueous layer was extracted 3 times with ethyl acetate and the organic layers were dried over sodium sulfate then concentrated *in vacuo* once again to yield compound **47** as a brown oil (526 mg, 49% yield); **^{1}H NMR (400 MHz, $CDCl_3$)** δ 7.09 (d, J = 2.4 Hz, 1H), 6.83 – 6.72 (m, 2H), 5.04 (br , 1H), 3.92 (t, J = 5.2 Hz, 2H), 3.81 (s, 3H), 3.47 (q, J = 5.2 Hz, 2H), 1.43 (s, 9H); **^{13}C NMR (101 MHz, $CDCl_3$)** δ 156.0, 153.0, 150.6, 120.0, 114.2, 112.9, 112.0, 79.6, 68.0, 56.9, 40.1, 28.4; ; **HRMS** m/z calcd for $C_9H_{12}NO_3Br$ [M-Boc+H]+ calc for 246.0124 found 246.132.

***tert*-Butyl N-{2-[4-methoxy-3-(4,4,5,5-tetramethyl-1,3,2-dioxaborolan-2-yl)phenoxy]ethyl}carbamate (48)**

To a solution of compound **47** (100mg, 0.3 mmol, 1.0 equiv.) in anhydrous dioxane (3 mL, 0.1 M) was added B_2Pin_2 (110 mg, 0.45 mmol, 1.5 equiv.) and KOAc (85 mg, 0.9 mmol, 3.0 equiv). The solution was sparged with nitrogen for 5 minutes and Pd(dppf)Cl_2.CH_2Cl_2 (24 mg, 0.03 mmol, 10 mol%) was added, before heating at reflux for 18 hours. The resulting solution was partitioned between ethyl acetate and H_2O , and the aqueous layer was extracted 3 times with ethyl acetate. The organic layers were dried over sodium sulfate and the filtrate was concentrated *in vacuo.* Purification by automated flash column chromatography on an Isolera one (30-60% EtOAc in petroleum ether) yielded colourless oil **48** (54.5 mg, 48% yield); **^{1}H NMR (400 MHz, $CDCl_3$)** δ 7.20 (d, J = 3.2 Hz,

1H), 6.91 (dd, *J* = 8.9, 3.2 Hz, 1H), 6.78 (d, *J* = 8.9 Hz, 1H), 5.02 (br s, 1H), 3.97 (t, *J* = 5.1 Hz, 2H), 3.77 (s, 3H), 3.48 (q, *J* = 5.1 Hz, 2H), 1.44 (s, 9H), 1.34 (s, 12H); **^{13}C NMR (101 MHz, $CDCl_3$)** δ 158.9, 156.0, 152.3, 122.4, 118.4, 112.2, 83.7, 79.5, 67.9, 60.5, 56.8, 40.4, 28.5, 24.7; **HRMS** m/z calcd for $C_{20}H_{32}BNO_6Na$ [M+Na]+ calc for 416.2219 found 416.2221.

N-(2-{3-[4-Bromo-1-(4-methylbenzenesulfonyl)-1H-pyrrolo[2,3-b]pyridin-2-yl]-4-methoxyphenoxy}ethyl)carbamate (50)

To a solution of compound **49**, synthesised according to the protocol of our previous work,[4,19] (165 mg, 0.35 mmol, 1.0 equiv) in dioxane (3.5 mL, 0.1 M) and 2M Na_2CO_3 (1.2 mL, 2.45 mmol, 7.0 equiv.) was added compound **48** (150 mg, 0.38 mmol, 1.1 equiv.). The solution was sparged with nitrogen for 5 minutes before $Pd(PPh_3)_4$ (40 mg, 0.035 mmol, 10 mol%) was added. The solution was then heated for 18 hours at 110 °C, before being allowed to cool to room temperature and partitioned between ethyl acetate and H_2O. The aqueous layer was extracted 3 times with ethyl acetate and the organic layers were washed with brine and dried over magnesium sulfate. The filtrate was concentrated *in vacuo* and purified by automated flash column chromatography on an Isolera one (20-60% EtOAc in petroleum ether) yielded colourless oil **50** (192 mg, 90% yield); **^{1}H NMR (400 MHz, $CDCl_3$)** δ 8.20 (d, *J* = 5.3 Hz, 1H), 7.88 (d, *J* = 8.4 Hz, 2H), 7.33 (d, *J* = 5.3 Hz, 1H), 7.20 (d, *J* = 8.4 Hz, 2H), 6.99 (dd, *J* = 8.9, 3.0 Hz, 1H), 6.94 (d, *J* = 3.0 Hz, 1H), 6.89 (d, *J* = 8.9 Hz, 1H), 6.53 (s, 1H), 5.05 (br s, 1H), 4.03 (t, *J* = 5.1 Hz, 2H), 3.75 (s, 3H), 3.55 (q, *J* = 5.1 Hz, 2H), 2.35 (s, 3H), 1.46 (s, 9H); **^{13}C NMR (101 MHz, $CDCl_3$)** δ 156.0, 152.9, 152.0, 148.8, 145.0, 144.6, 139.0, 136.2, 129.3, 128.3, 125.0, 123.5, 122.6, 122.3, 118.0, 116.1, 111.4, 107.5, 68.0, 56.0, 40.4, 28.2, 25.0, 21.7; **HRMS** m/z calcd for $C_{28}H_{30}N_3O_6BrS$ [M+H]+ calc for 618.1095 found 618.1100.

Methyl 4-{2-[5-(2-{[(tert-butoxy)carbonyl]amino}ethoxy)-2-methoxyphenyl]-1-(4-methylbenzenesulfonyl)-1H-pyrrolo[2,3-b]pyridin-4-yl}benzoate (51)

To a solution of compound **50** (134 mg, 0.22 mmol, 1.0 equiv) in dioxane (2.2 mL, 0.1 M) and 2M Na_2CO_3 (0.55 mL, 1.1 mmol, 5.0 equiv.) was added methyl 4-(4,4,5,5-tetramethyl-1,3,2-dioxaborolan-2-yl)benzoate **31** (86 mg, 0.33 mmol, 1.5 equiv.). The solution was sparged with nitrogen for 5 minutes before $Pd(dppf)Cl_2.C_2H_2$ (9 mg, 0.01 mmol, 5 mol%) was added. The solution was then heated in the microwave for 45 minutes at 110 °C, before being allowed to cool to room temperature and partitioned between ethyl acetate and H_2O. The aqueous layer was extracted 3 times with ethyl acetate and the organic layers were washed with brine and dried over magnesium sulfate. The filtrate was concentrated *in vacuo* and purified by automated flash column chromatography on an Isolera one (40-70% EtOAc in petroleum ether) yielding colourless oil **51** (108 mg, 74% yield); **^{1}H NMR (400 MHz, $CDCl_3$)** δ 8.48 (d, *J* = 5.0 Hz, 1H), 8.13 (d, *J* = 8.4 Hz, 2H), 7.95 (d, *J* = 8.5 Hz, 2H), 7.67 (d, *J* = 8.4 Hz, 2H), 7.25 (d, *J* = 5.8 Hz, 1H), 7.22 (d, *J* = 8.5 Hz, 2H), 6.98 (dd, *J* = 8.8, 3.0

Hz, 1H), 6.94 (d, *J* = 3.0 Hz, 1H), 6.88 (d, *J* = 8.8 Hz, 1H), 6.65 (s, 1H), 5.04 (br s, 1H), 4.02 (t, *J* = 5.2 Hz, 2H), 3.94 (s, 3H), 3.75 (s, 3H), 3.54 (q, *J* = 5.2 Hz, 2H), 2.35 (s, 3H), 1.46 (s, 9H); **HRMS** m/z calcd for $C_{36}H_{37}N_3O_8S$ [M+H]+ calc for 671.2534 found 671.2416.

4-{2-[5-(2-Aminoethoxy)-2-methoxyphenyl]-1H-pyrrolo[2,3-b]pyridin-4-yl}benzoic acid (52)

Compound **51** (81 mg, 0.12 mmol, 1.0 equiv) was dissolved in MeOH (1.5 mL, 0.08 M) and treated with potassium hydroxide (34 mg, 0.6 mmol, 5.0 equiv.) and heated to reflux for 18 hours. The crude mixture was then concentrated *in vacuo* and then treated with TFA (2.5 mL, 0.05 M). After two hours, the solution was concentrated *in vacuo* and then co-evaporated with MeOH. The resulting residue was taken forward to the next step without further purification.

4-(2-{2-Methoxy-5-[2-(prop-2-enamido)ethoxy]phenyl}-1H-pyrrolo[2,3-b]pyridin-4-yl)benzoic acid (53)

Compound **52** (12 mg, 0.03 mmol, 1.0 equiv.) was dissolved in anhydrous DMF (0.5 mL, 0.06 M) and was treated with triethylamine (30 µL, 0.18 mmol, 6.0 equiv.) and acryloyl chloride (7.5 µL, 0.09 mmol, 3.0 equiv.) and stirred at room temperature. After 1 hour, the reaction was re-subjected to the conditions above. After a further hour of stirring at room temperature, the solution was quenched with H_2O and the crude reaction mixture was then purified by reverse phase HPLC (20-60% MeCN 0.1% TFA in H_2O) to yield yellow solid **53** (4.1 mg, 30% yield); **^{1}H NMR (400 MHz, DMSO)** δ 12.06 (s, 1H), 8.39 (t, *J* = 5.6 Hz, 1H), 8.14 (d, *J* = 8.4 Hz, 2H), 7.94 (d, *J* = 8.4 Hz, 2H), 7.54 (d, *J* = 3.0 Hz, 1H), 7.27 (d, *J* = 4.8 Hz, 1H), 7.23 (s, 1H), 7.10 (d, *J* = 9.0 Hz, 1H), 6.96 (dd, *J* = 9.0, 3.0 Hz, 1H), 6.29 (dd, *J* = 17.1, 10.1 Hz, 1H), 6.12 (dd, *J* = 17.1, 2.3 Hz, 1H), 5.60 (dd, *J* = 10.1, 2.3 Hz, 1H), 4.09 (t, *J* = 5.7 Hz, 2H), 3.86 (s, 3H), 3.54 (q, *J* = 5.7 Hz, 2H); **^{13}C NMR (101 MHz, DMSO)** δ 167.0, 164.9, 152.4, 151.0, 149.3, 142.9, 142.6, 138.9, 136.1, 131.6, 130.5, 130.0, 128.5, 125.3, 120.3, 118.3, 115.7, 114.7, 113.8, 113.3, 99.9, 66.9, 56.1, 38.4; **HRMS** m/z calcd for $C_{26}H_{23}N_3O_5$ [M+H]+ calc for 458.1710 found 458.1718; **Retention Time** (min) 24.95 (5-95% ACN 0.1% TFA in H_2O 0.1% over 50 minutes), 99% purity.

4-[2-(2-Methoxy-5-{2-[(2*E*)-4-methoxy-4-oxobut-2-enamido]ethoxy}phenyl)-1H-pyrrolo[2,3-b]pyridin-4-yl]benzoic acid (54)

A solution of monomethyl fumarate (13 mg, 0.14 mmol, 5.0 equiv.), DIPEA (46 µL, 0.27 mmol, 10.0 equiv.) and HATU (46 mg, 0.14 mmol, 5.0 equiv.) in DMF (540 µL, 0.25 M) was stirred at room temperature for 30 minutes. Compound **52** (11 mg, 0.027 mmol, 1.0 equiv.) was added and the solution was stirred overnight. The crude reaction mixture was then purified by reverse phase flash column chromatography on an Isolera one using a 30g C18 column (5-95% ACN 0.1% TFA in H_2O 0.1%) to yield a yellow solid (3.1 mg, 22% yield); **^{1}H NMR (400 MHz, DMSO)** δ 12.06 (d, *J* = 2.1 Hz, 1H), 8.84 (t, *J* = 5.6 Hz, 1H), 8.33 (d, *J* = 5.0 Hz, 1H), 8.14 (d, *J* = 8.5 Hz, 2H), 7.94 (d, *J* = 8.5

Hz, 2H), 7.54 (d, J = 2.9 Hz, 1H), 7.27 (d, J = 5.0 Hz, 1H), 7.22 (d, J = 2.1 Hz, 1H), 7.10 (d, J = 9.0 Hz, 1H), 7.09 (d, J = 15.5 Hz, 1H), 6.96 (dd, J = 9.0, 2.9 Hz, 1H), 6.62 (d, J = 15.5 Hz, 1H), 4.12 (t, J = 5.6 Hz, 2H), 3.86 (s, 3H), 3.72 (s, 3H), 3.58 (q, J = 5.6 Hz, 2H); **^{13}C NMR (101 MHz, DMSO)** δ 167.0, 165.5, 163.1, 152.3, 151.1, 149.3, 142.8, 142.6, 138.9, 137.5, 136.1, 130.5, 130.0, 128.4, 128.2, 120.3, 118.3, 115.7, 114.6, 113.9, 113.3, 99.9, 66.7, 56.1, 52.0, 38.8; HRMS m/z calcd for $C_{28}H_{25}N_3O_7$ [M+H]+ calc for 516.1765 found 516.1761**; Retention Time** (min) 27.60 (5-95% MeCN 0.1% TFA in H_2O 0.1% over 50 minutes, 254 nm), 95% purity.

Metabolic Stability

Stability in human serum was evaluated using HPLC. To an Eppendorf containing 15 µL compound (10 mM in DMSO) in a pre-heated block (37 ℃) was added 135 µL PBS buffer and 150 µL serum from human male AB plasma (USA origin, sterile-filtered, H4522) to initiate the reaction (0.5 mM compound and 5% DMSO end concentration). The reaction was incubated at 37 ℃. At each time point (2 minutes – 24 hours), 12.5 µL was removed and 87.5 µL of cold MeOH (-20 ℃, 2% TFA) was added to quench the reaction. Samples were centrifuged (1 minute, 4000 g), and 10 µL was then injected onto a Shimadzu HPLC to run over 15 minutes (5-95% ACN +0.1% TFA in H2O +0.1% TFA). Consumption of starting material was then monitored by UV (compound 4 was monitored by a wavelength of 254 nm. The natural log of the peak area was plotted against time to yield a straight line. The gradient of this line was taken as the pseudo-first order rate constant, and $t_{1/2}$ was obtained by dividing -ln(2) by this value.

Time (mins)	2	57	12	27	28	14
0			15.41061	14.9625	15.09448	14.34307
2			14.97065	14.84284	14.74307	14.42218
5	12.87952	13.42418	14.98097	14.84519	14.90545	14.57064
10	13.02155	13.44746	15.14292	14.93732	15.00214	14.52707
20	12.98281	13.47232				
25			15.29072	14.74061	14.92427	14.48514
40	12.87515	13.55012	15.26138	14.89823	14.97073	14.40321
60			15.13199	14.87072	15.00639	14.44274
120	12.85074	12.55918	15.1073	14.91846	14.97503	14.47788
240	12.89964	12.13231	14.9922	14.89822	15.01547	14.36858
1440	11.73031	7.78904	14.57247	14.92503	14.85133	13.05222
3120			13.49875	14.56546	14.66422	11.70144

Table S1: Human serum stability data. 0.5 mM compound in 1:1 PBS:Serum (5% DMSO) at 37 ℃. Values given as Ln(peak area) for each compound.

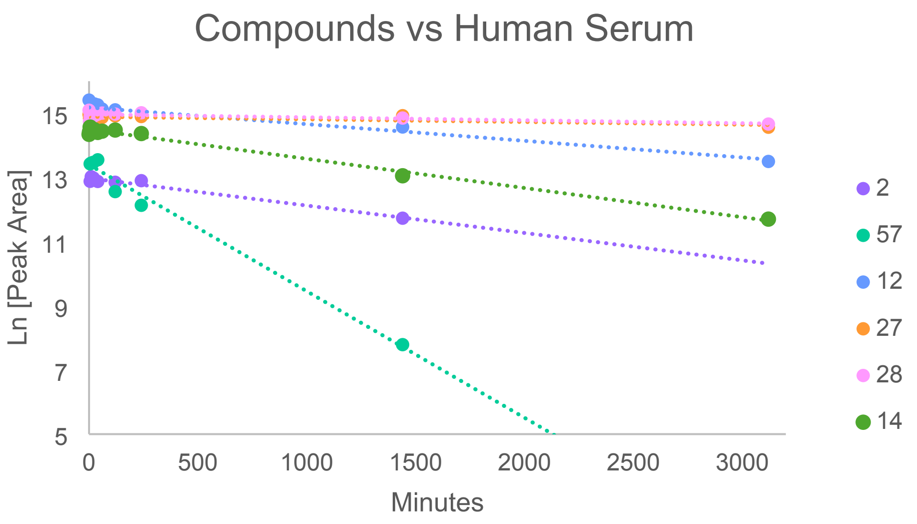

Figure S1: Plotted human serum stability data.

Glutathione stability was determined using HPLC. To an Eppendorf containing 125 µL compound (10 mM in DMSO) in a pre-heated block (37 °C) was added 1125 µL GSH (11.1 mM in 5 mM phosphate buffer, pH 7.5) to initiate the reaction. The reaction was incubated at 37 °C, and 100µL aliquots were removed at time points (2 minutes – 72h). 900µL cold MeOH (-20 °C, 0.1% formic acid) was added to each aliquot to quench the reaction. 40 µL was then injected onto a Shimadzu HPLC to run over 15 minutes (5-95% ACN +0.1% TFA in H2O +0.1% TFA). Consumption of starting material was then monitored by UV (214 nm) and the natural log of the peak area was plotted against time to yield a straight line. The gradient of this line was taken as the pseudo-first order rate constant, and $t_{1/2}$ was obtained by dividing -ln(2) by this value.

Time (mins)	12	27	28	14	15	10	2	57
2	15.35121	15.0934	15.35322	14.91992	15.07695	15.13916	13.64359	
5	15.42623	15.15033	15.52369	14.97779	15.13231	15.14423	13.55396	
10	15.37341	15.09928	15.49448	14.95547	15.22027	15.11663	13.15263	15.18
15	15.28205	14.76412	15.31271	14.81863		15.05106	12.82032	15.2666
20							12.42066	15.28915
25							11.9431	
30	14.88117	14.23564	15.07311	14.65063	15.01626	15.05074	11.62591	15.13755
40							11.00553	15.087
50							10.07803	
60	13.1283	12.84244		14.30361	15.1676	14.92074	8.887929	15.26
70							7.628031	
120	11.13049	11.6843	13.21755	13.79061	14.91745	14.2002		15.17873
180			12.53159	13.27479	14.81799	14.90708		
240				13.24241	14.2105	14.8692		15.20553
360				12.98667	13.87599	14.87117		15.21695
1440				12.11845		14.76021		
1500								15.2951

Table S2: Glutathione Stability Data. 1 mM compound and 10 mM GSH in PB buffer, at 37 °C. Values given as Ln(peak area) for each compound.

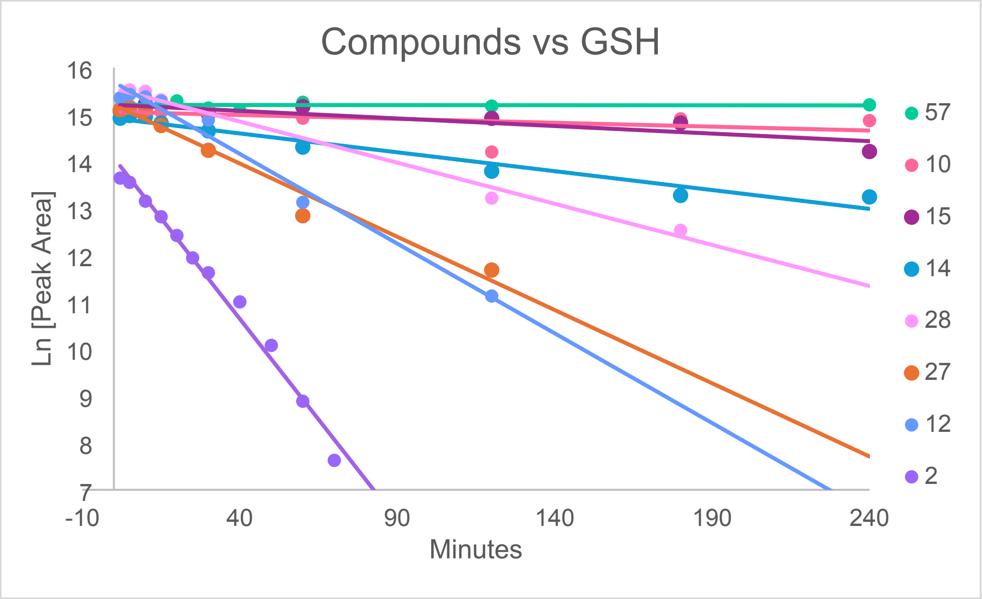

Figure S2: Plotted glutathione stability data.

Stability in hepatocytes were carried out by Pharmaron Ltd. For hepatocyte stability, 198 µL of hepatocytes and boiled hepatocytes in William's E Medium supplemented with GlutaMAX were added to a 96-well non-coated plate, and incubated at 37°C for 10 minutes. 2 µL of 100 µM test compound or positive control was added start the reaction, followed by further incubation. Well contents were transferred in 25 µL aliquots at time points of 0.5, 15, 30, 60, 90 and 120 minutes. The aliquots were then mixed with 6 volumes (150 µL) of acetonitrile containing with internal standard, IS (100 nM alprazolam, 200 nM caffeine and 100 nM tolbutamide) to terminate the reaction. Samples were vortexed for 5 minutes and centrifuged for 45 minutes at 3,220 g. 100 µL of the supernatant was diluted in 100 µL ultra-pure water, and the mixture was used for LC/MS/MS analysis. All incubations were performed in duplicate. Peak areas were determined from extracted ion chromatograms. The slope value, k, was determined by linear regression of the natural logarithm of the remaining percentage of the parent drug vs. incubation time curve.

Compound ID	Species	Remaining Percentage (%)					
		0.5 min	15 min	30 min	60 min	90 min	120 min
Verapamil	Mouse	100.00	11.56	2.12	0.29	BLOD	BLOD
SB5-171 (**14**)	Mouse	100.00	70.68	46.20	23.93	13.64	10.26
SB6-10 (**28**)	Mouse	100.00	14.44	1.62	BLOD	BLOD	BLOD

Table S3: Remaining percentage of compounds **14** and **28** when incubated at 37 °C with mouse liver hepatocytes. Reference compound verapamil also given in table.

NMR Spectra for Novel Compounds

^{1}H Spectra (DMSO-d6) for compound 8

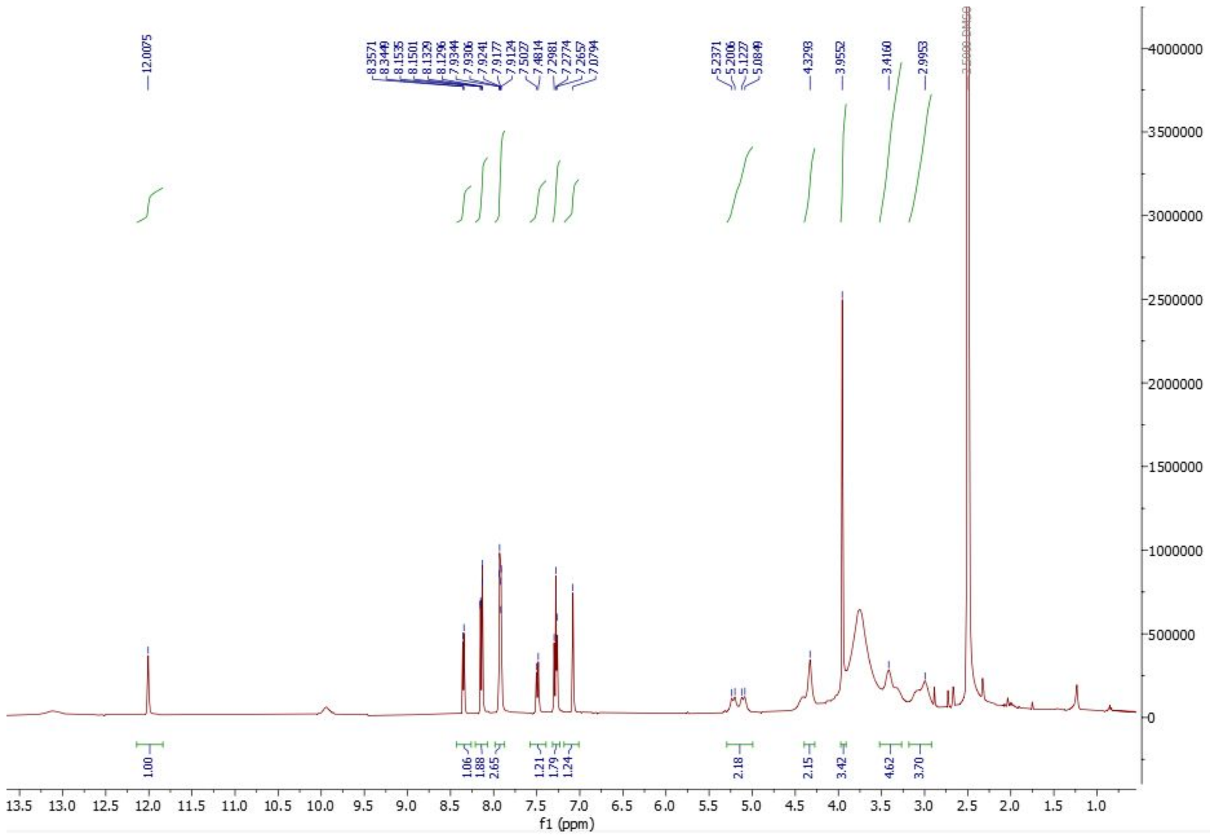

[1]H Spectra (DMSO-d6) for compound 9

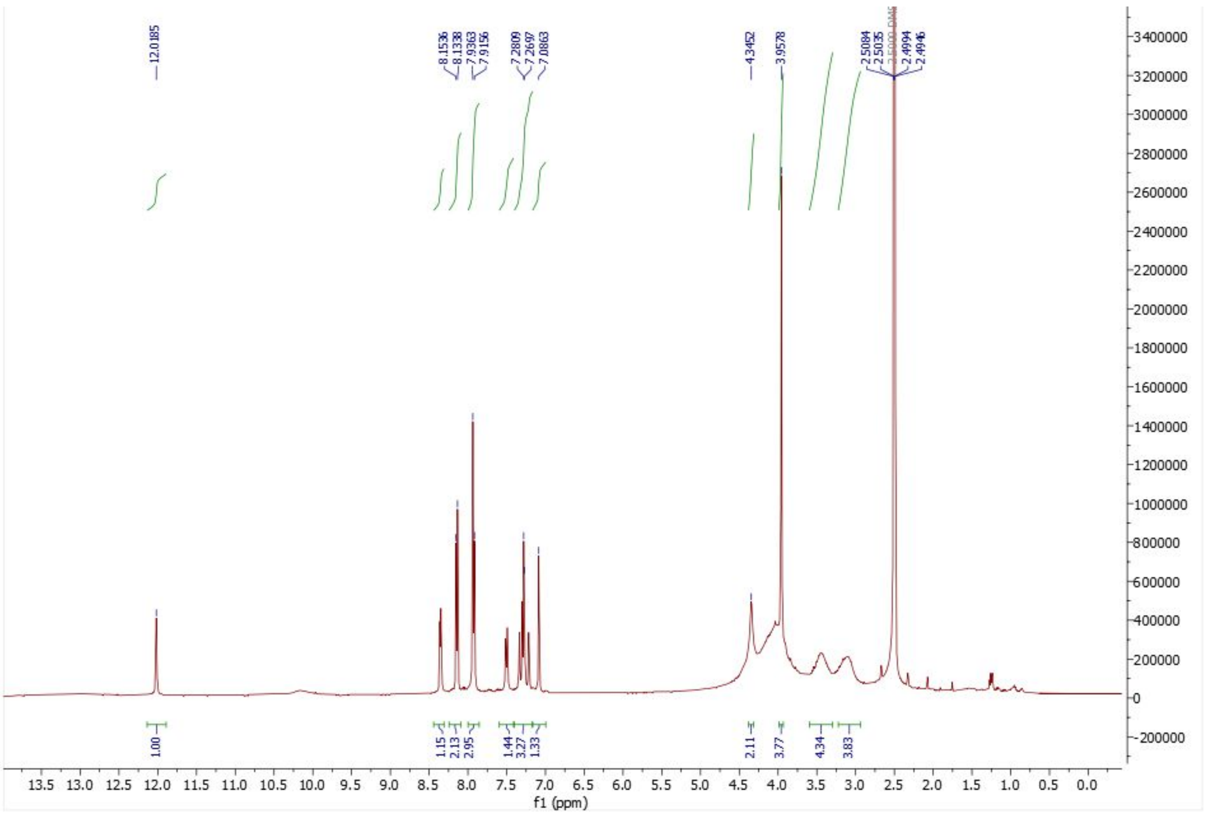

^{1}H and ^{13}C Spectra (DMSO-d6) for compound 10

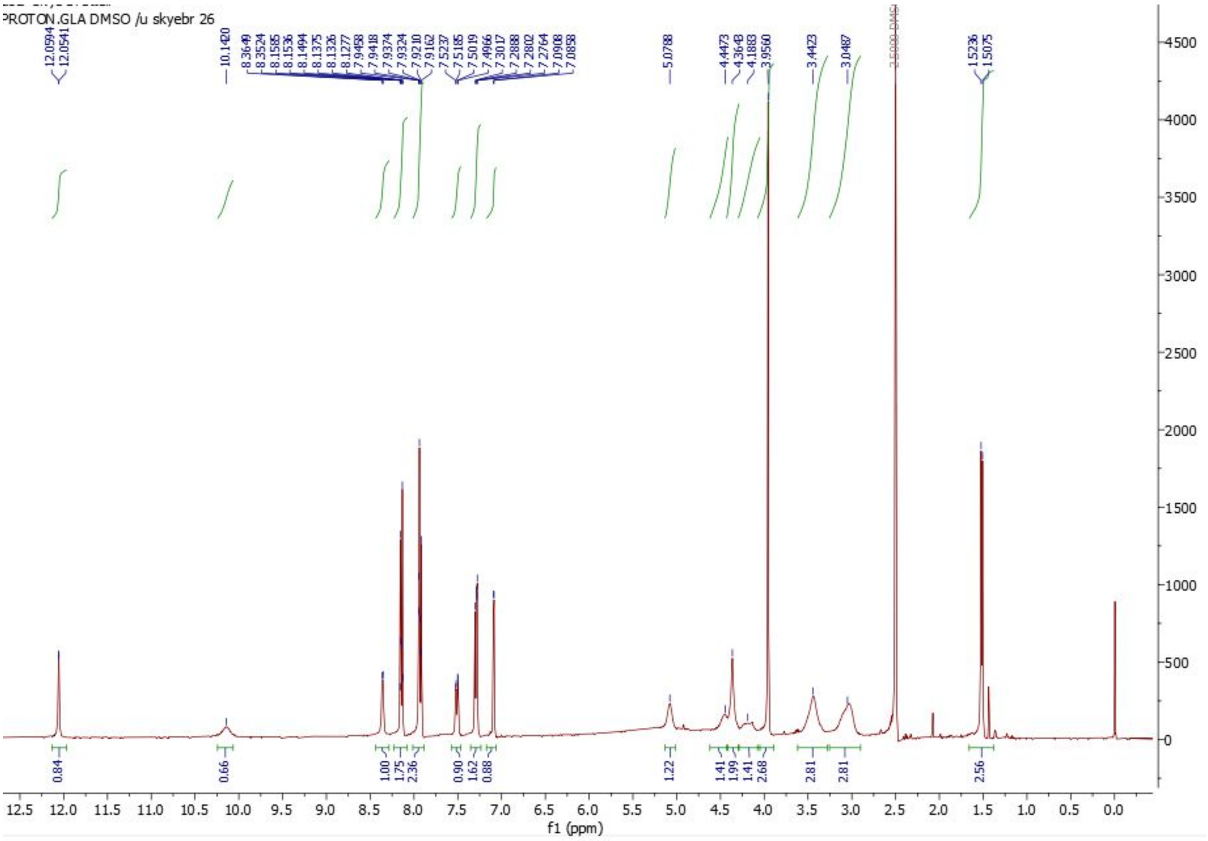

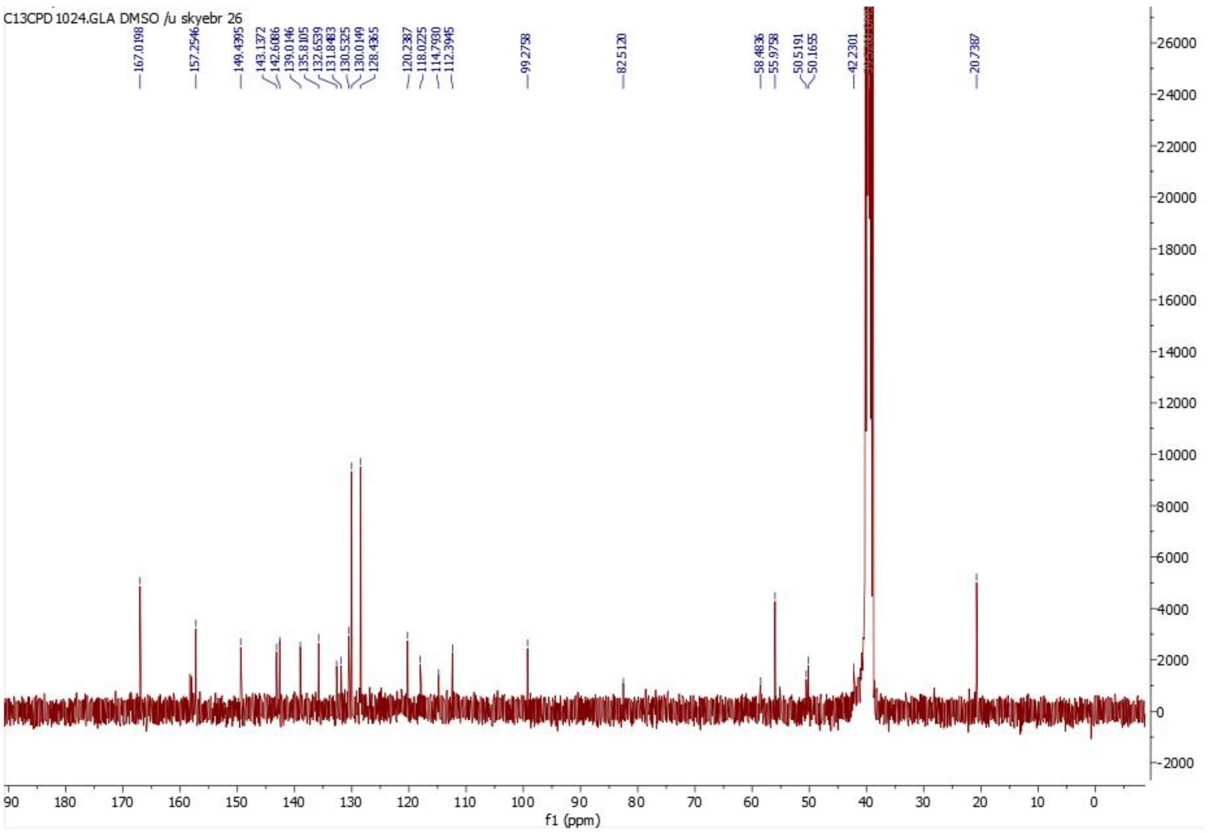

^{1}H and [13]C Spectra (DMSO-d6) for compound 12

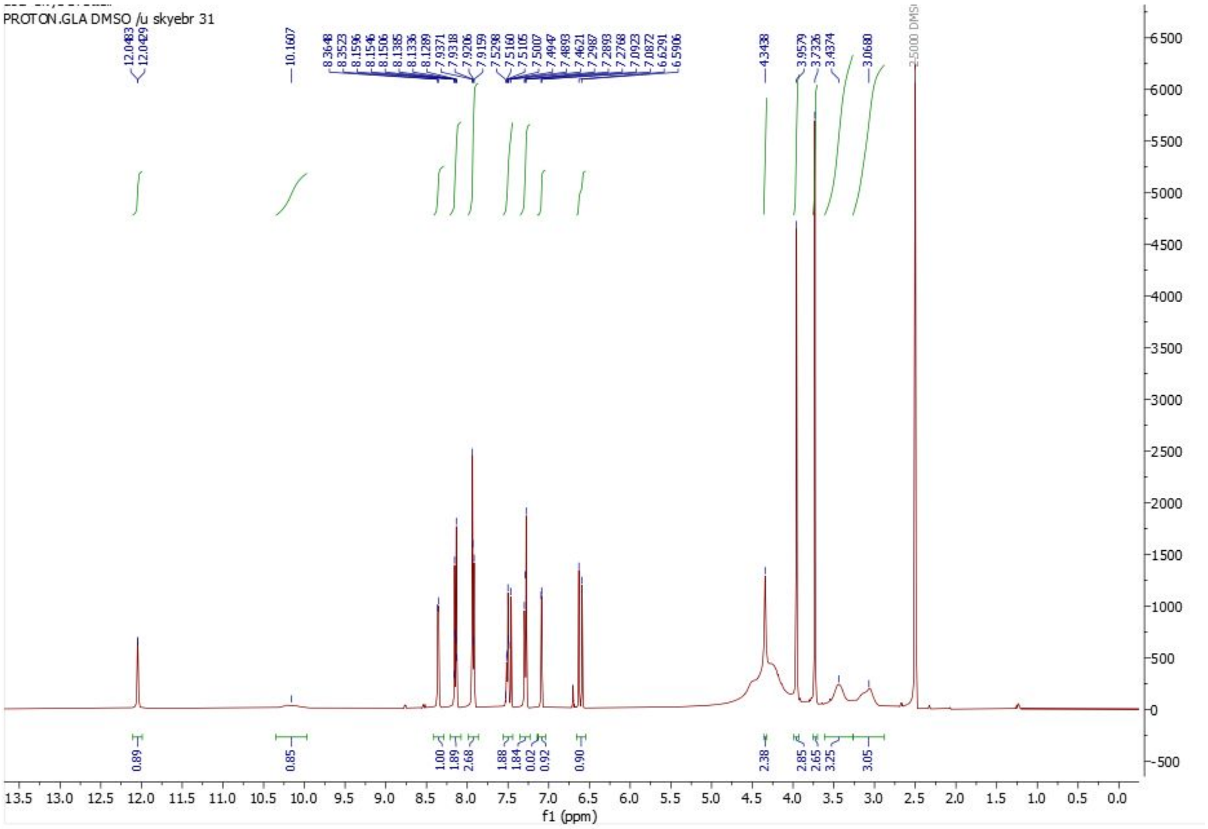

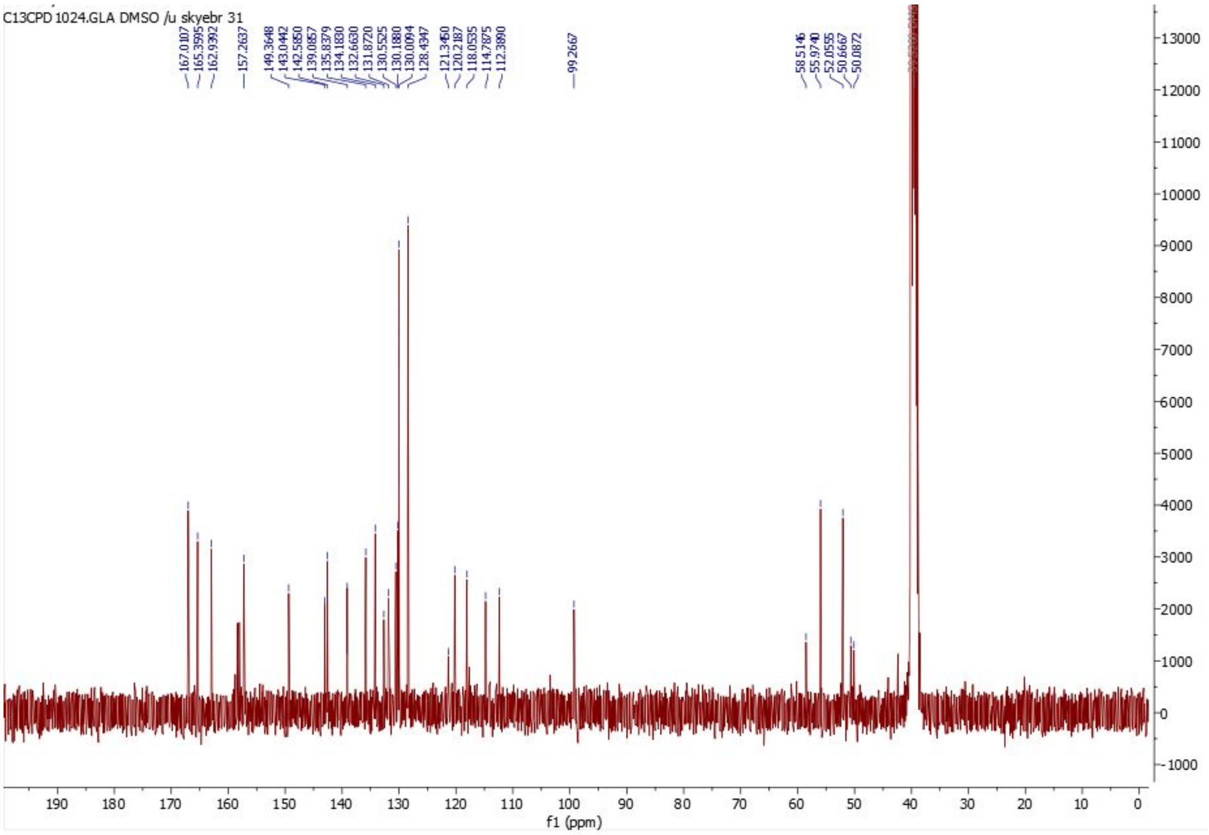

^{1}H and [13]C Spectra (DMSO-d6) for compound 27

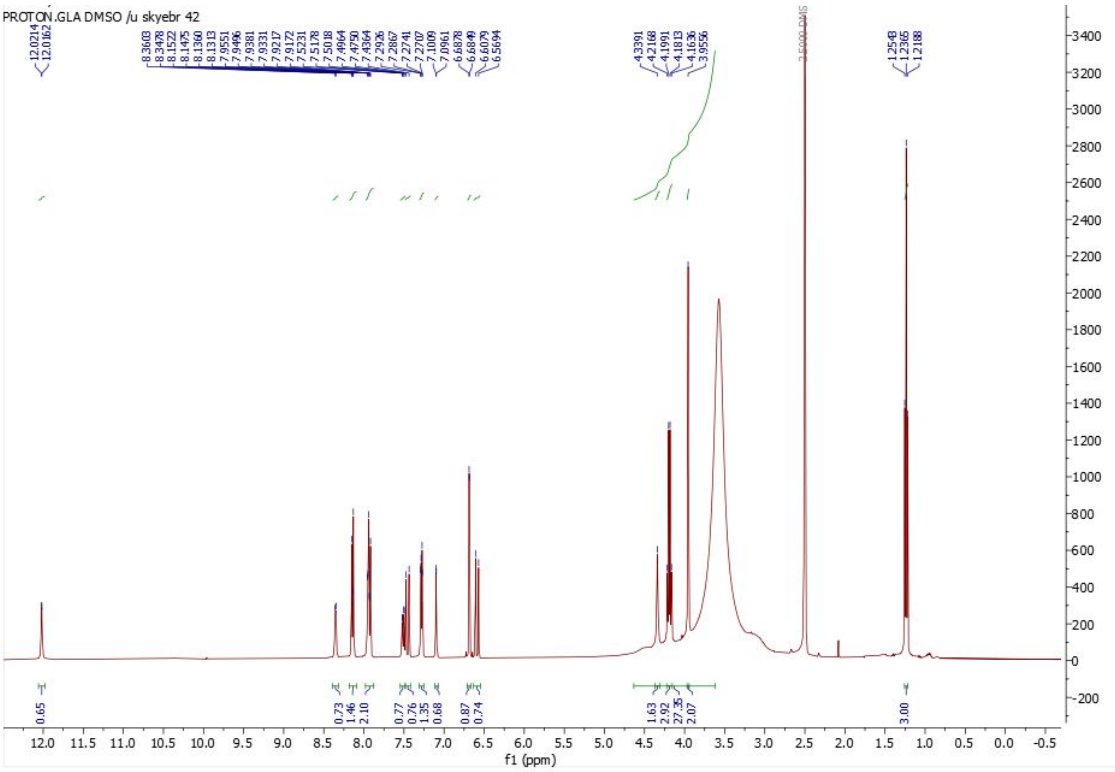

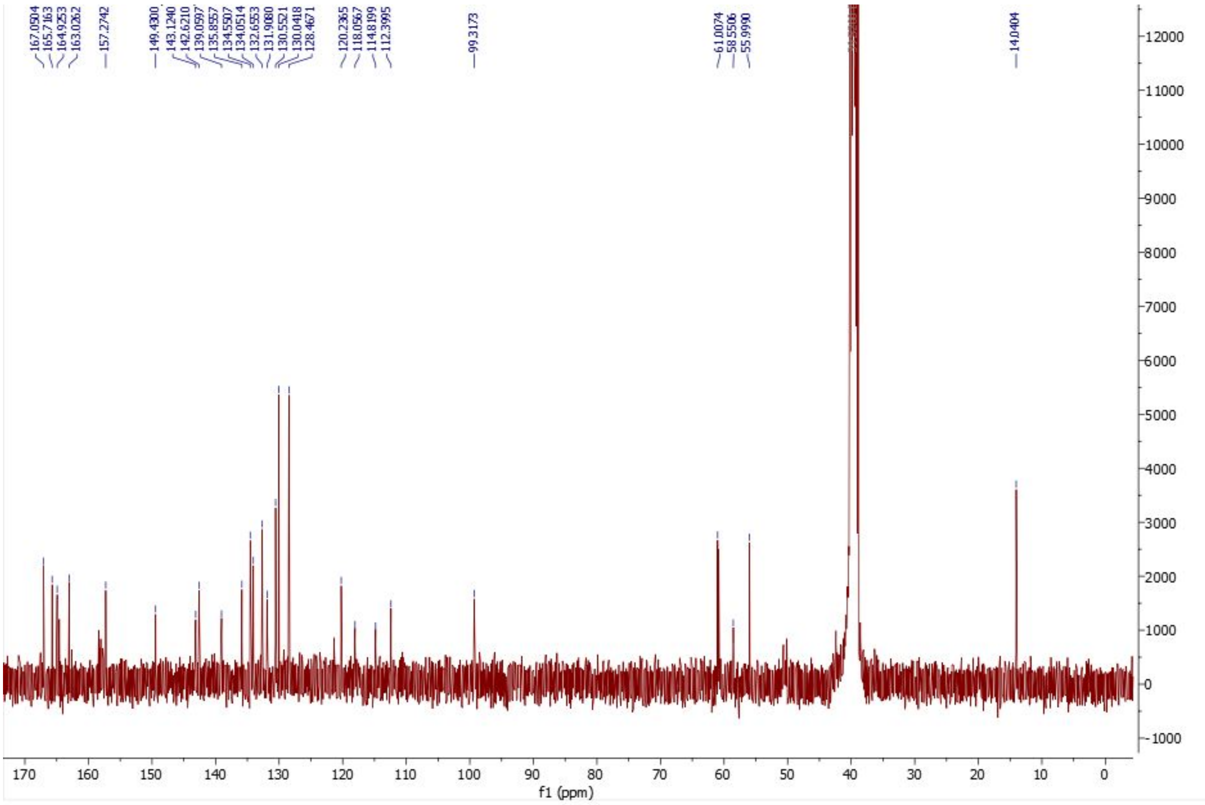

^1H and ^{13}C Spectra (DMSO-d6) for compound 28

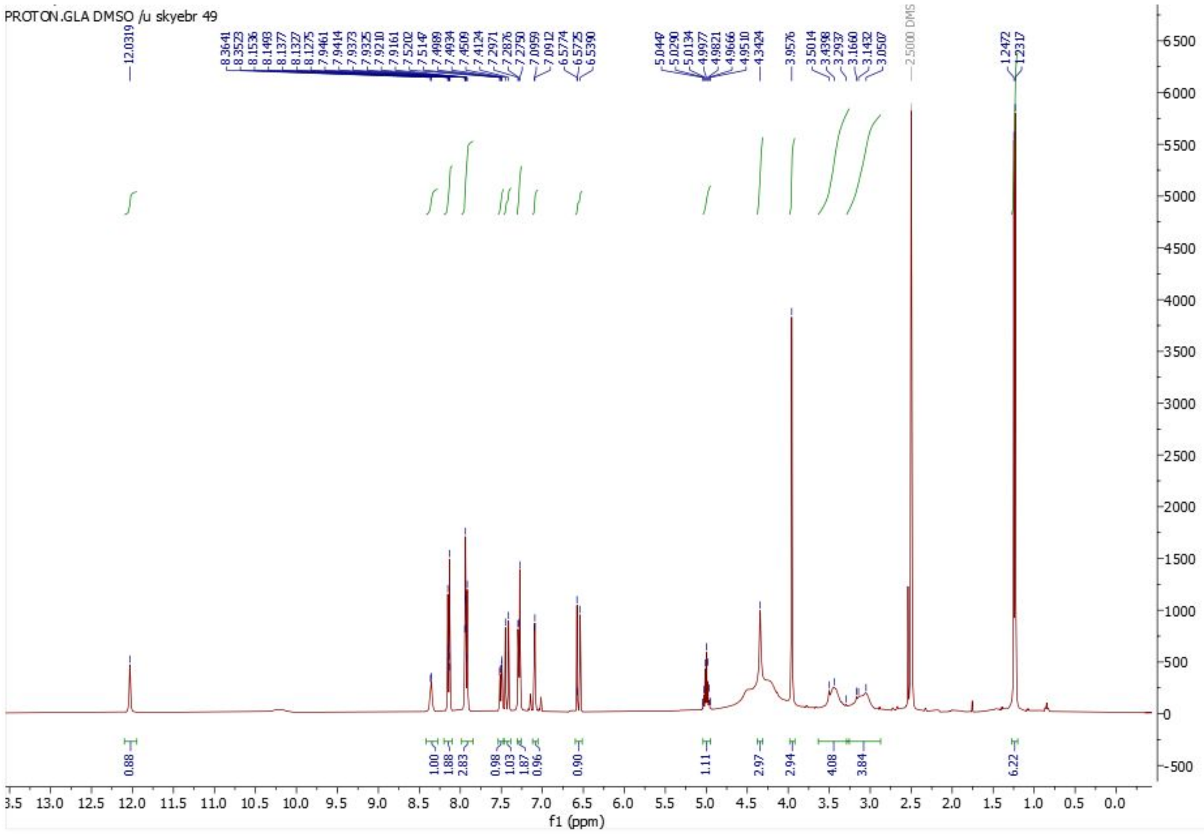

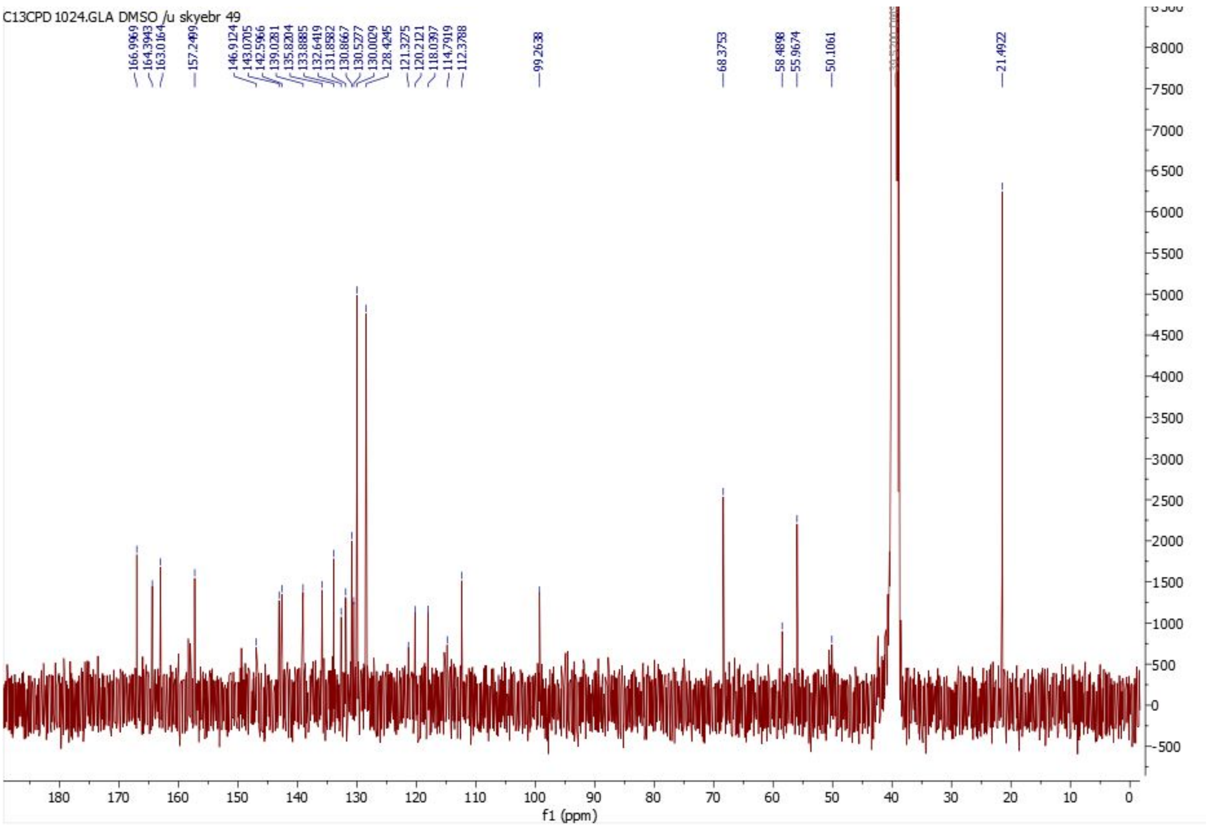

^{1}H and [13]C Spectra (DMSO-d6) for compound 29

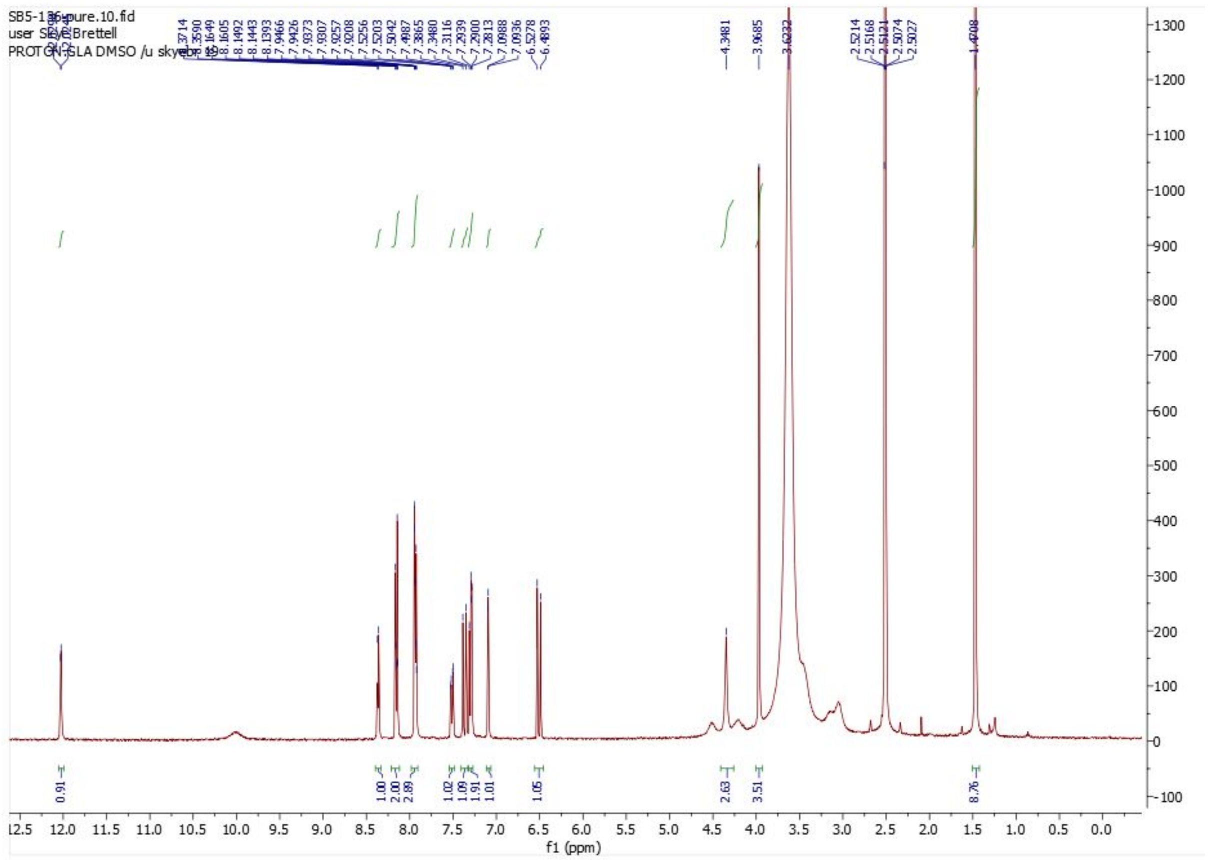

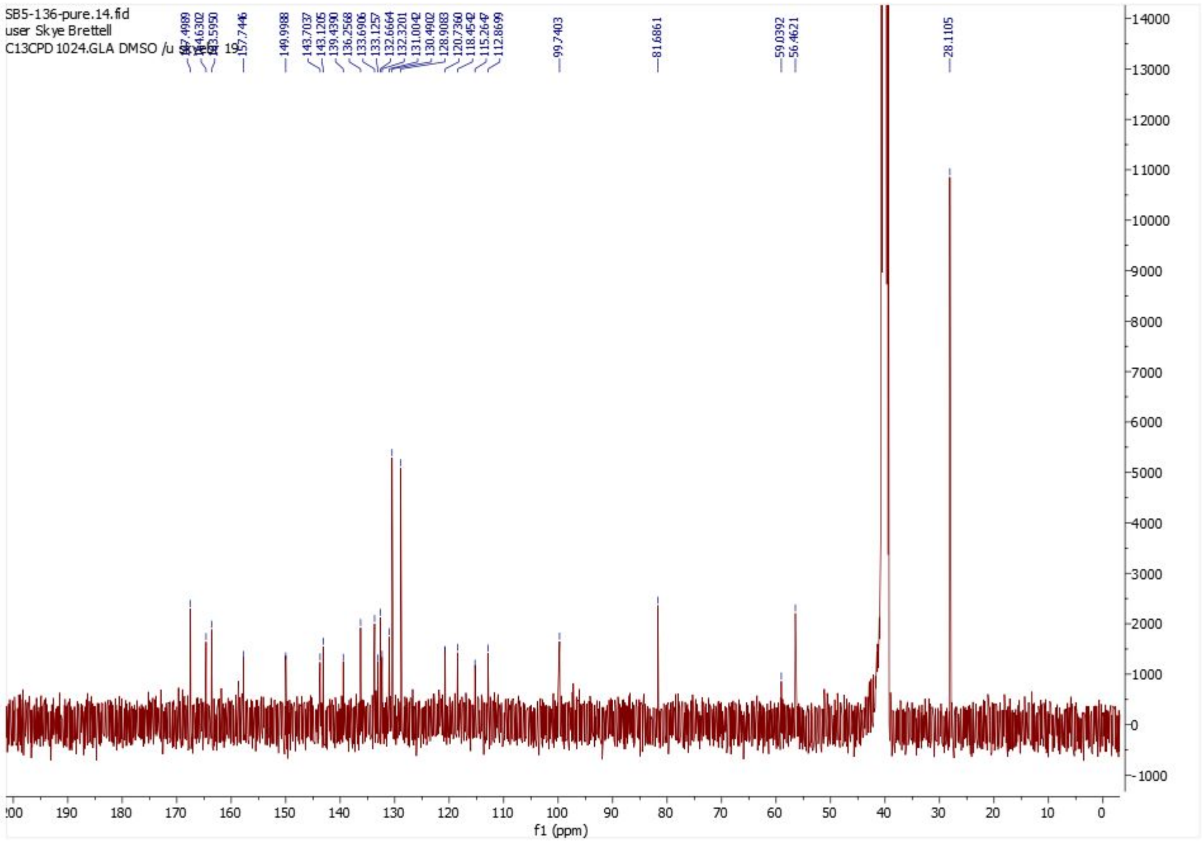

^{1}H and ^{13}C Spectra (DMSO-d6) for compound 13

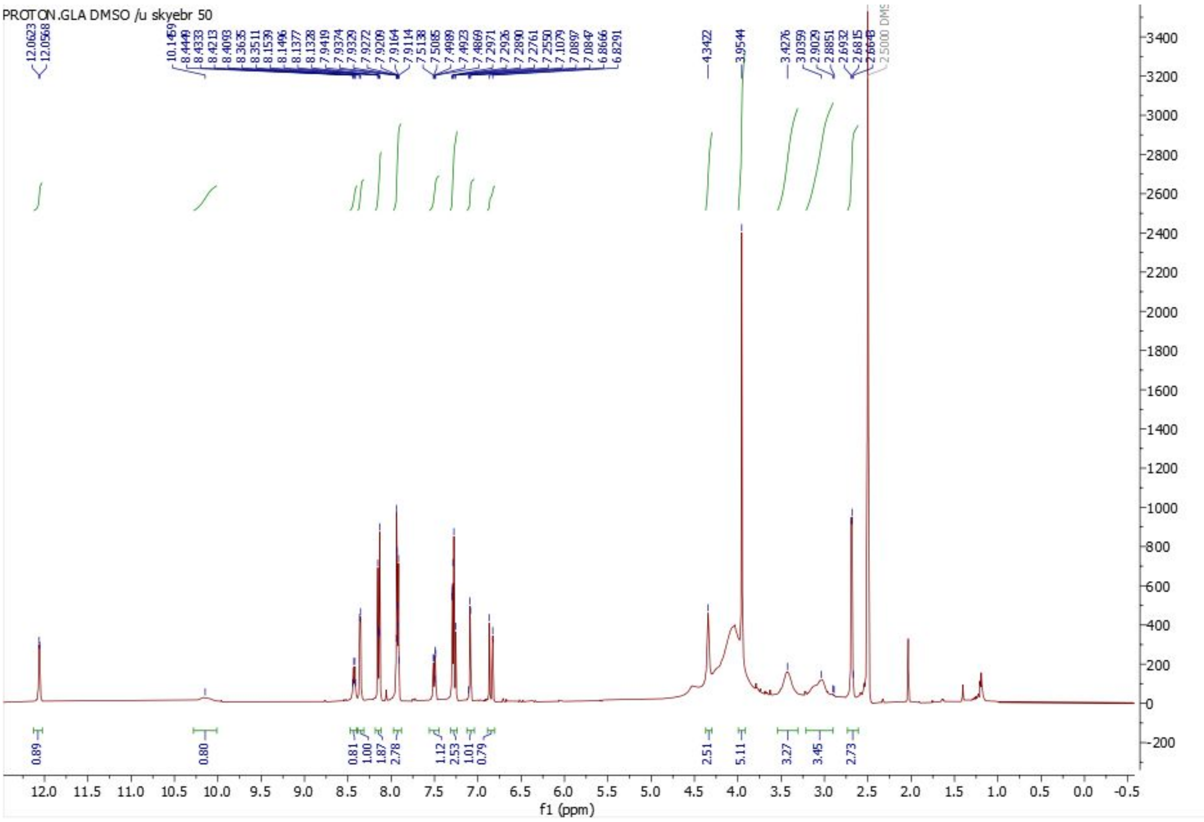

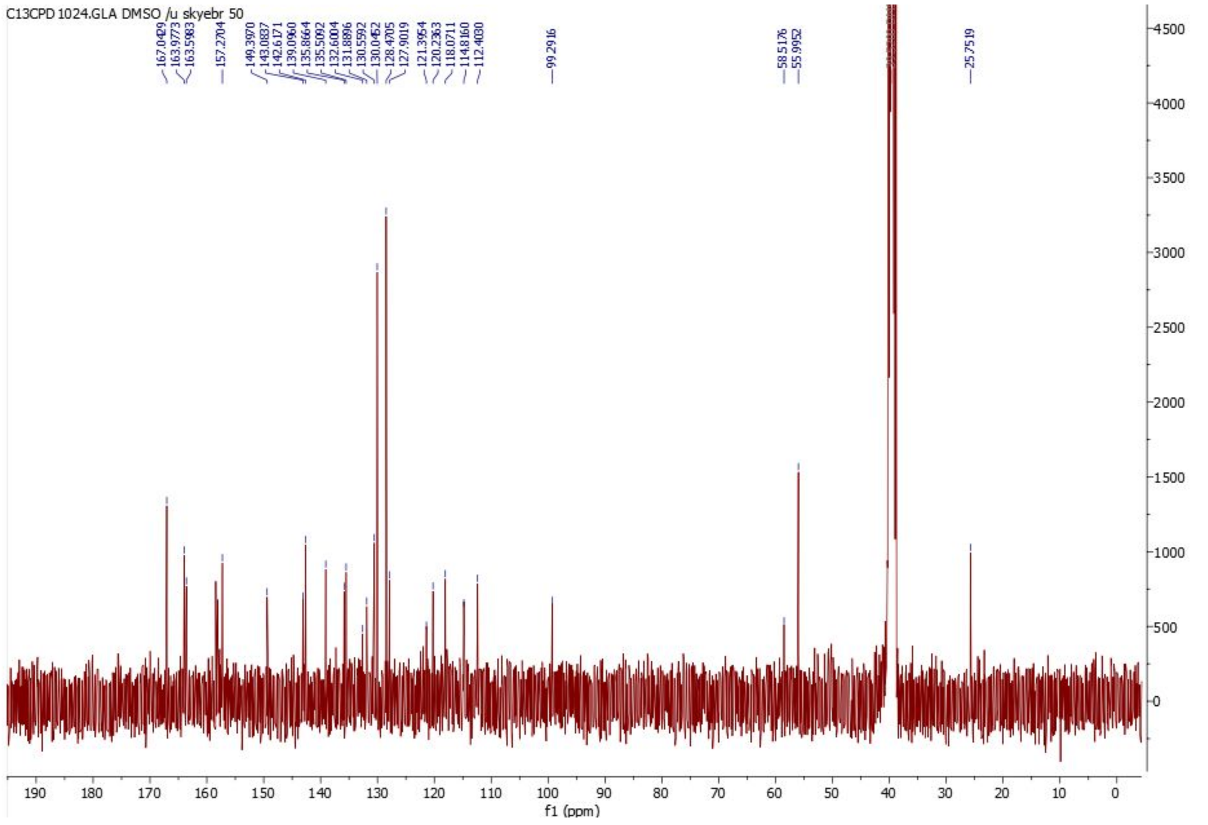

^{1}H and [13]C Spectra ($CDCl_3$) for compound S1

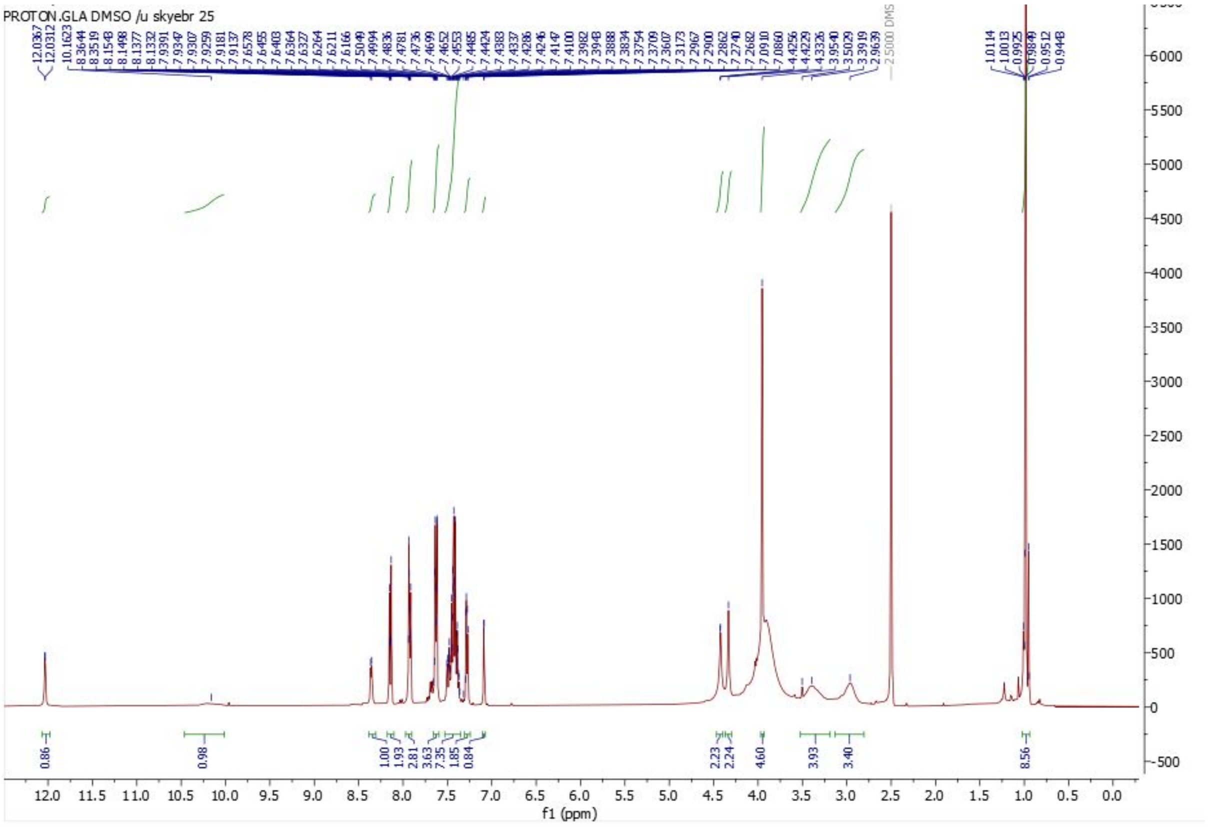

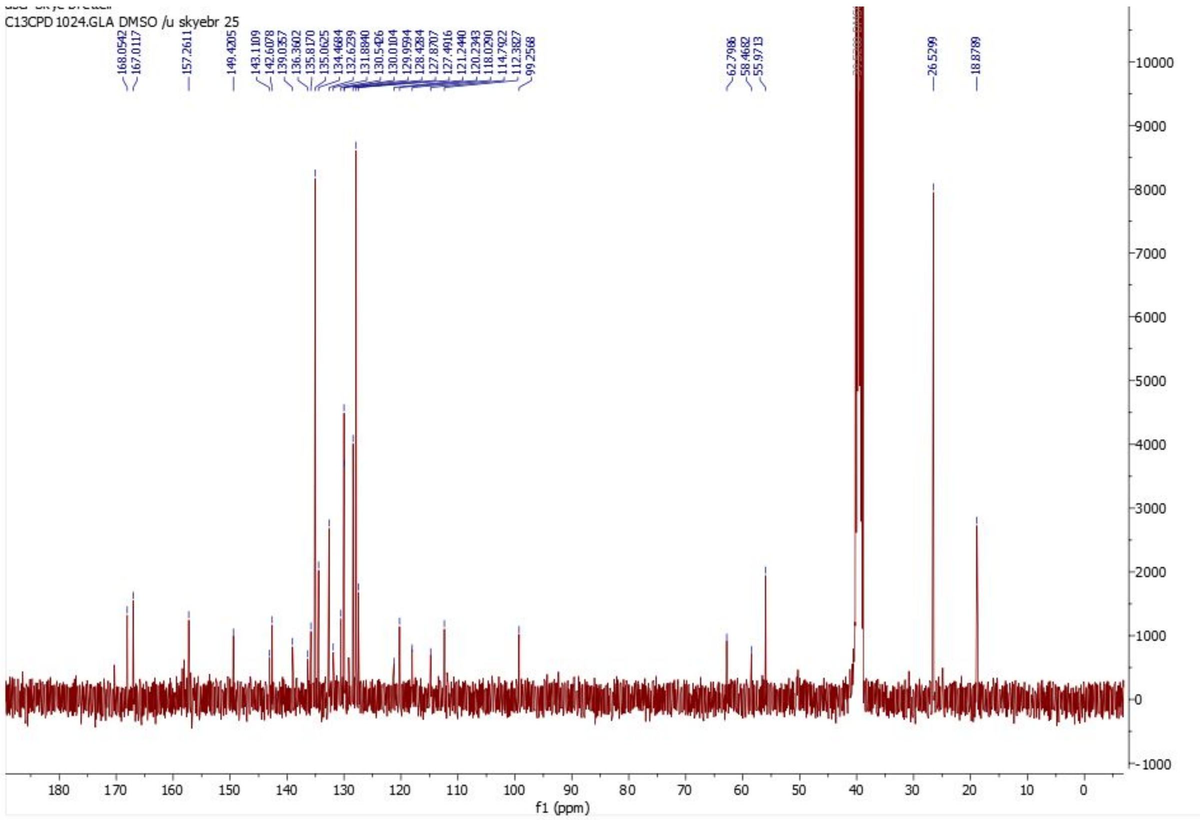

^{1}H and [13]C Spectra (DMSO-d6) for compound 14

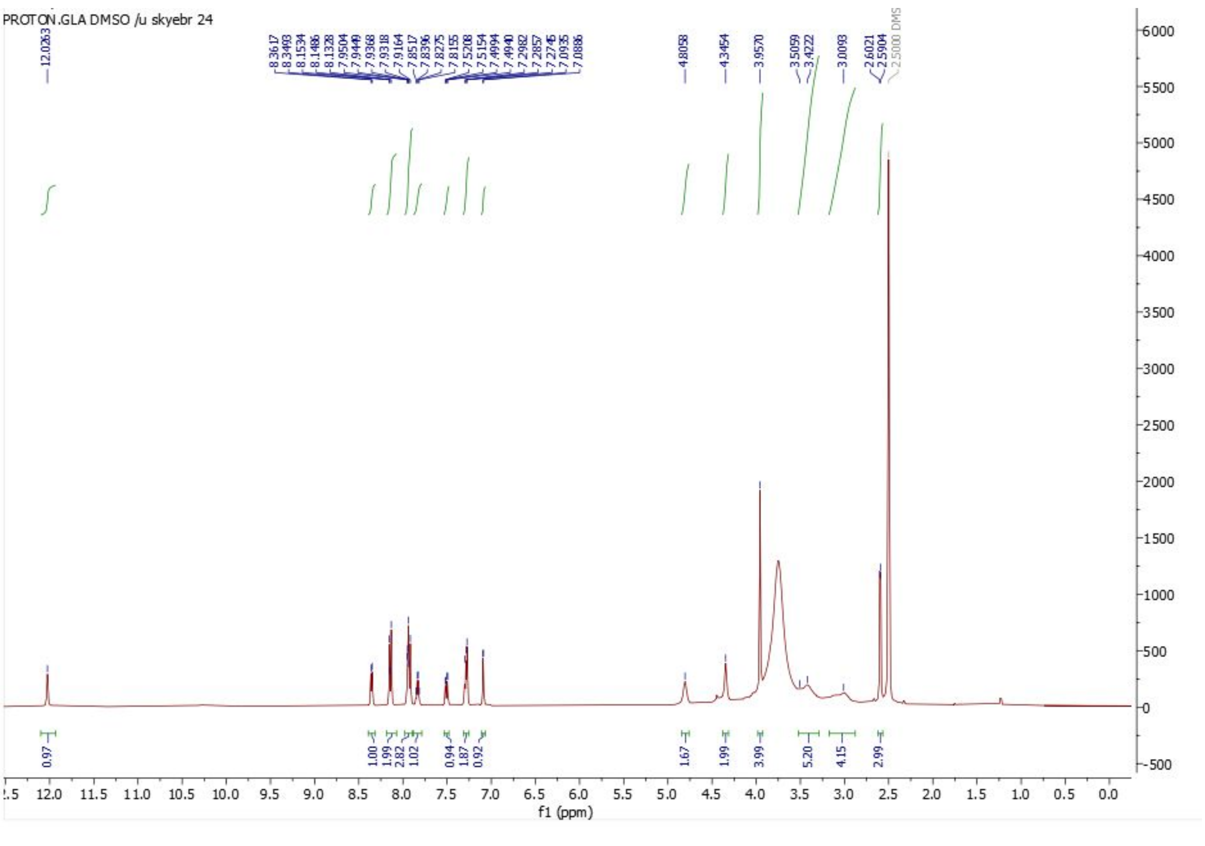

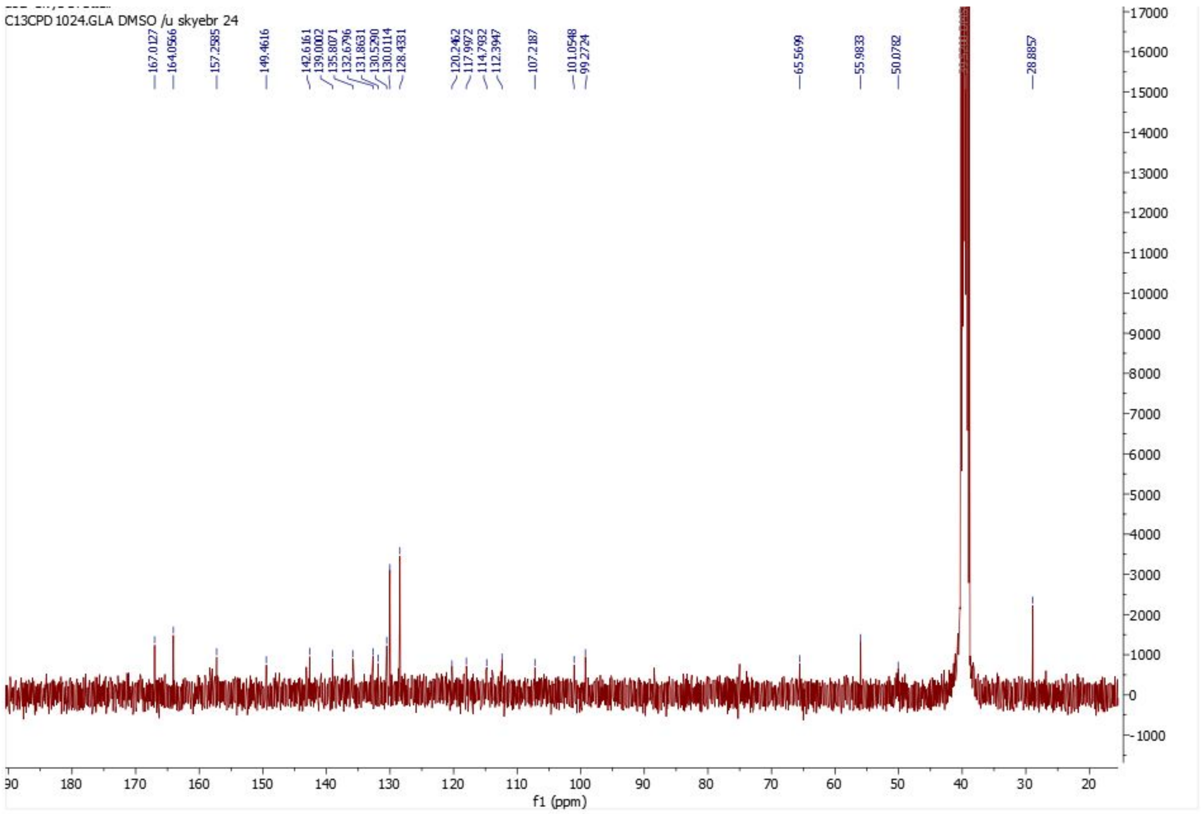

^{1}H and [13]C Spectra (DMSO-d6) for compound 15

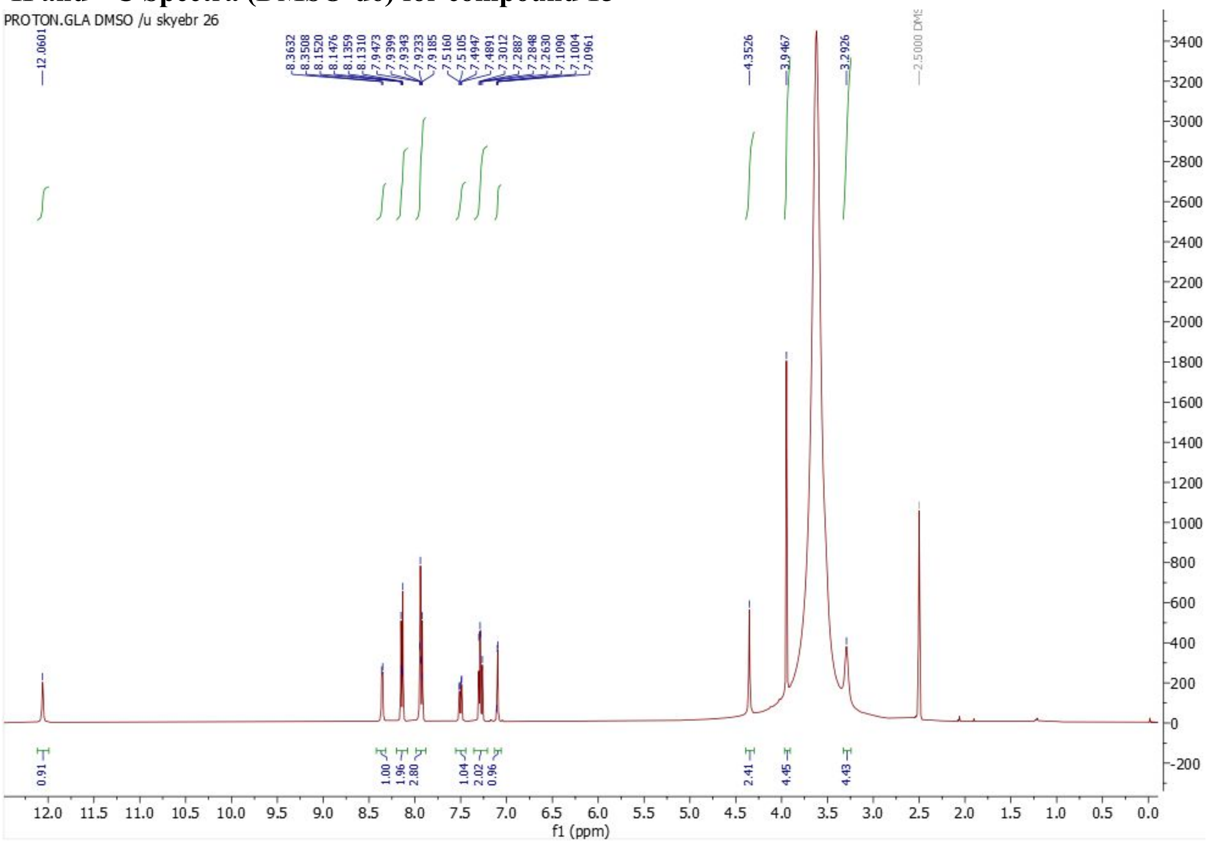

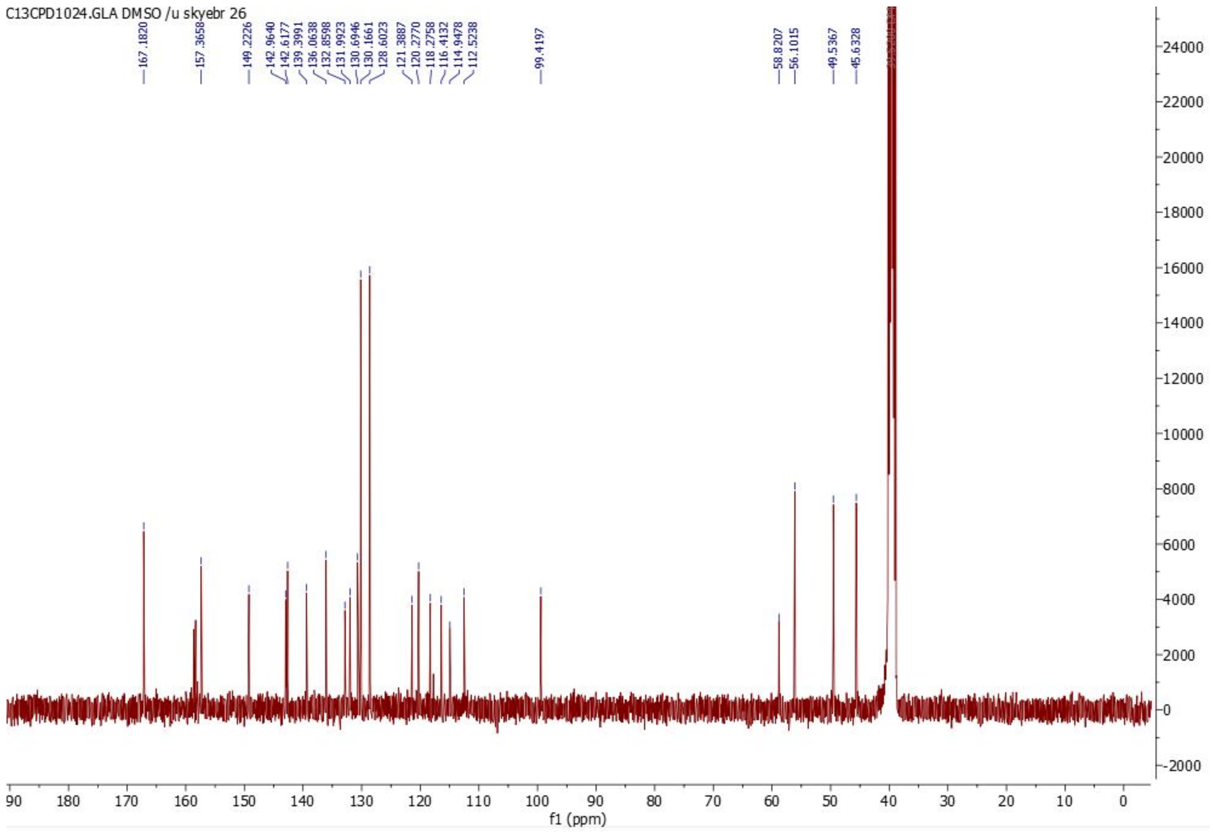

^1H and ^{13}C Spectra (DMSO-d6) for compound 16

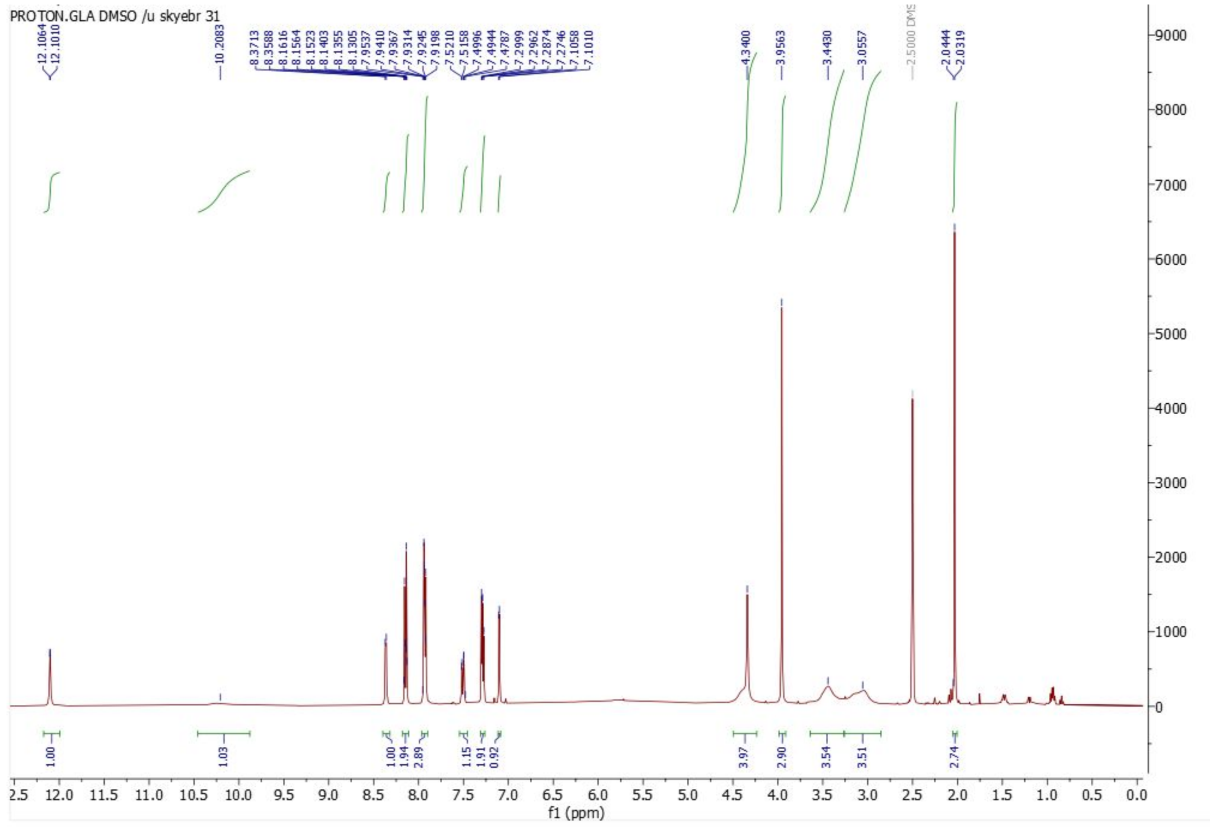

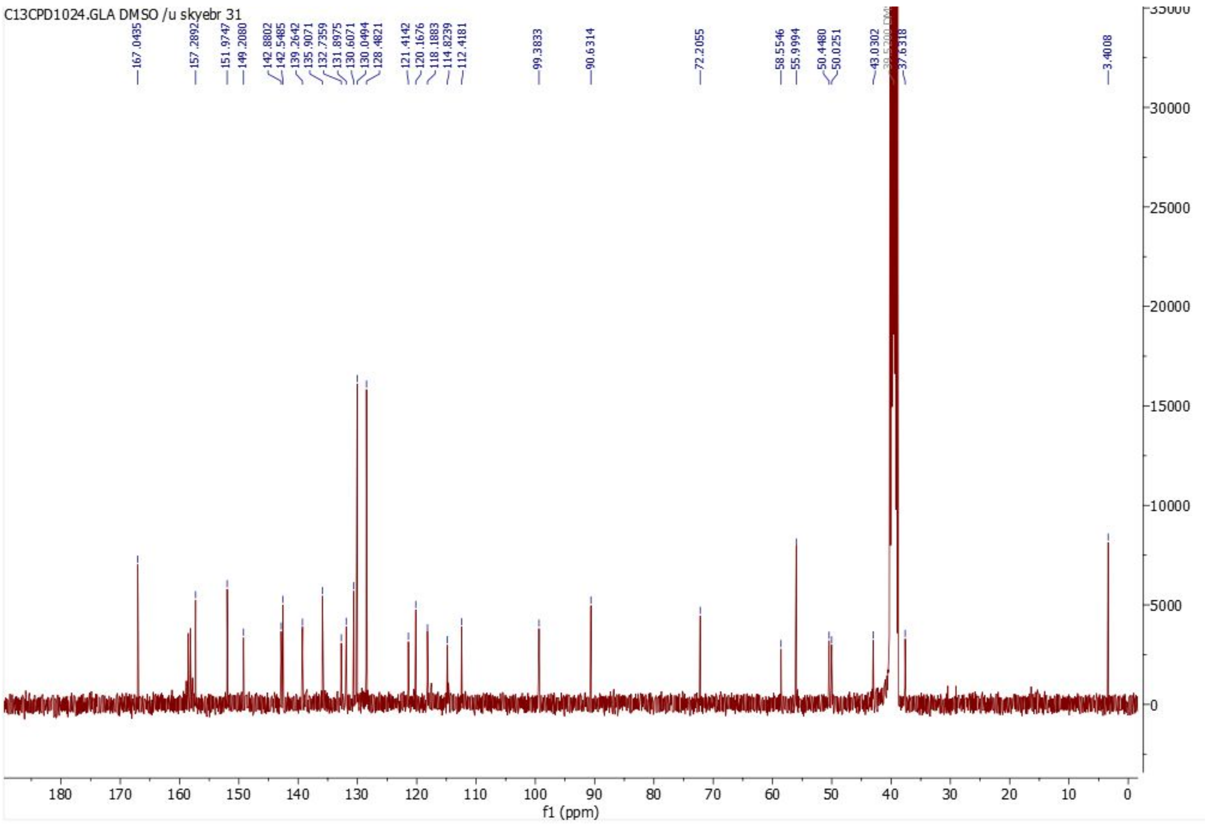

^{1}H and [13]C Spectra ($CDCl_3$) for compound 32

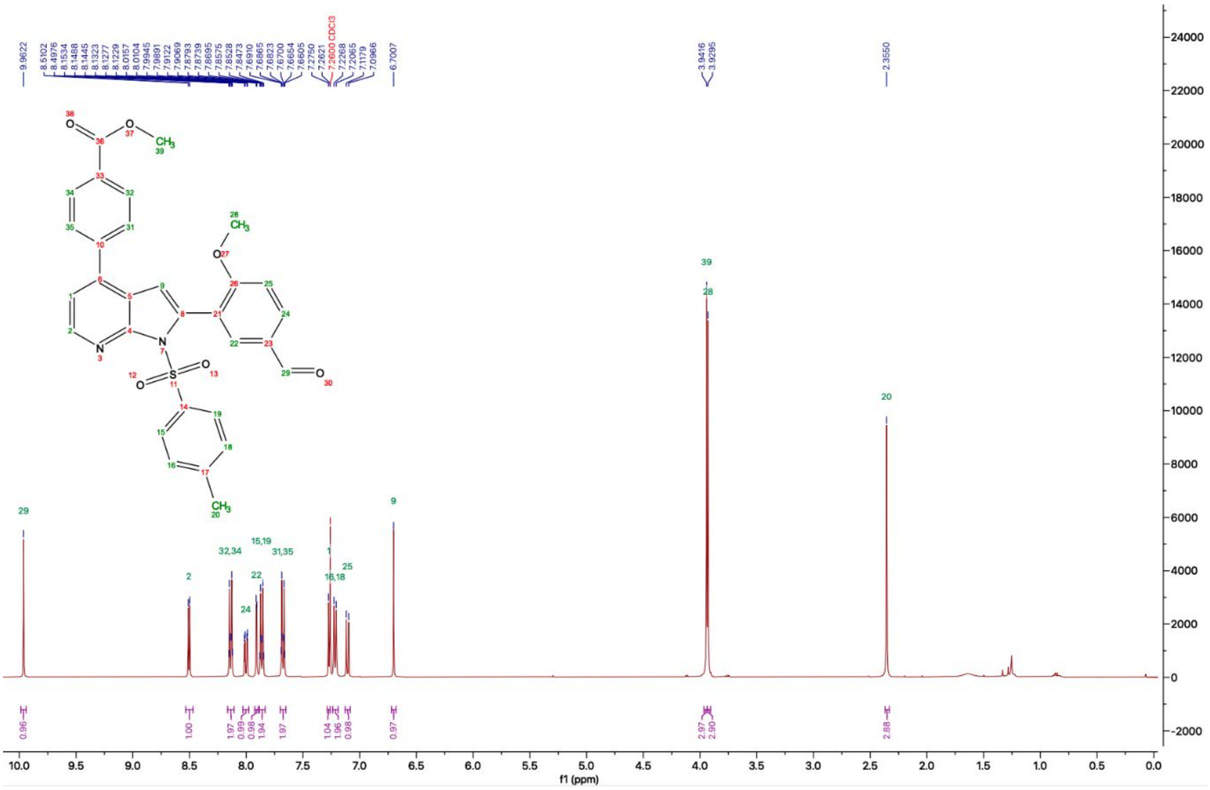

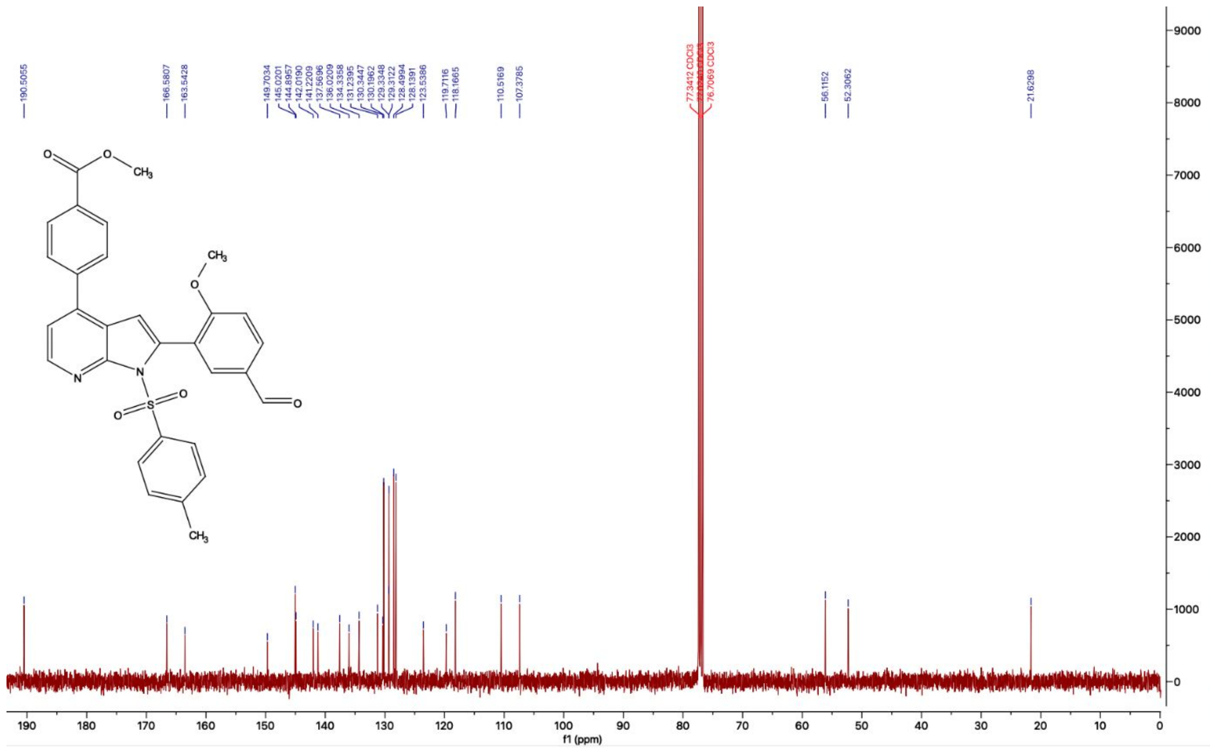

^{1}H and ^{13}C Spectra ($CDCl_3$) for compound 33

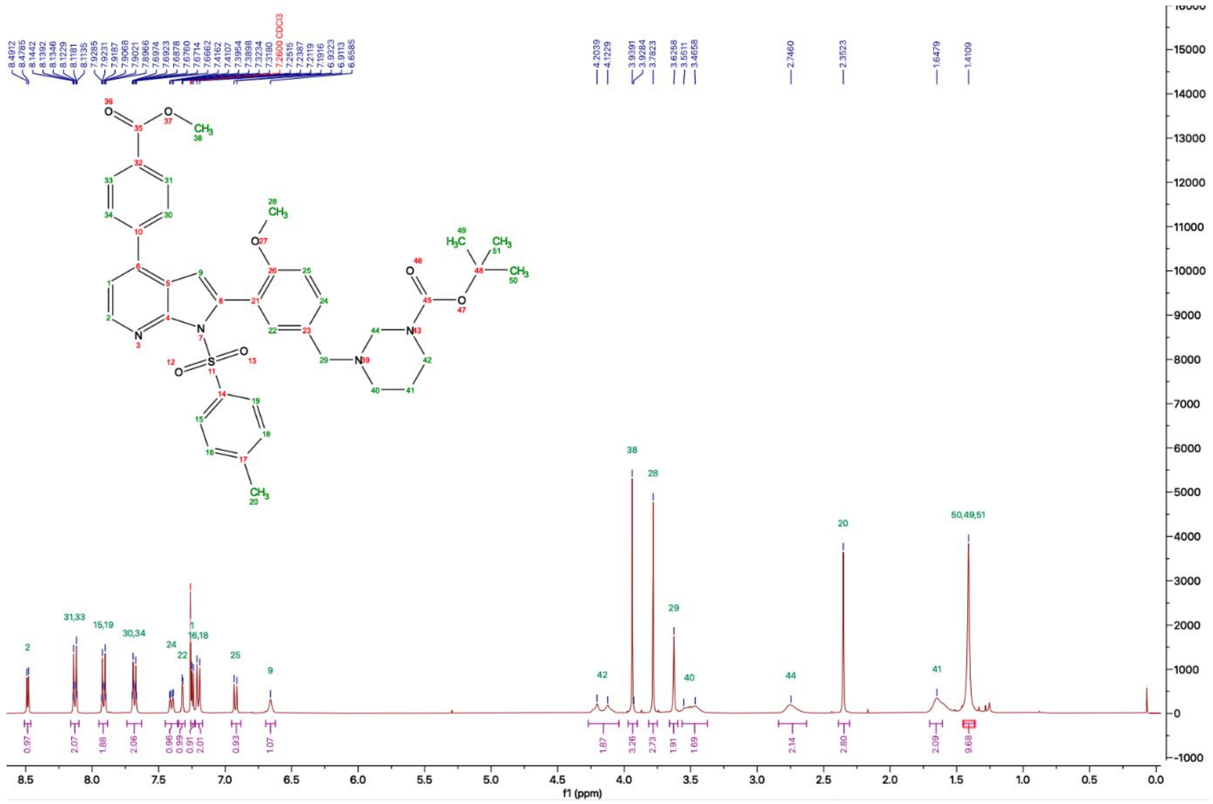

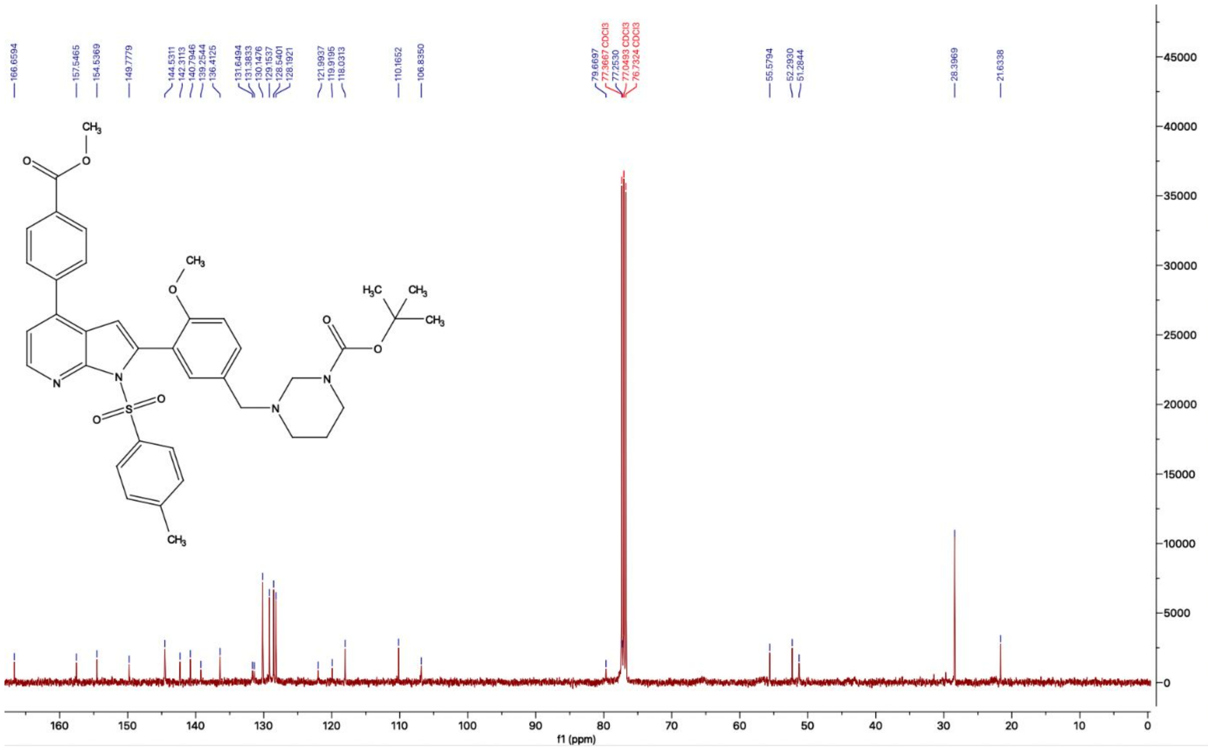

^{1}H and ^{13}C Spectra ($CDCl_3$) for compound 34

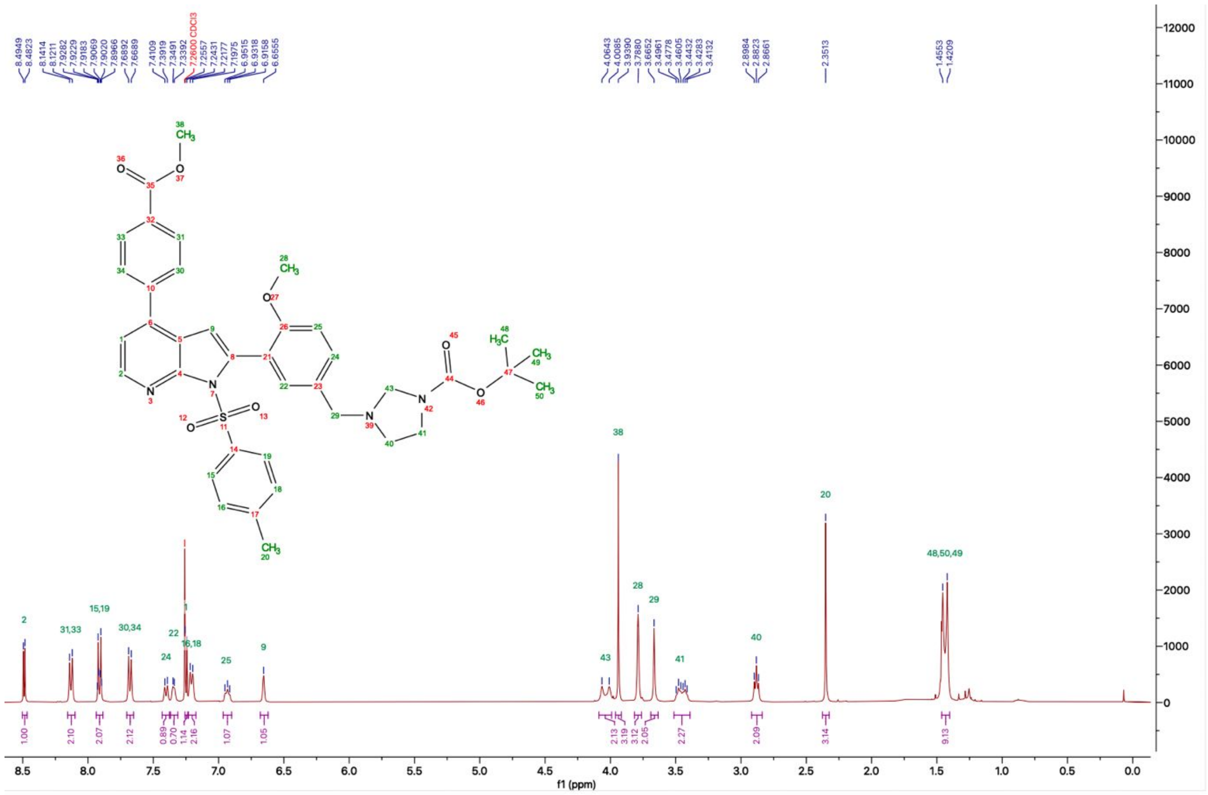

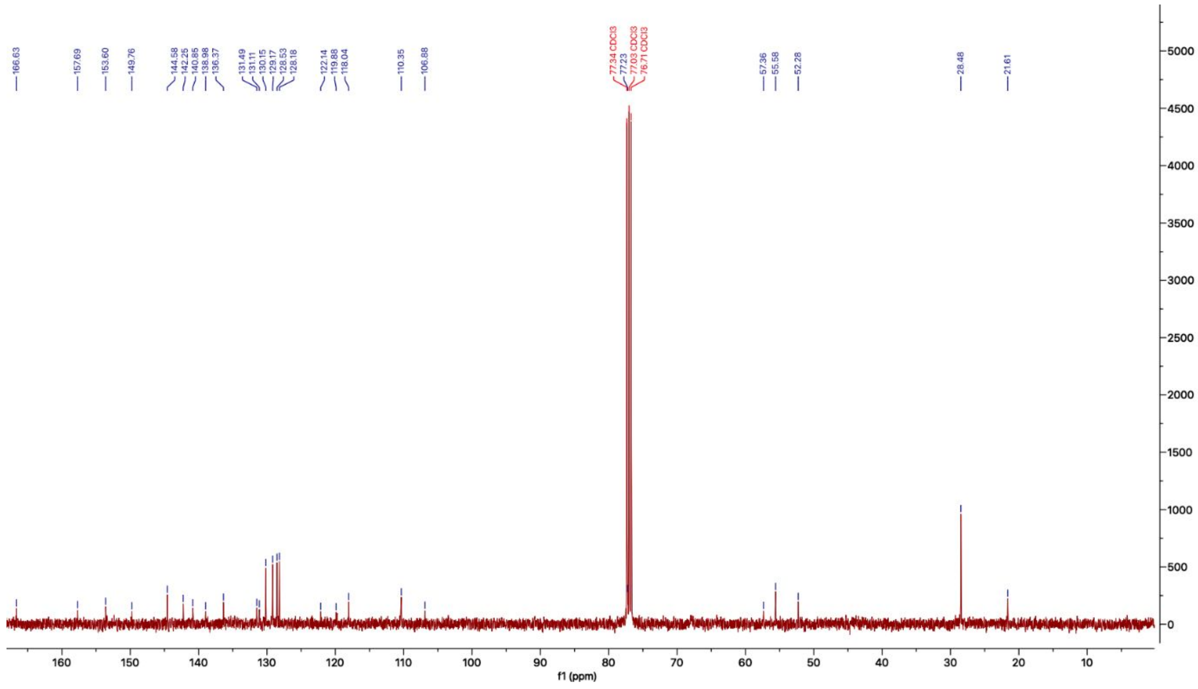

^{1}H and [13]C Spectra ($CDCl_3$) for compound 35

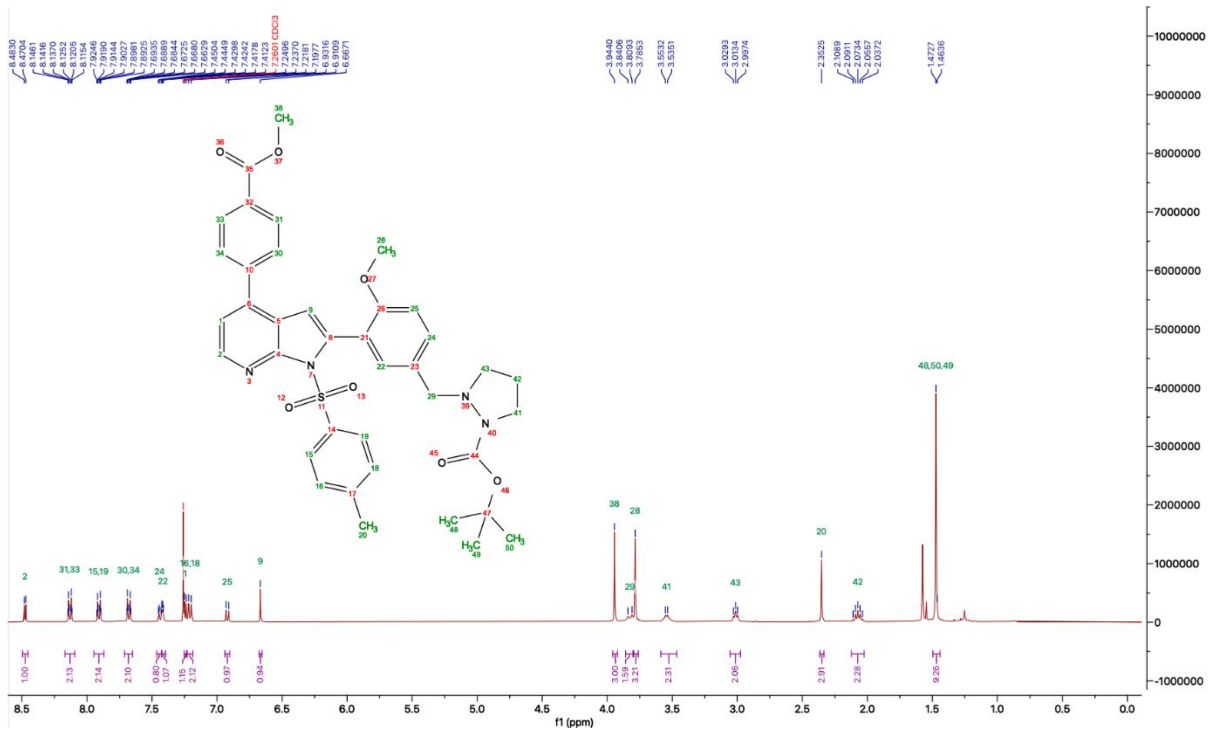

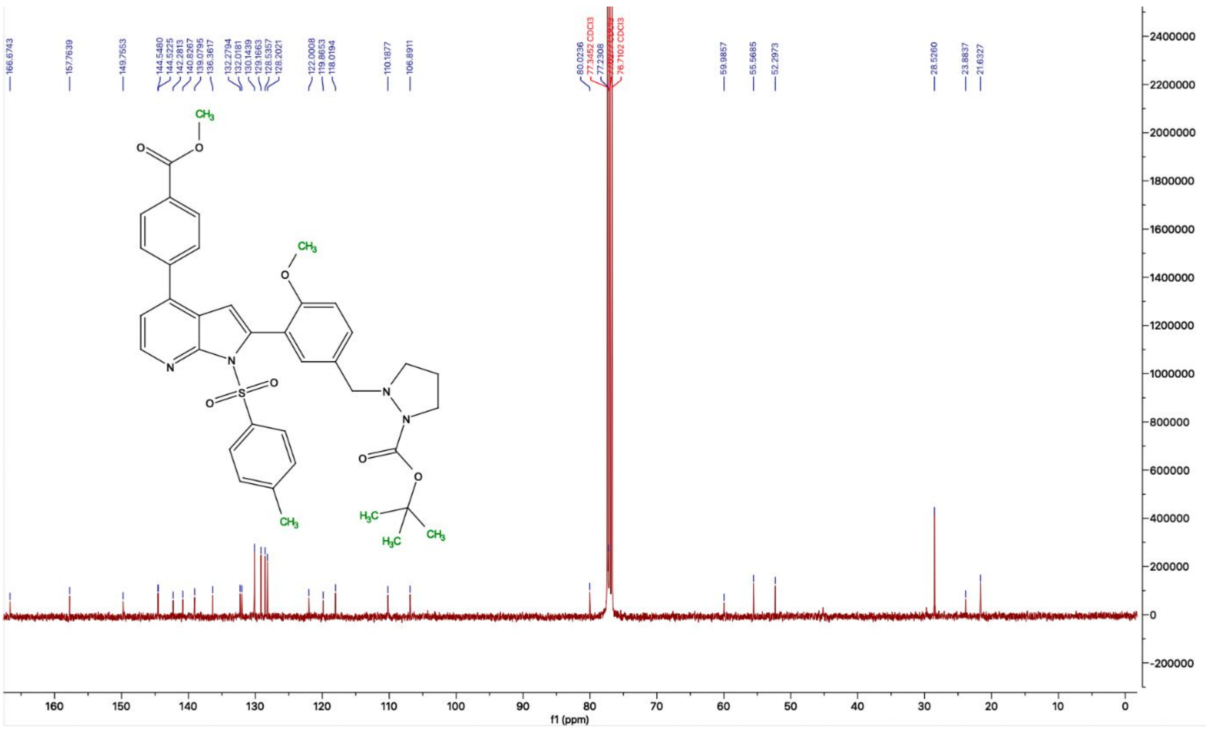

^{1}H and [13]C Spectra ($CDCl_3$) for compound 36

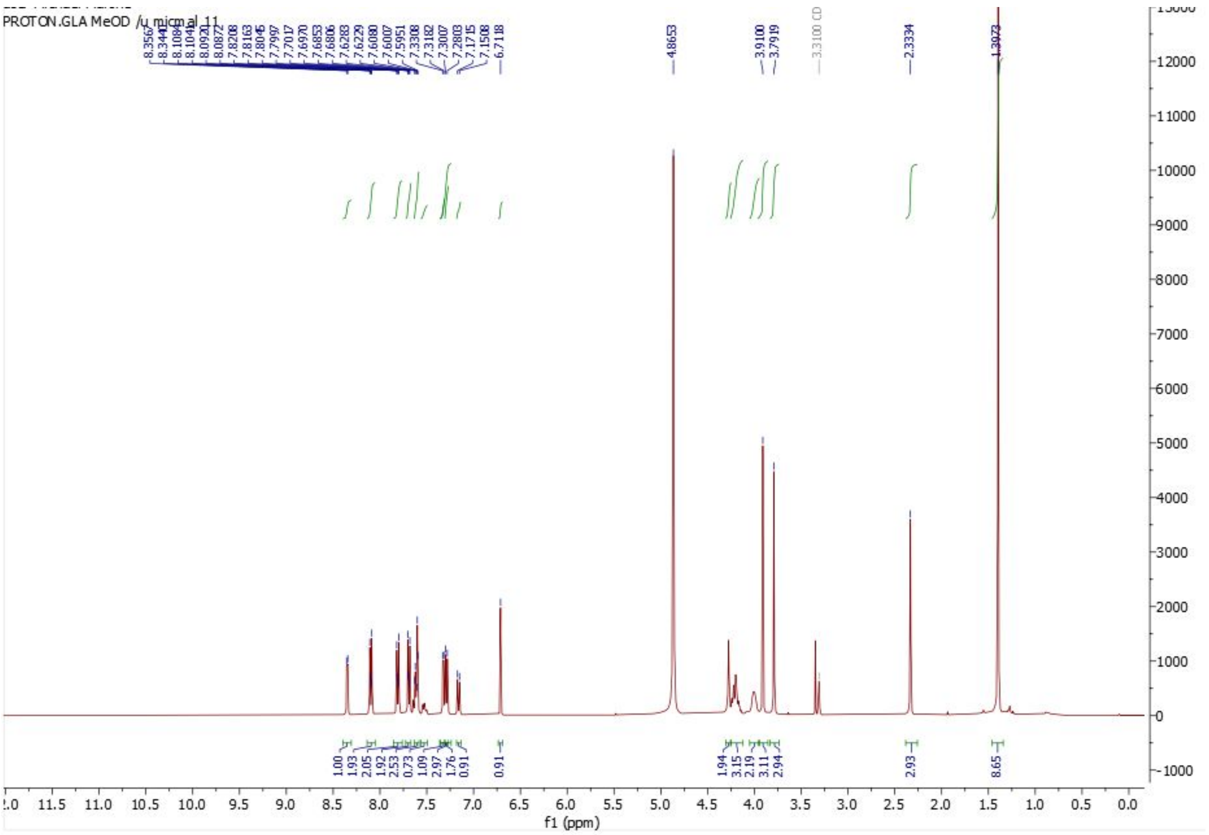

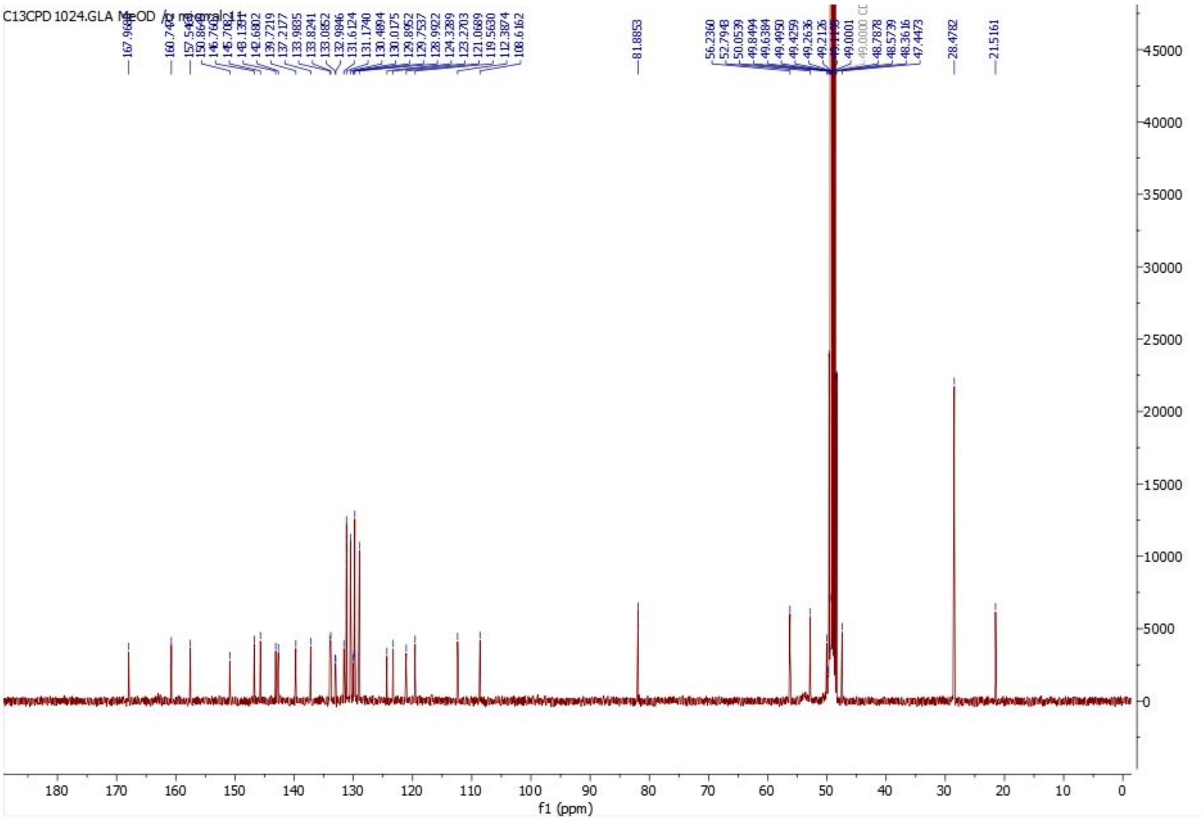

^{1}H and [13]C Spectra (DMSO-d6) for compound 40

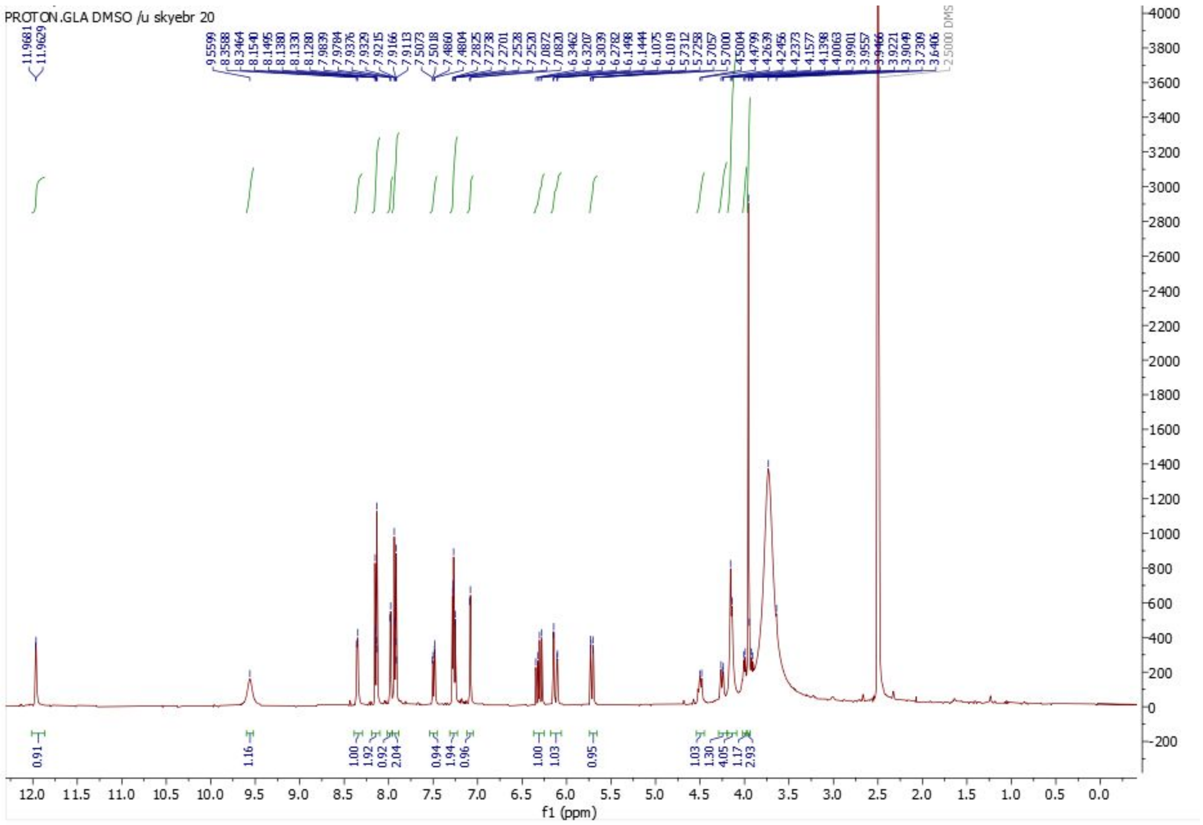

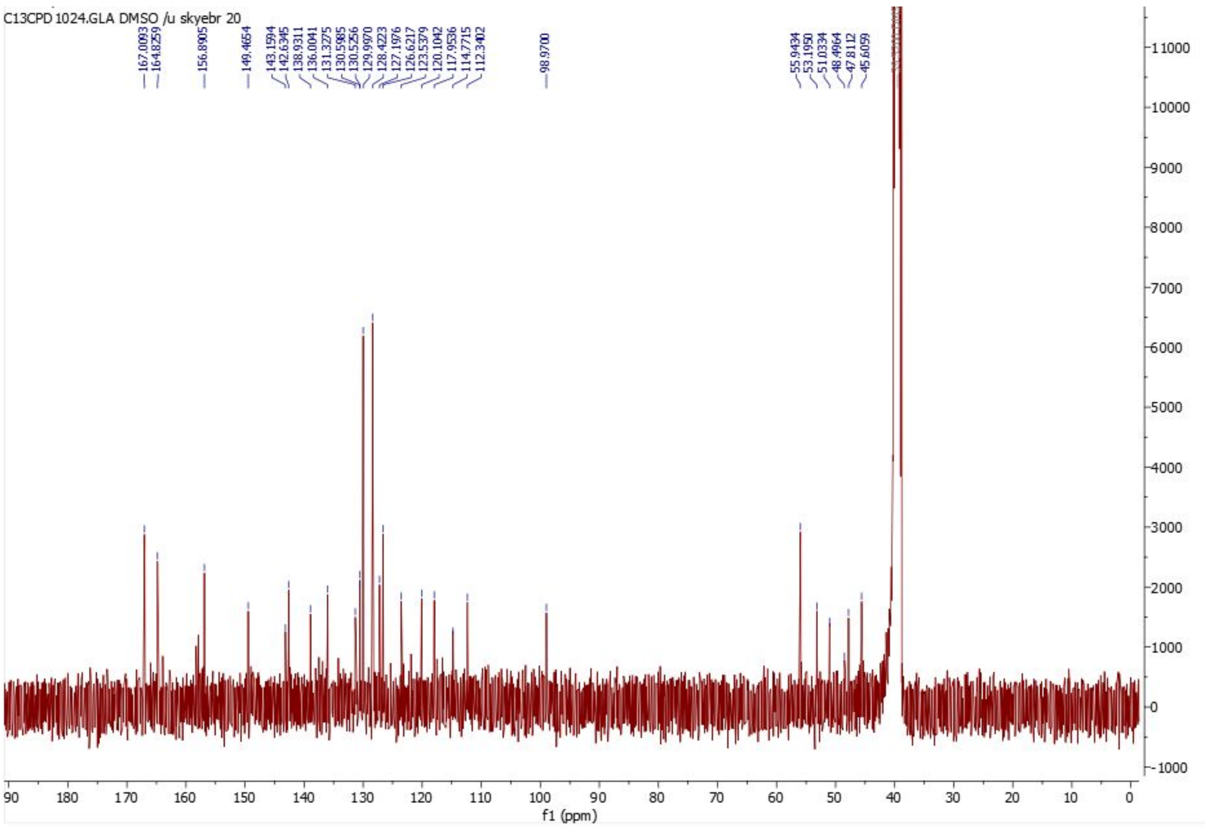

^{1}H and [13]C Spectra (DMSO-d6) for compound 41

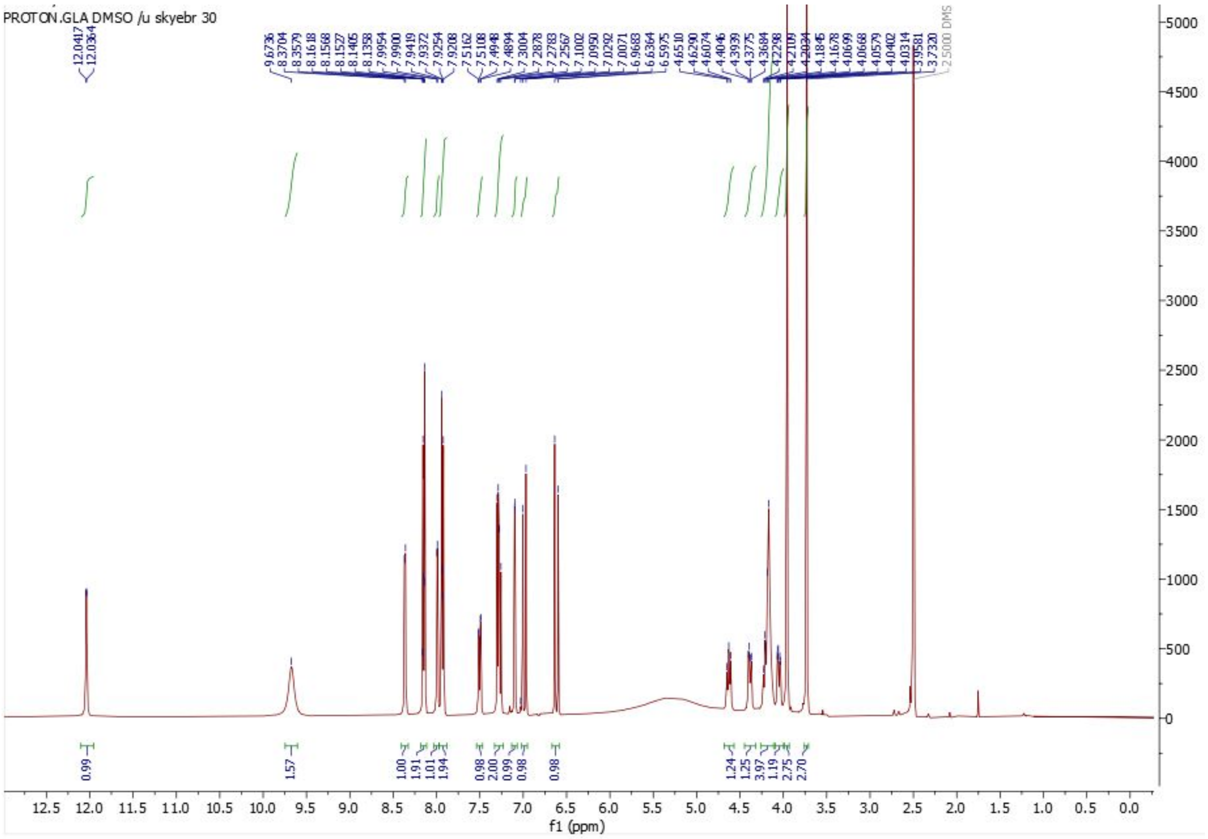

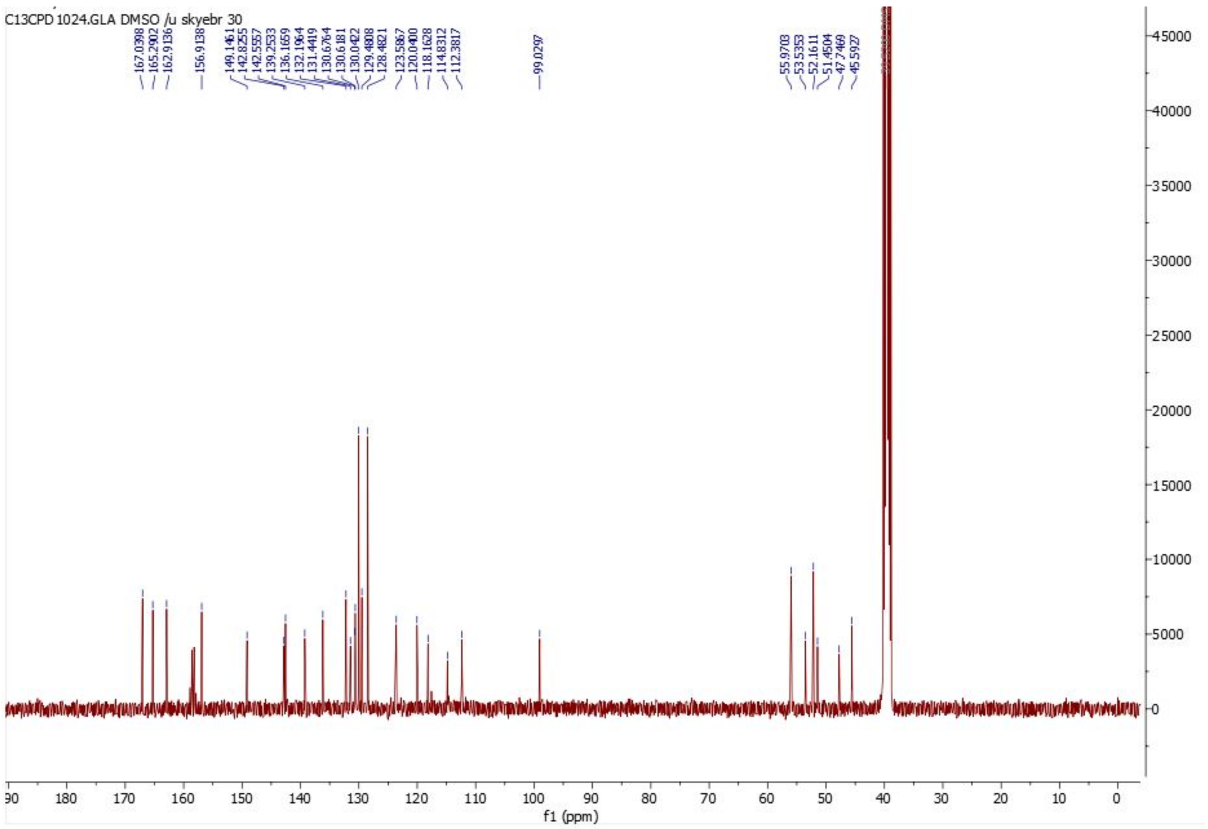

^{1}H and [13]C Spectra (DMSO-d6) for compound 42

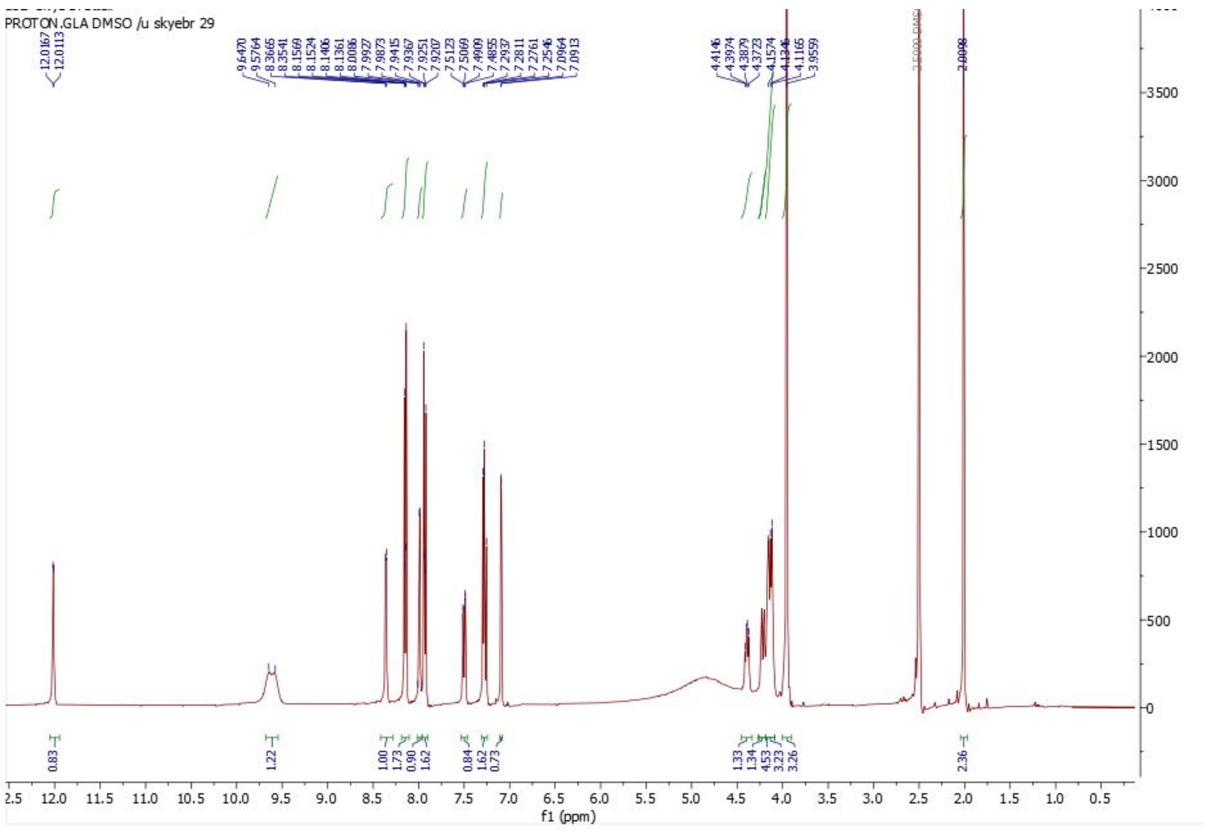

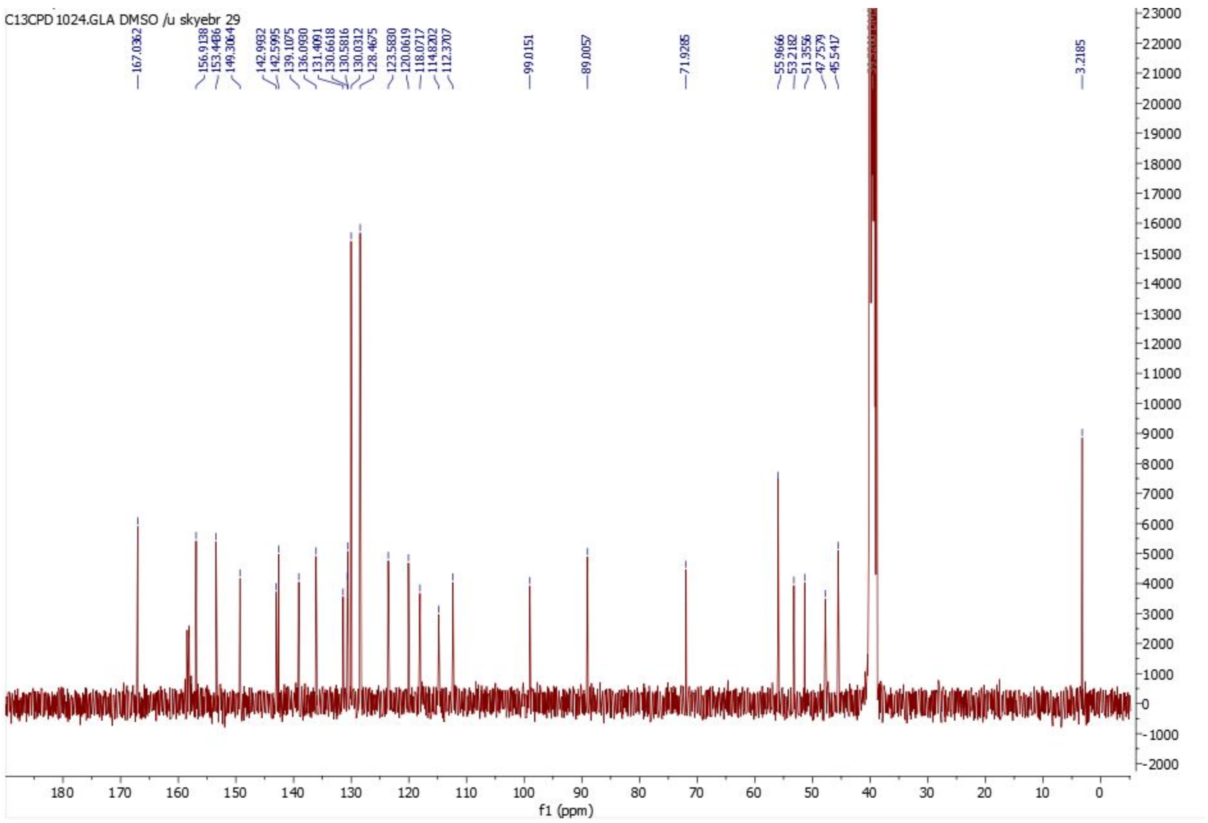

^{1}H and [13]C Spectra (DMSO-d6) for compound 43

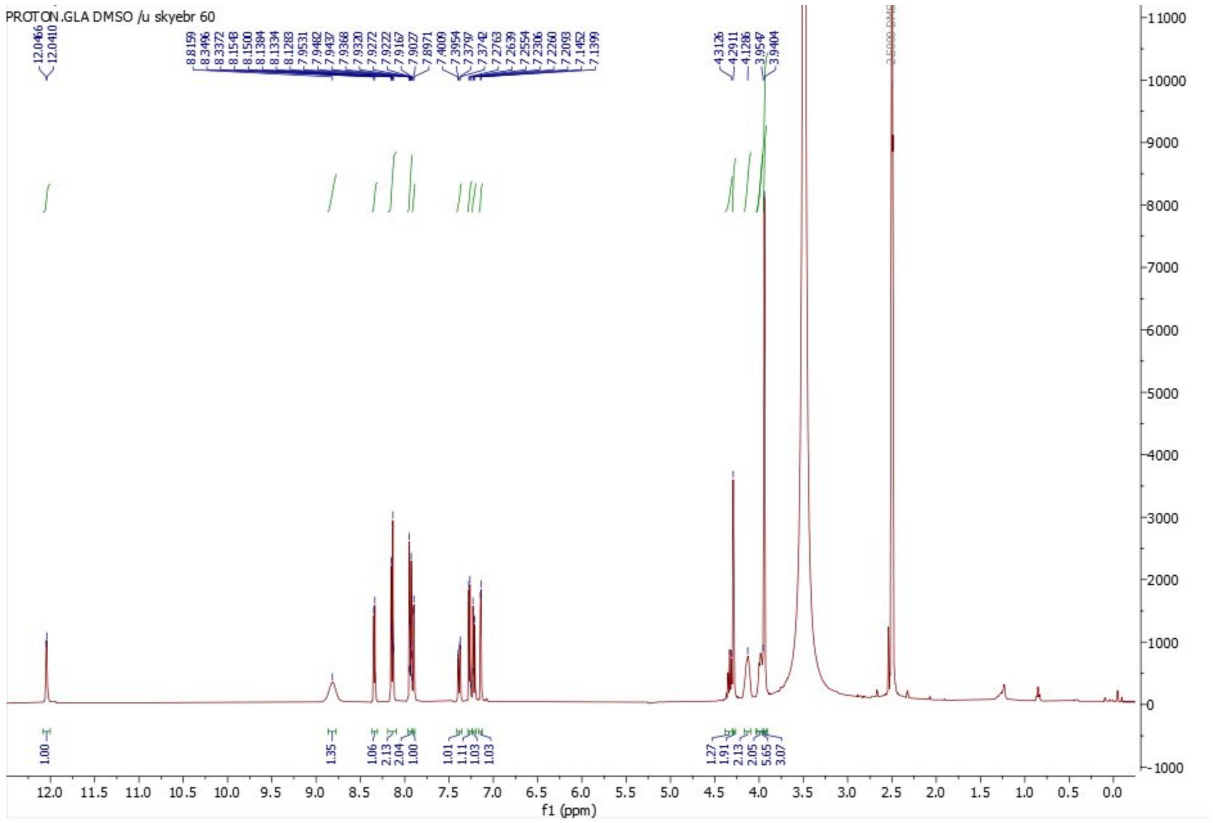

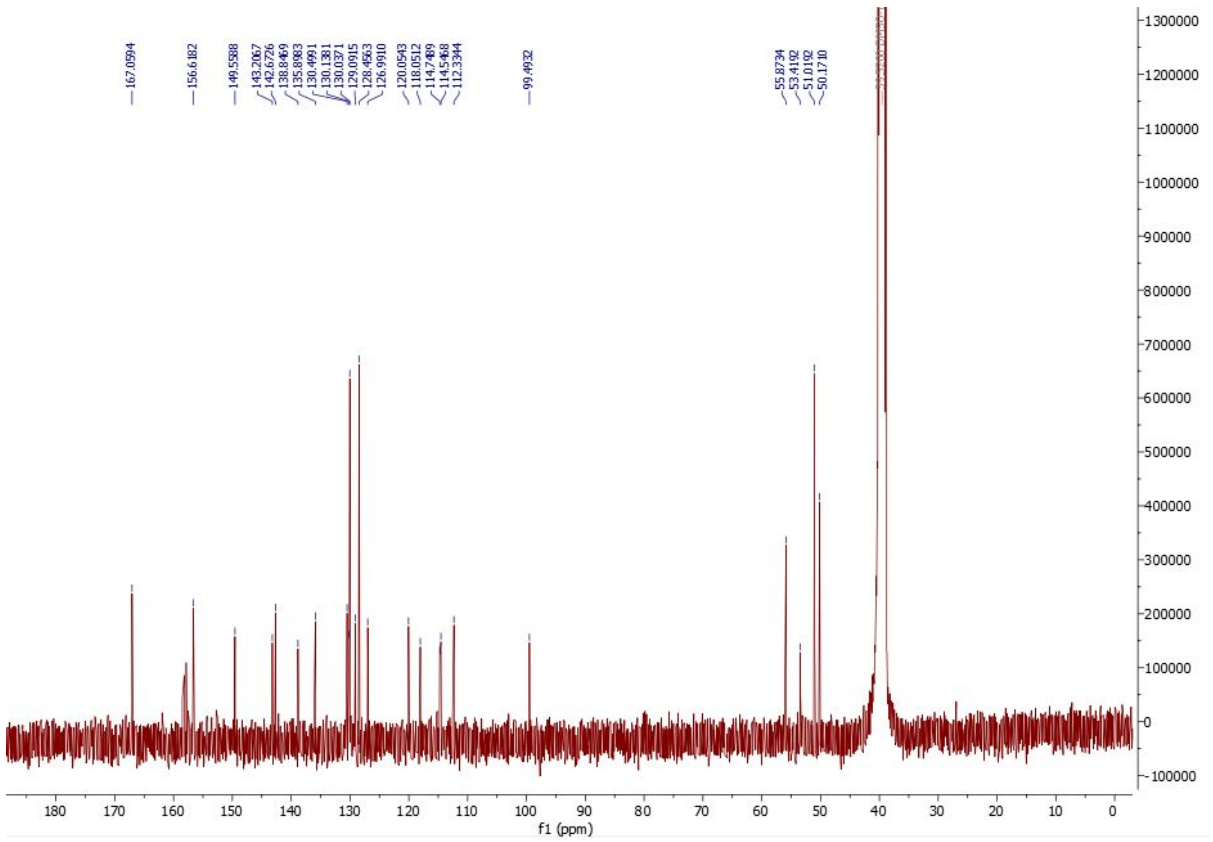

^{1}H and ^{13}C Spectra ($CDCl_3$) for compound 47

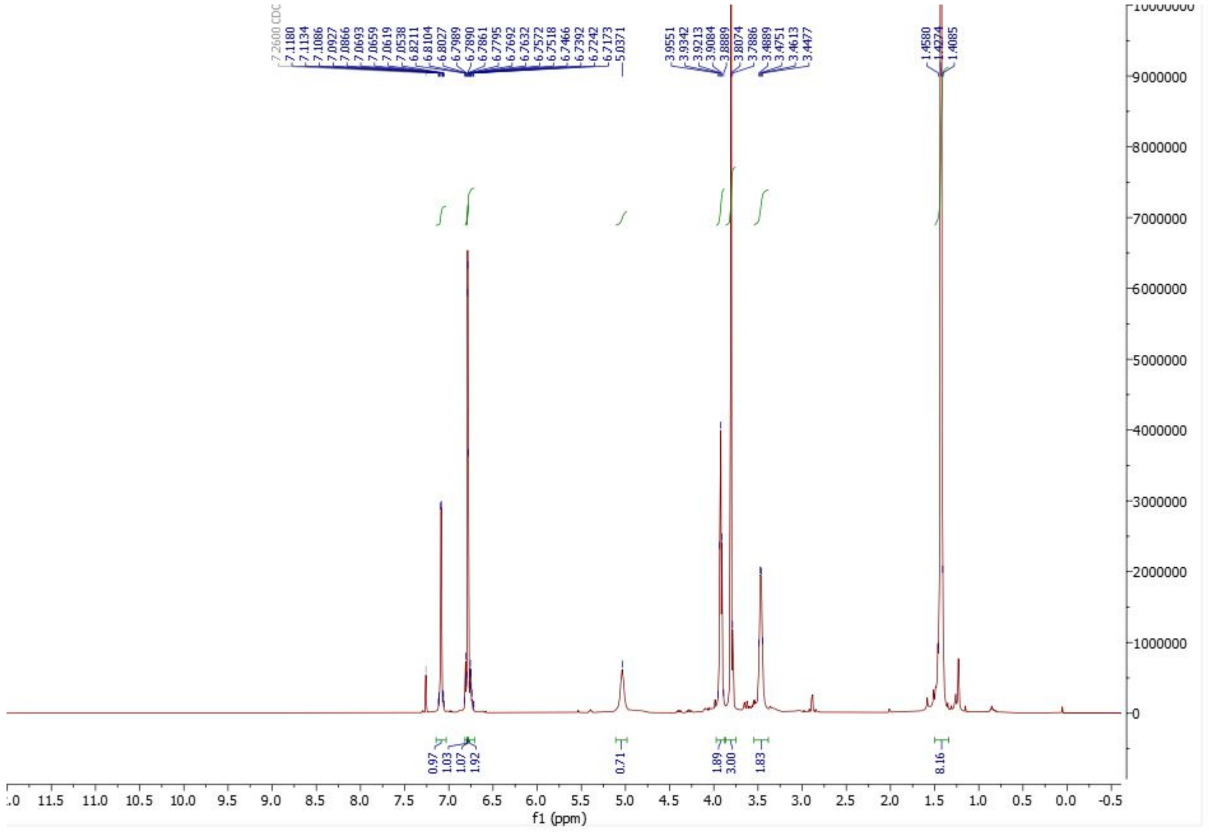

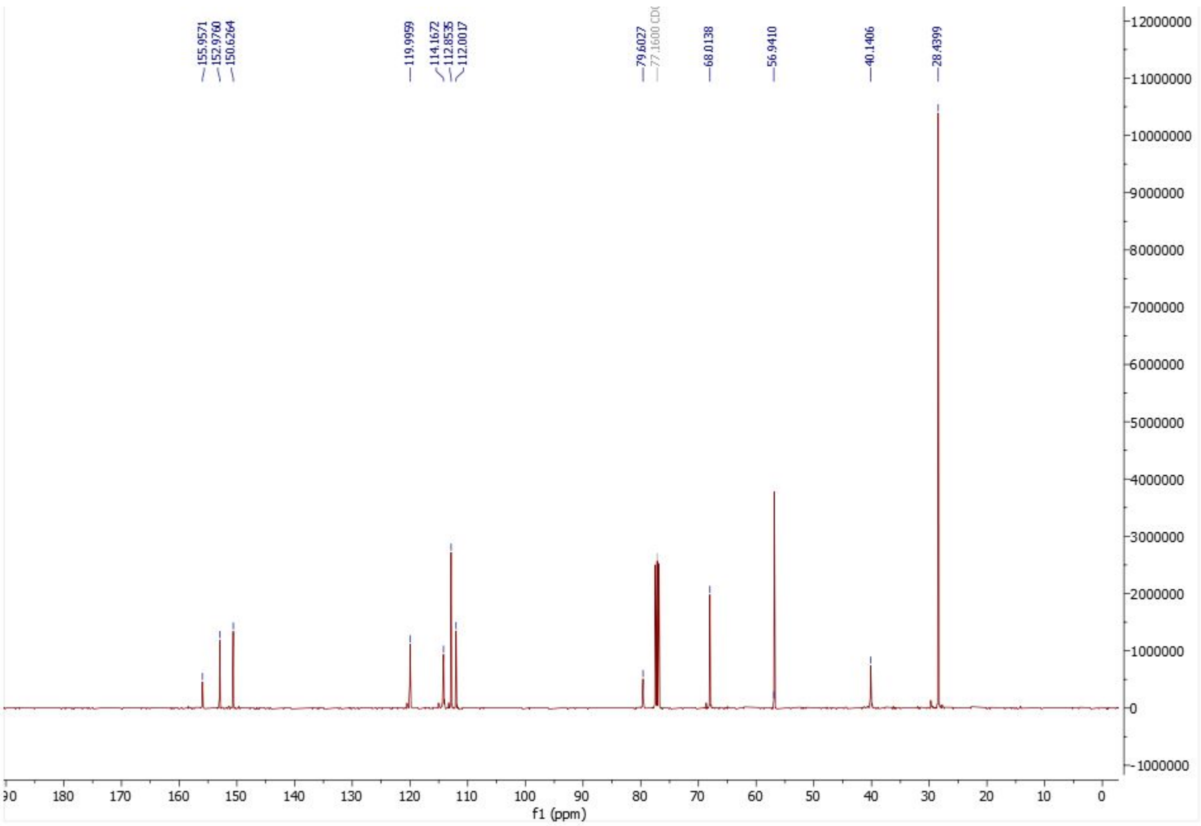

^{1}H and [13]C Spectra ($CDCl_3$) for compound 48

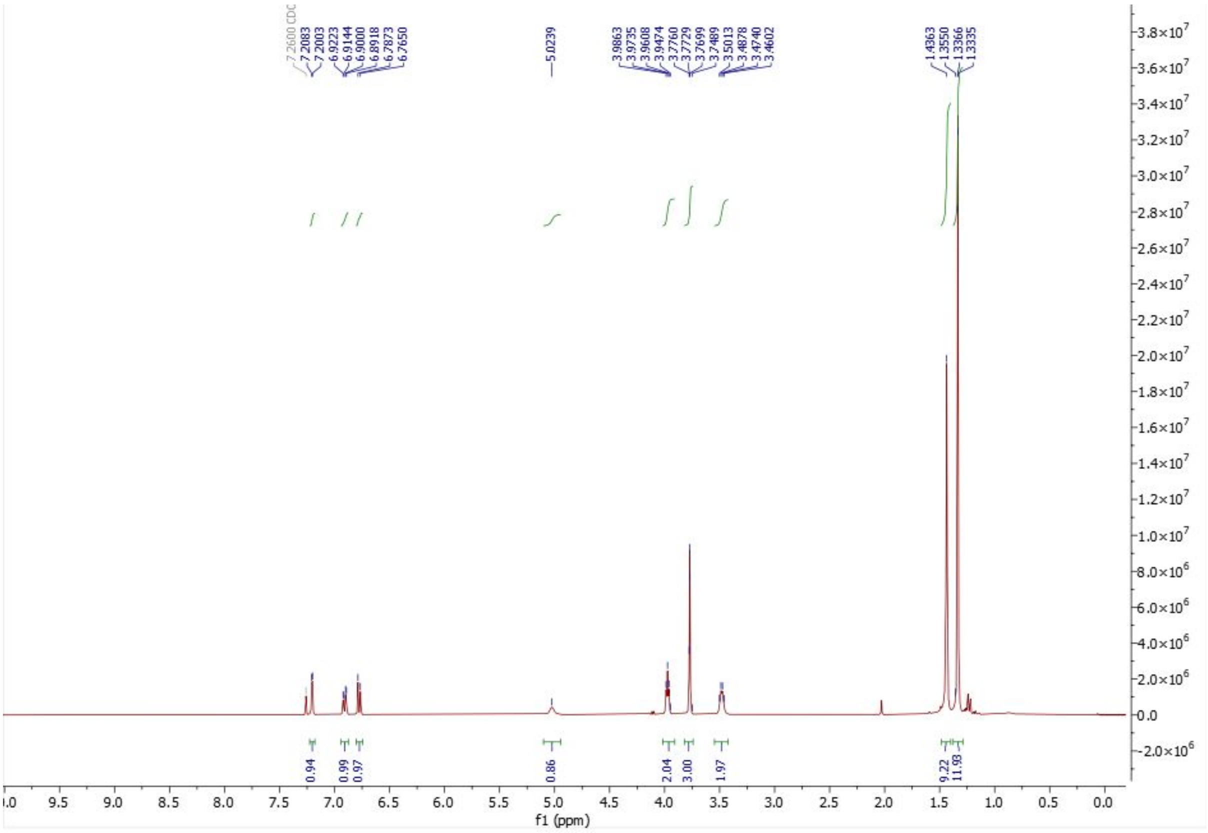

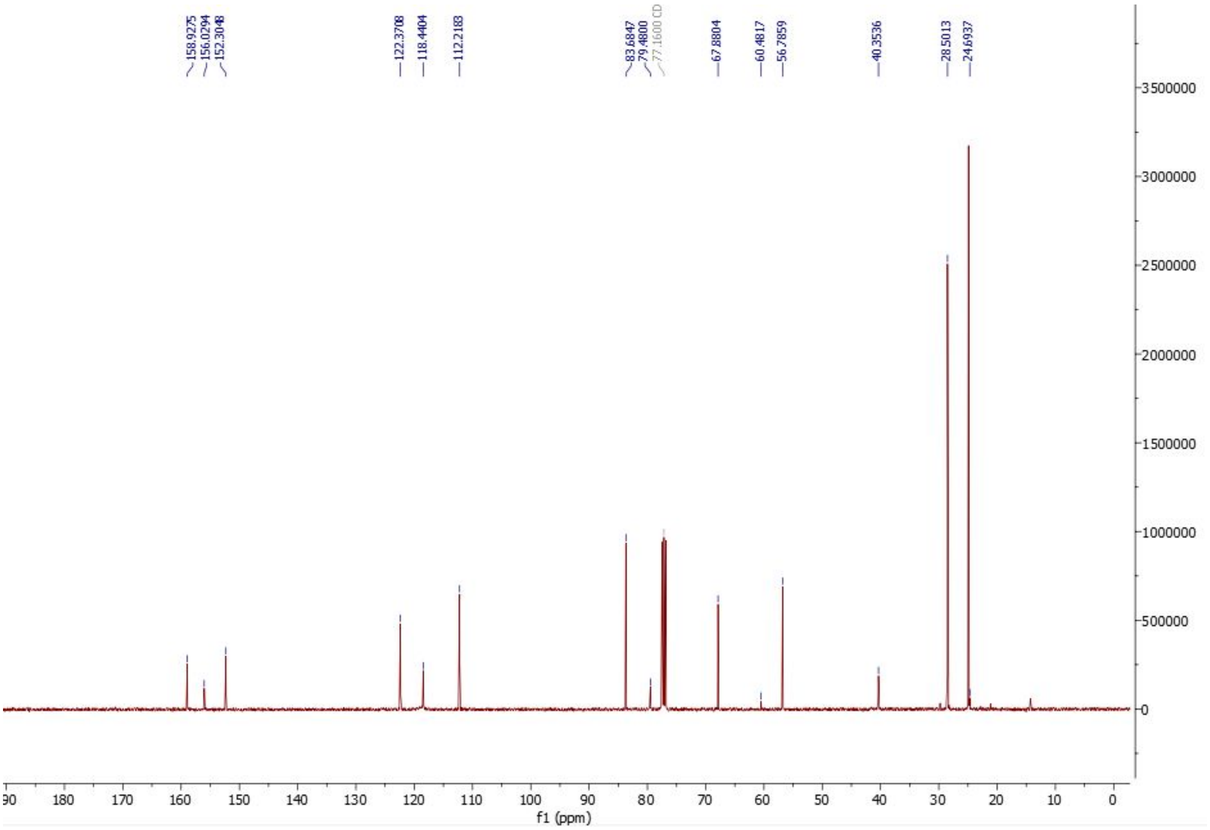

^{1}H and [13]C Spectra ($CDCl_3$) for compound 50

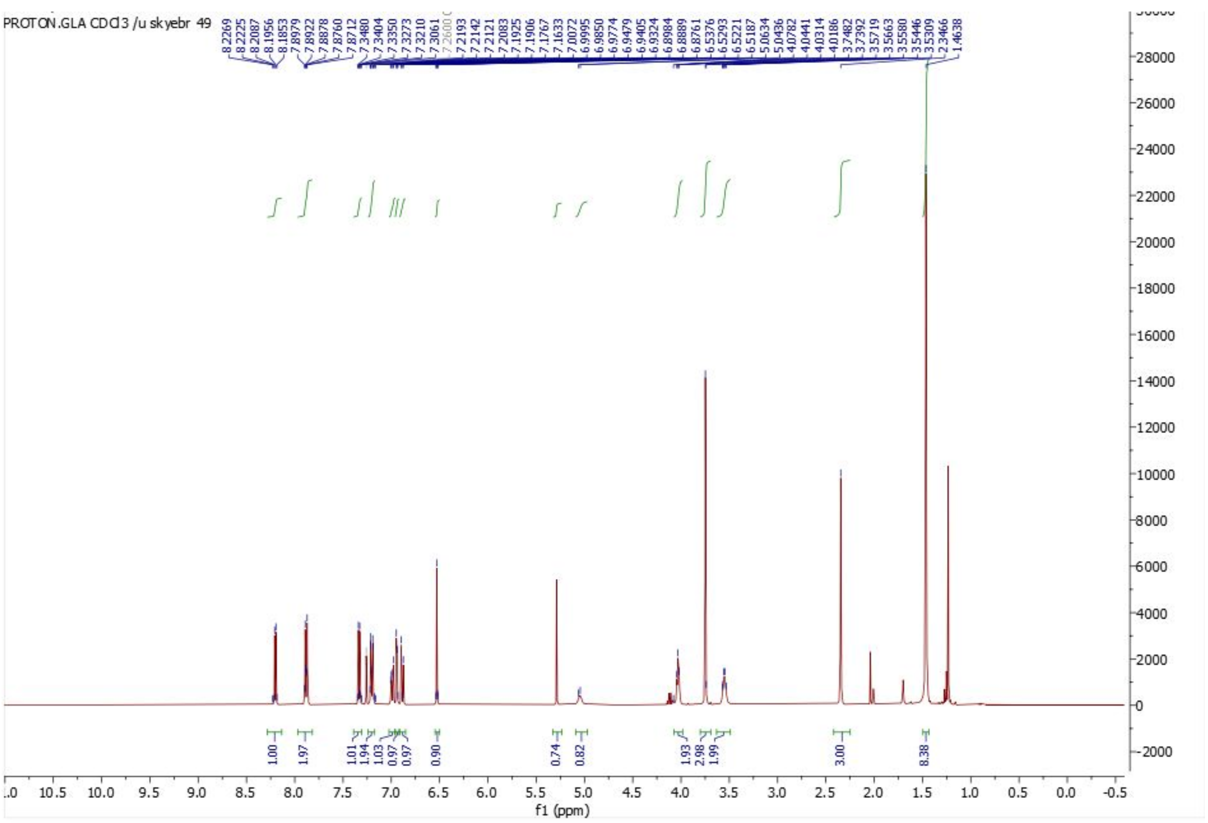

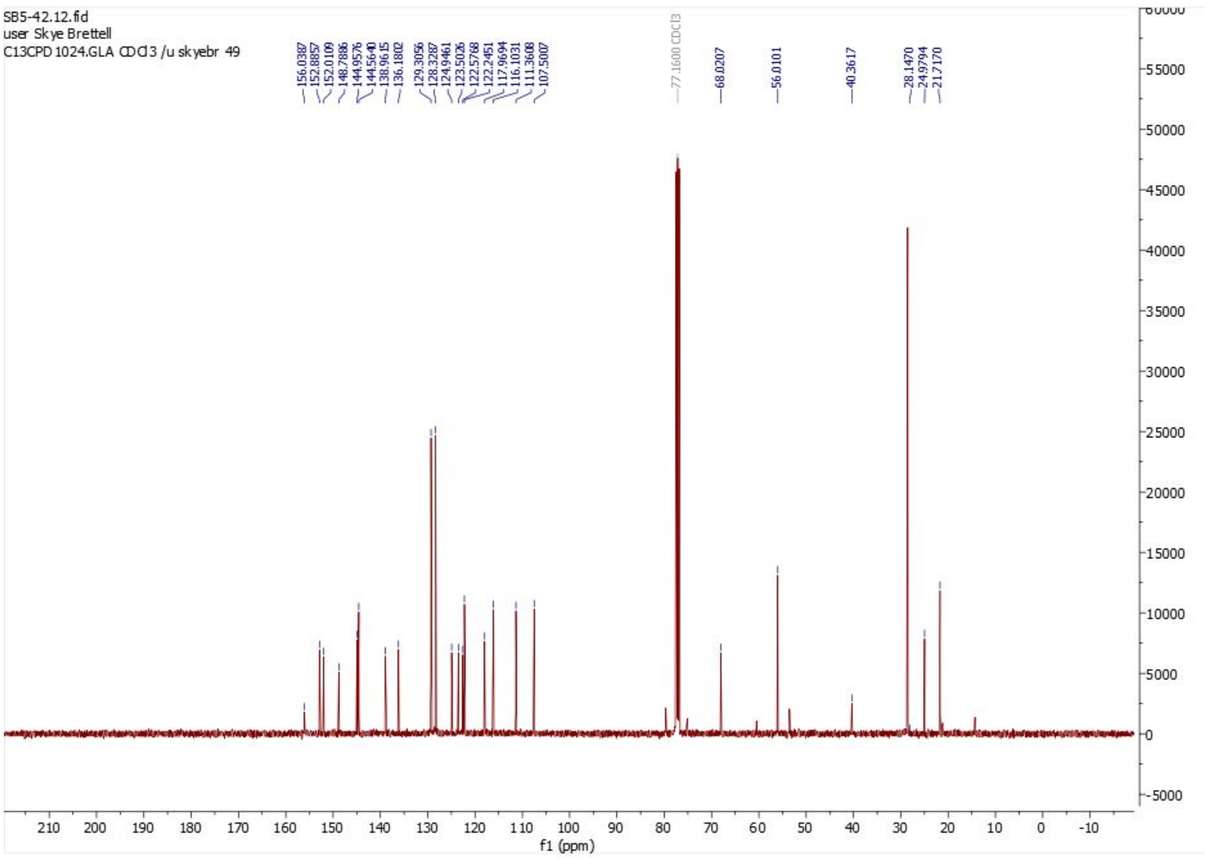

^{1}H and [13]C Spectra ($CDCl_3$) for compound 51

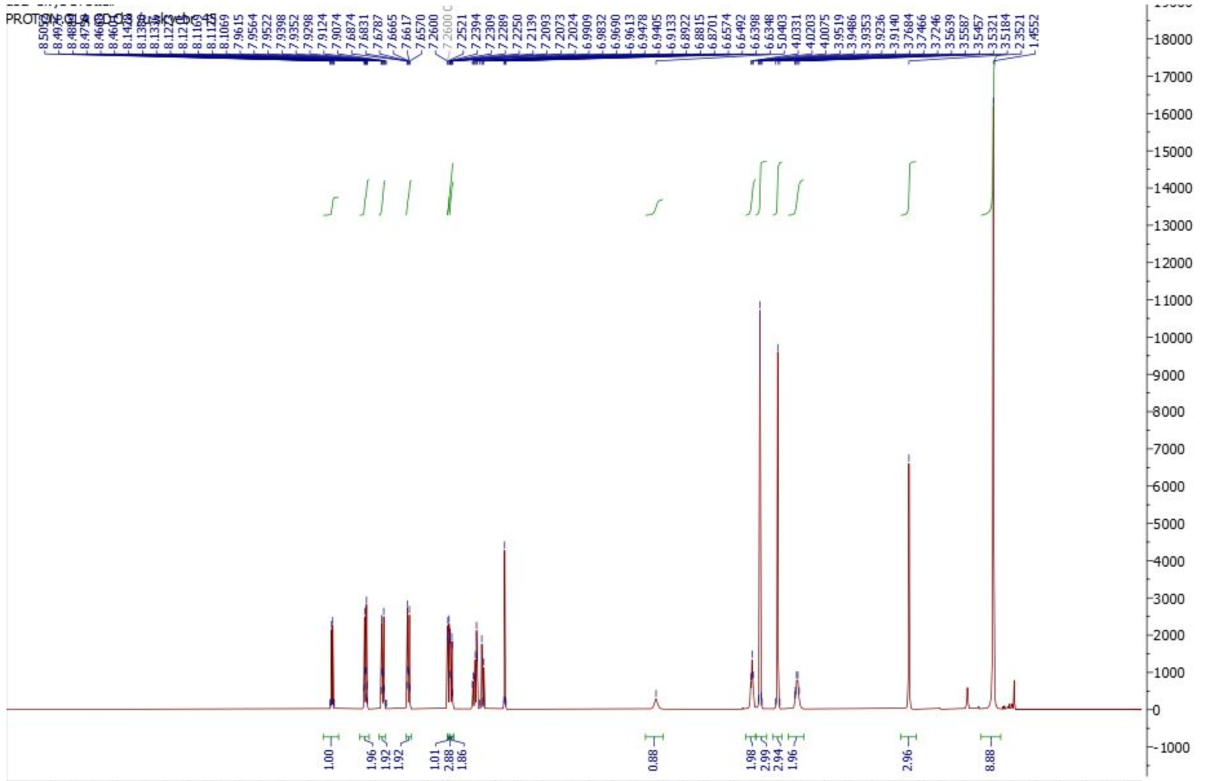

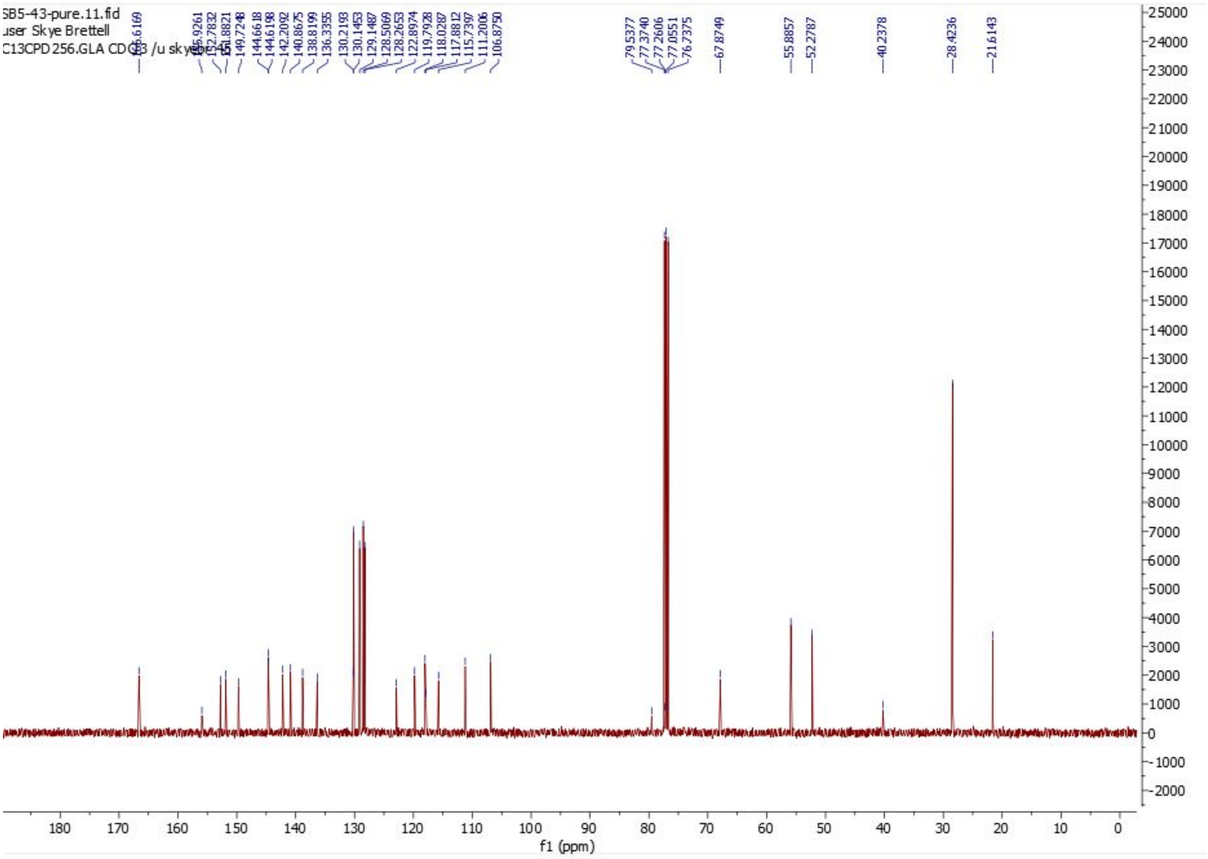

^{1}H and [13]C Spectra (DMSO-d6) for compound 53

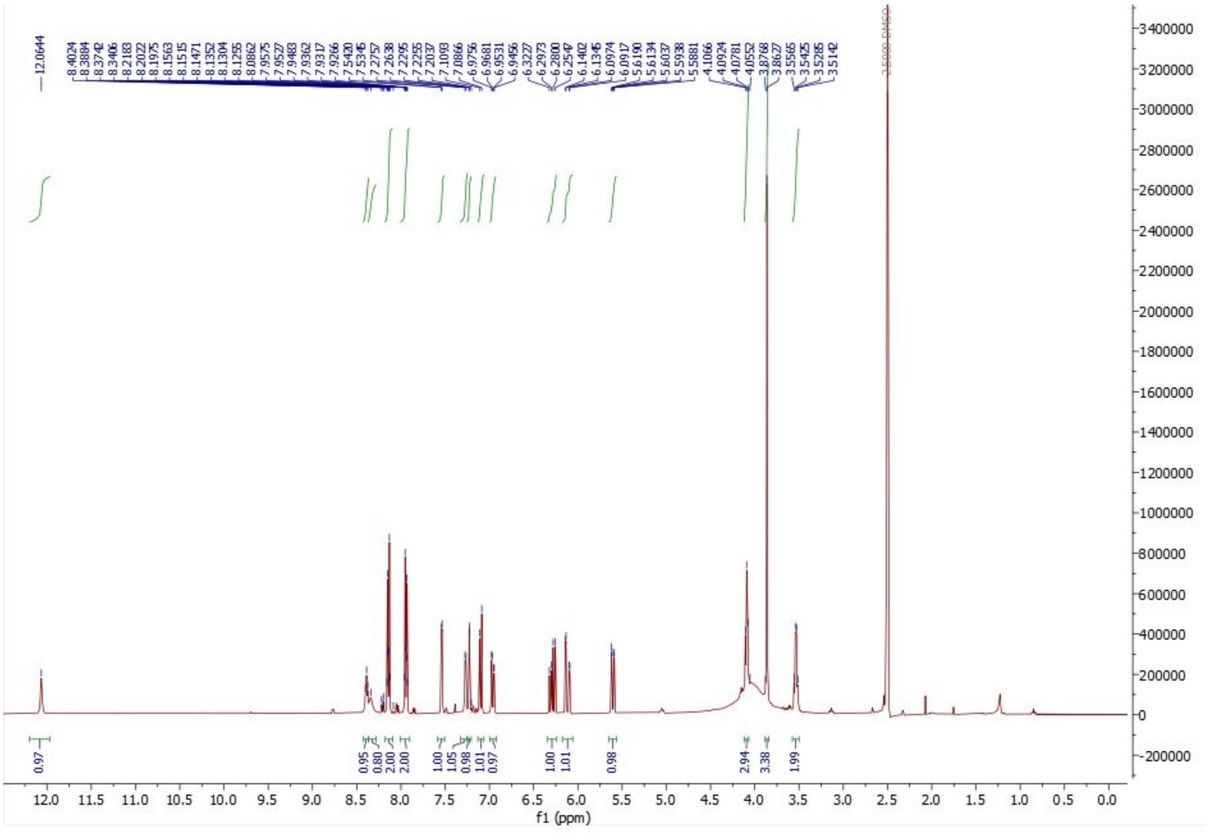

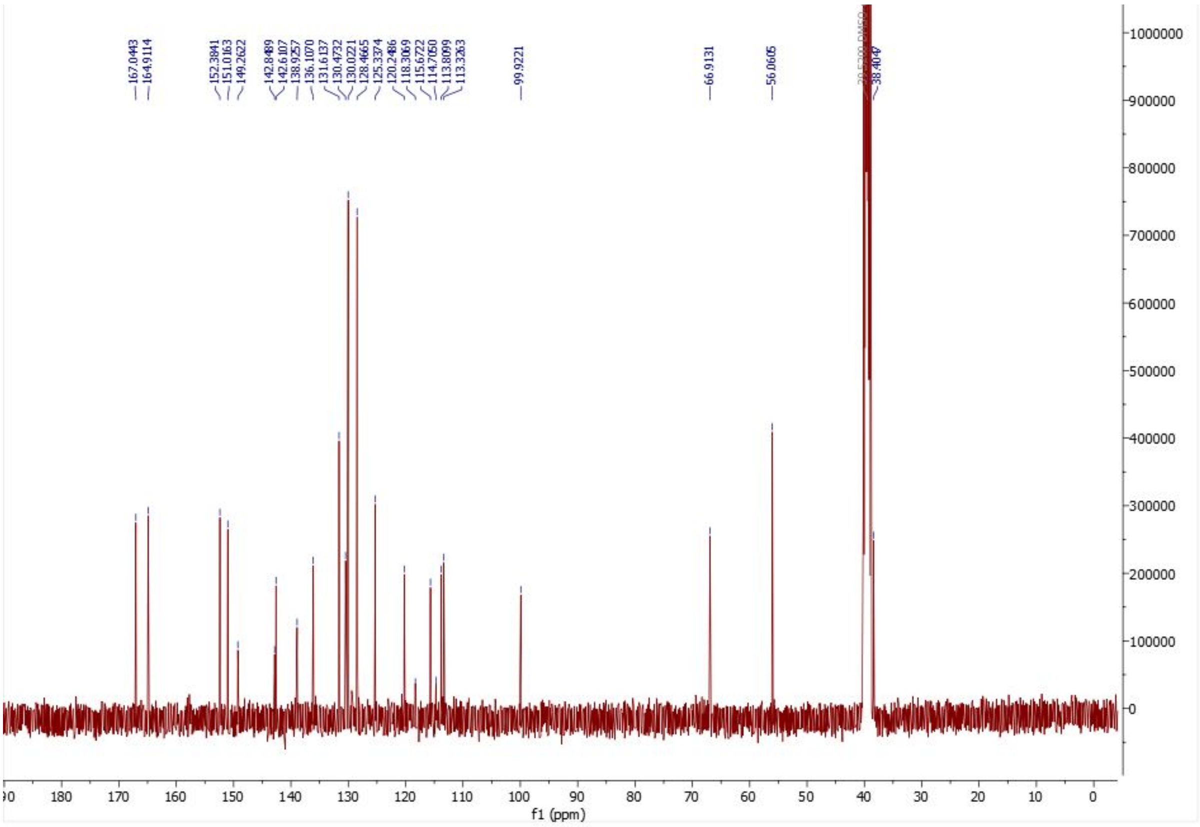

^{1}H and [13]C Spectra (DMSO-d6) for compound 54

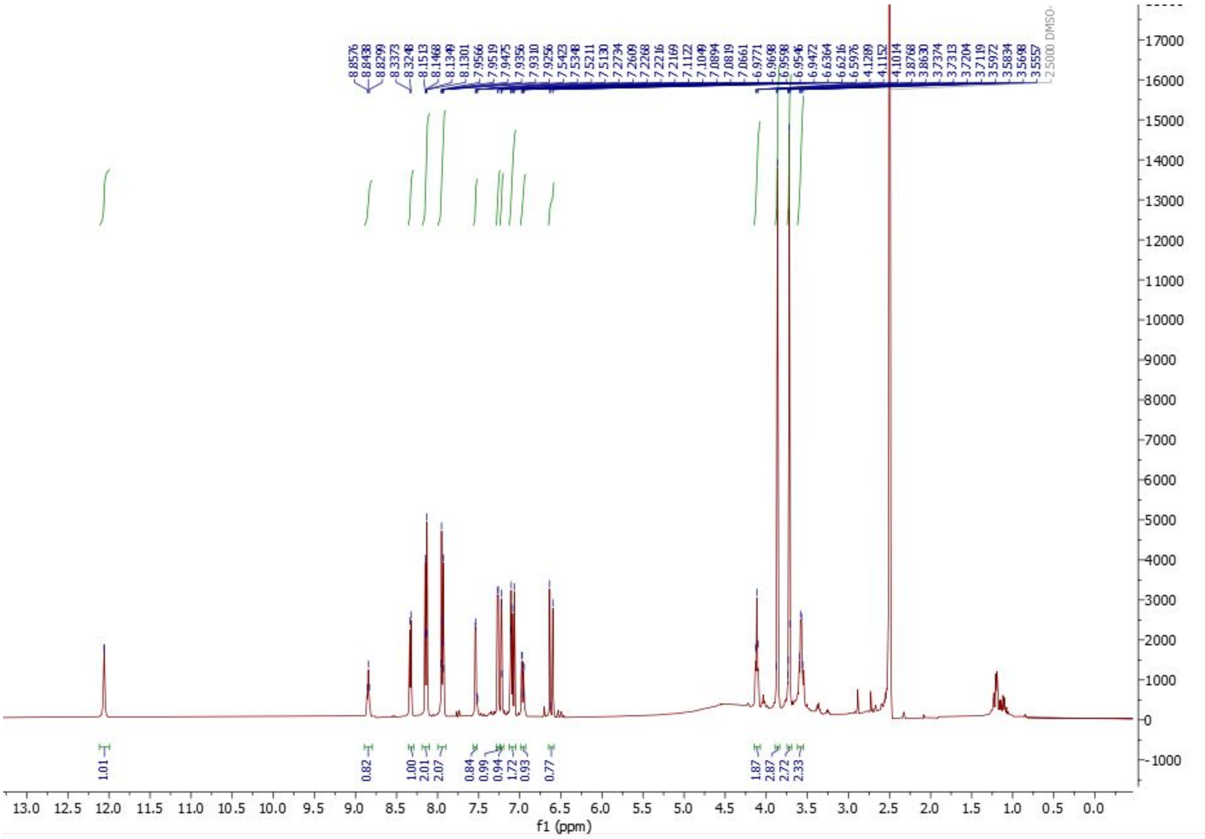

HPLC traces for Novel Compounds

HPLC Data for Compound 8

(5-95% ACN 0.1% TFA in H_2O 0.1% TFA over 50 minutes)

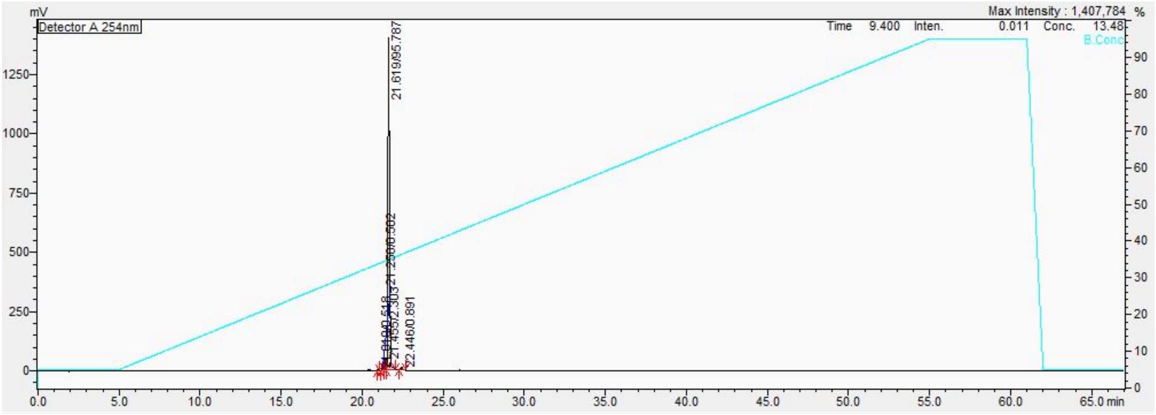

HPLC Data for Compound 9

(5-95% ACN 0.1% TFA in H_2O 0.1% TFA over 50 minutes)

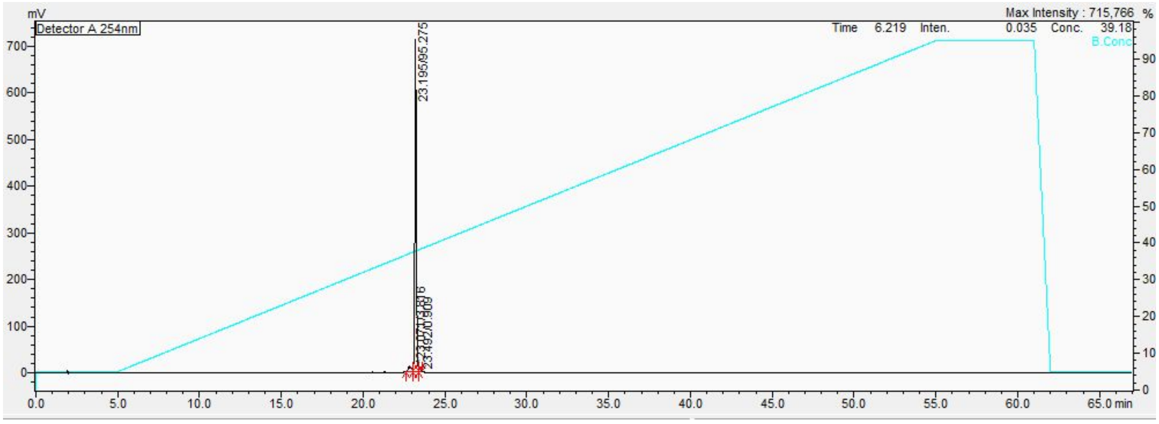

HPLC Data for Compound 10

(5-95% ACN 0.1% TFA in H_2O 0.1% TFA over 60 minutes)

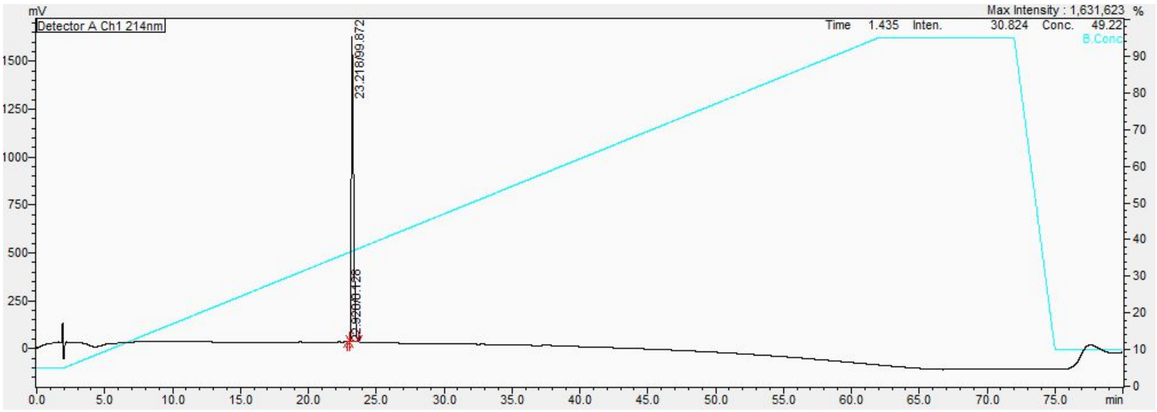

HPLC Data for Compound 12

(5-95% ACN 0.1% TFA in H_2O 0.1% TFA over 50 minutes)

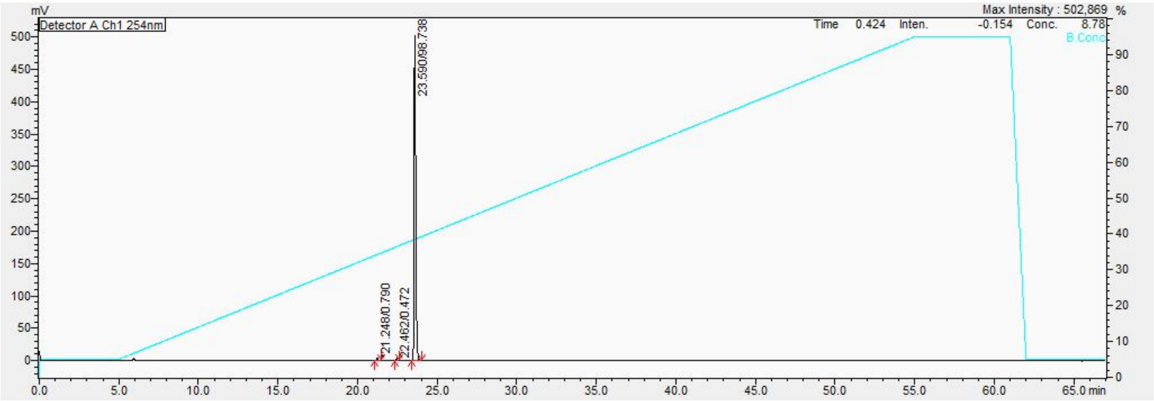

HPLC Data for Compound 27

(5-95% ACN 0.1% TFA in H_2O 0.1% TFA over 50 minutes)

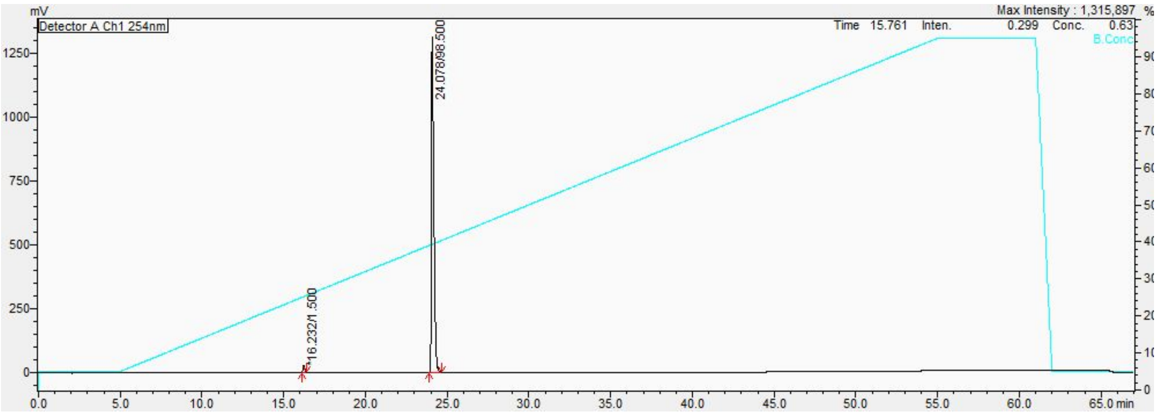

HPLC Data for Compound 28

(5-95% ACN 0.1% TFA in H_2O 0.1% TFA over 50 minutes)

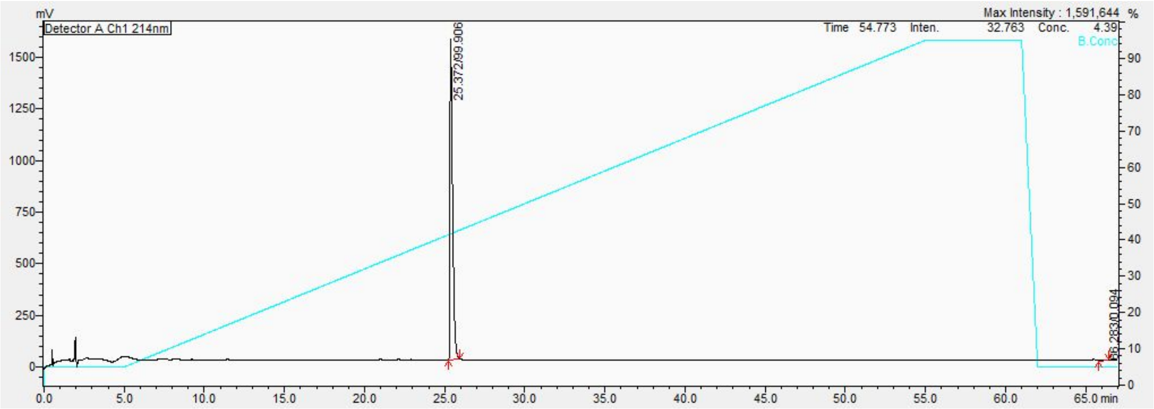

HPLC Data for Compound 29

(5-95% ACN 0.1% TFA in H_2O 0.1% TFA over 50 minutes)

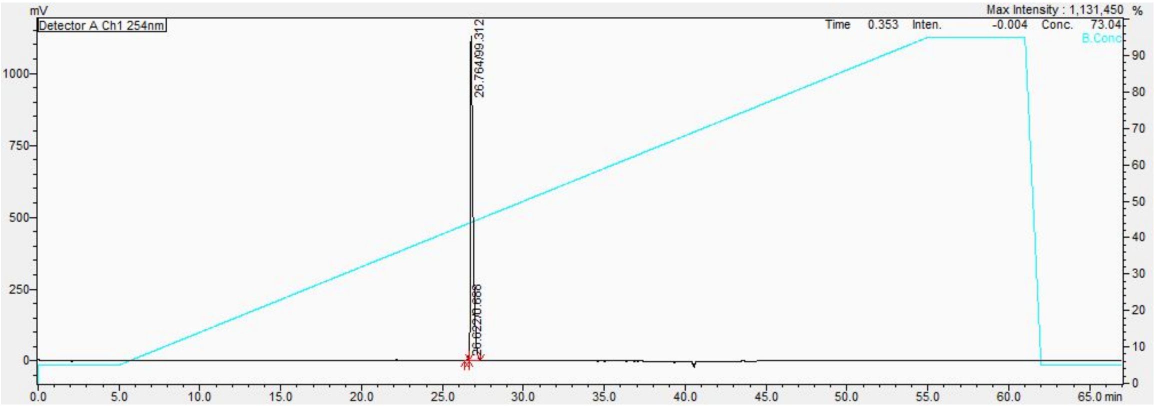

HPLC Data for Compound 13

(5-95% ACN 0.1% TFA in H_2O 0.1% TFA over 50 minutes)

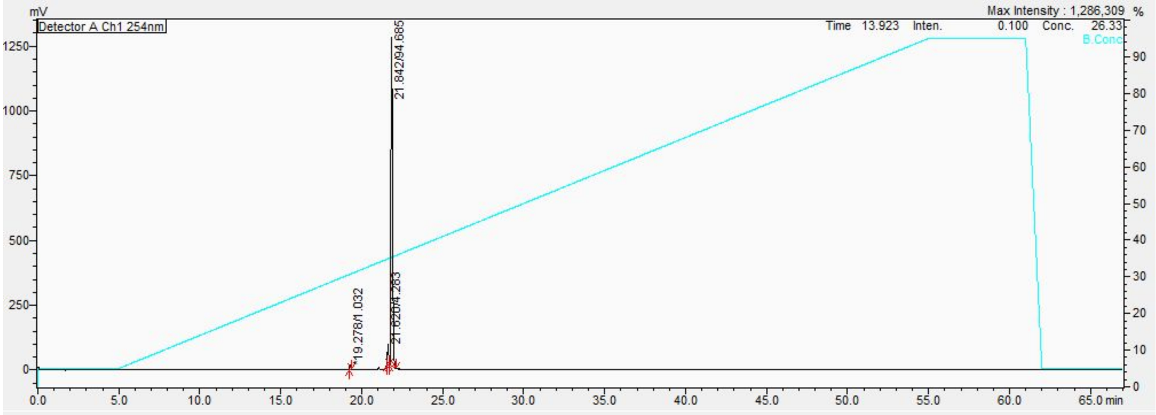

HPLC Data for Compound 14

(5-95% ACN 0.1% TFA in H_2O 0.1% TFA over 50 minutes)

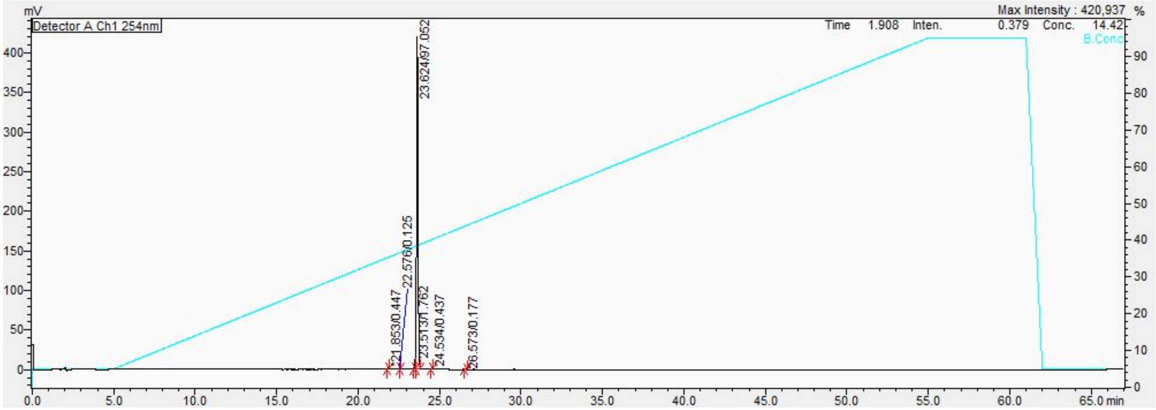

HPLC Data for Compound 15

(5-95% ACN 0.1% TFA in H_2O 0.1% TFA over 50 minutes)

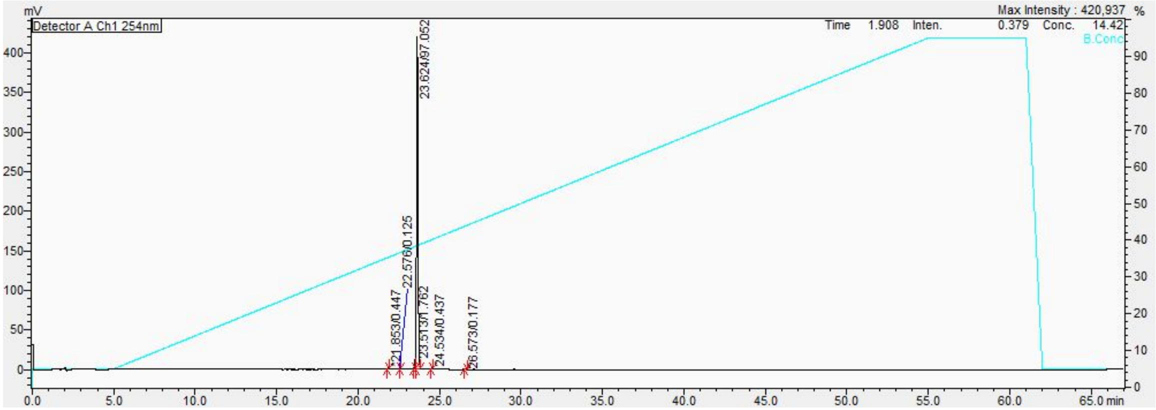

HPLC Data for Compound 16

(5-95% ACN 0.1% TFA in H_2O 0.1% TFA over 50 minutes)

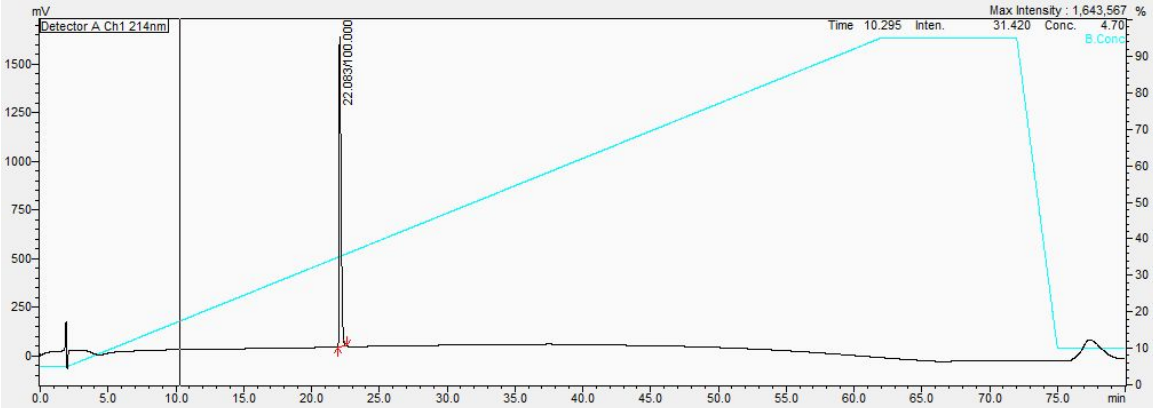

HPLC Data for Compound 40

(5-95% ACN 0.1% TFA in H_2O 0.1% TFA over 50 minutes)

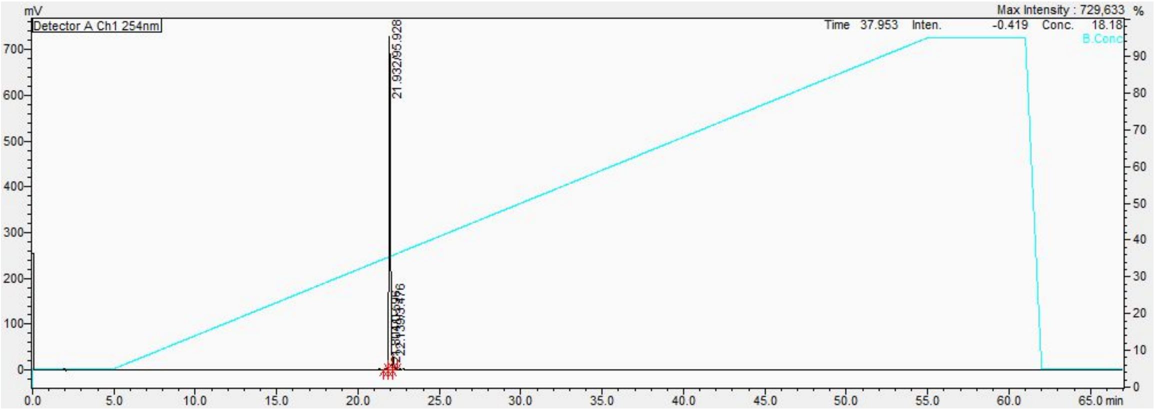

HPLC Data for Compound 41

(5-95% ACN 0.1% TFA in H_2O 0.1% TFA over 50 minutes)

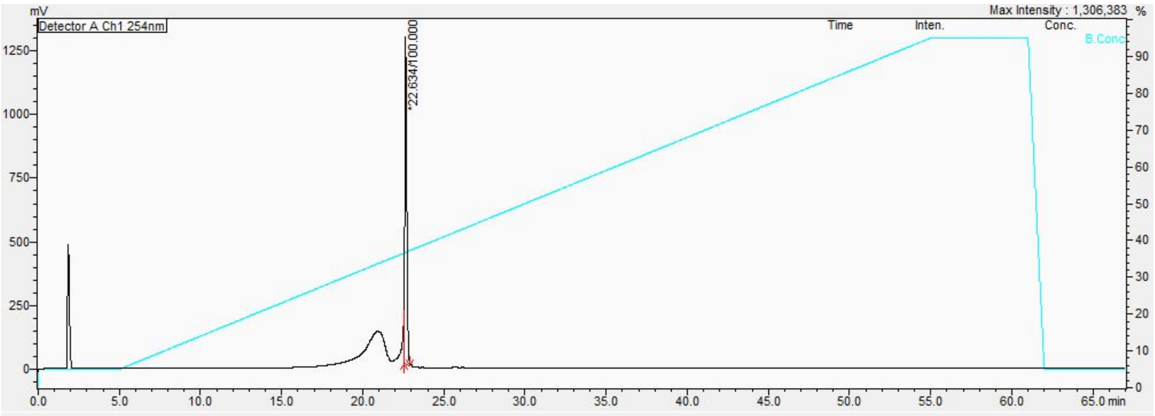

HPLC Data for Compound 42

(5-95% ACN 0.1% TFA in H_2O 0.1% TFA over 50 minutes)

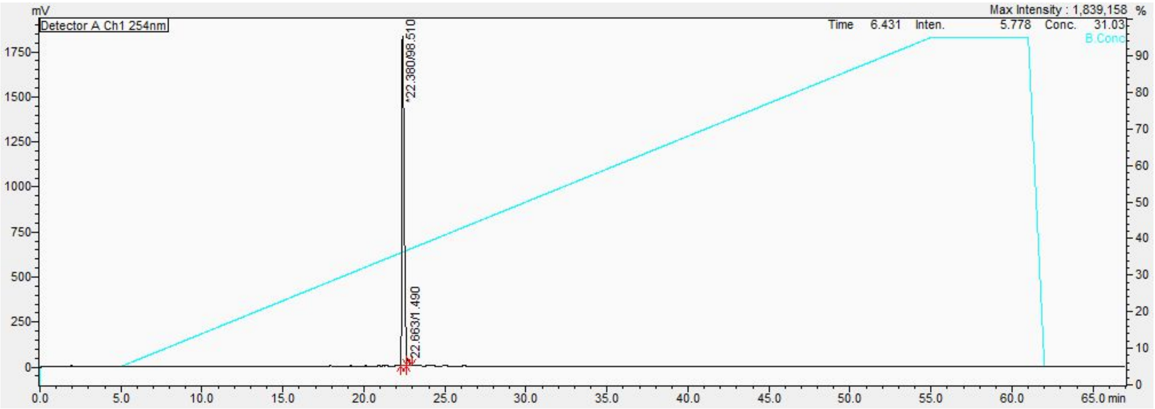

HPLC Data for Compound 43

(5-95% ACN 0.1% TFA in H_2O 0.1% TFA over 60 minutes)

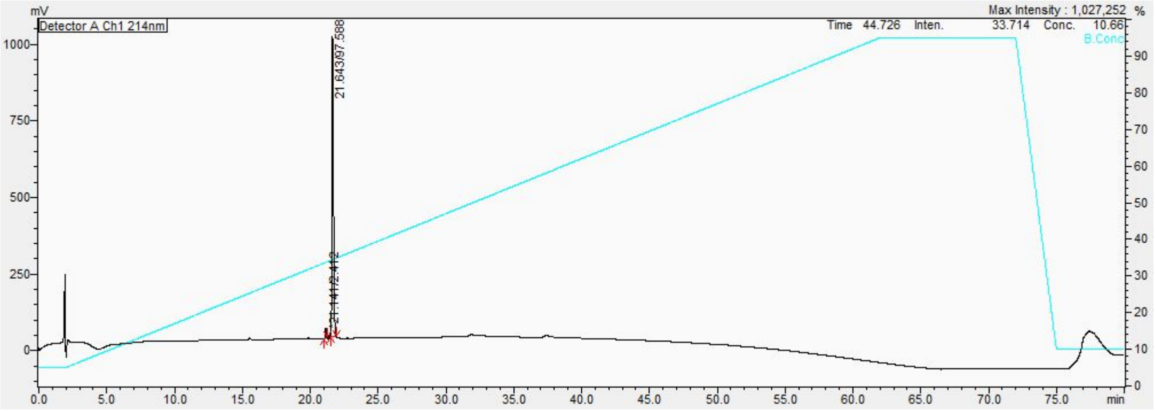

HPLC Data for Compound 53

(5-95% ACN 0.1% TFA in H_2O 0.1% TFA over 50 minutes)

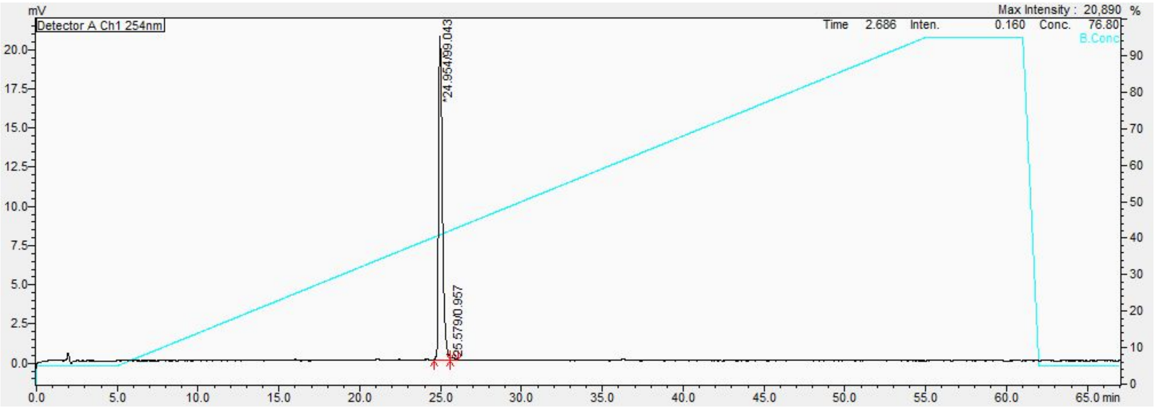

HPLC Data for Compound 54

(5-95% ACN 0.1% TFA in H_2O 0.1% TFA over 50 minutes)

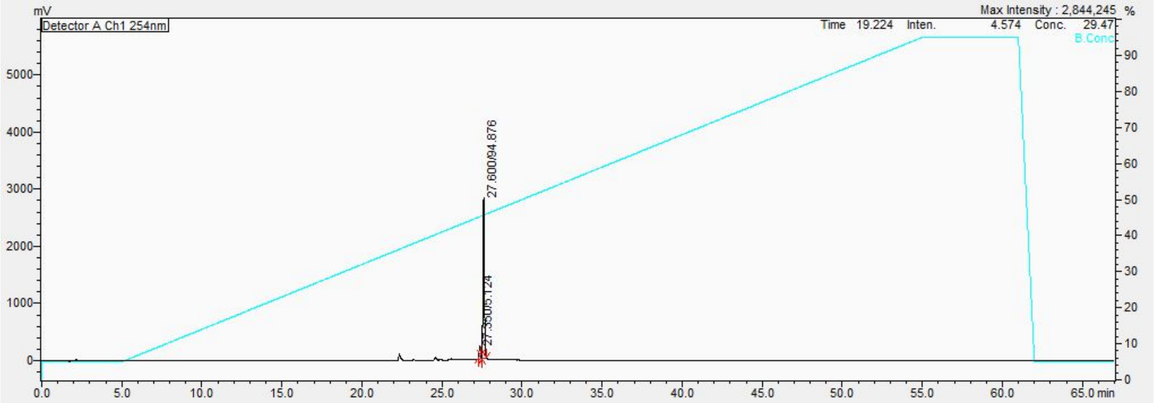

Supplement: Supplementary file 1 [file jm5c03342_si_001.pdf]
